# 2D/3D-QSAR Model Development Based on a Quinoline Pharmacophoric Core for the Inhibition of *Plasmodium falciparum*: An In Silico Approach with Experimental Validation

**DOI:** 10.3390/ph17070889

**Published:** 2024-07-04

**Authors:** Marcos Lorca, Gisela C. Muscia, Susana Pérez-Benavente, José M. Bautista, Alison Acosta, Cesar González, Gianfranco Sabadini, Jaime Mella, Silvia E. Asís, Marco Mellado

**Affiliations:** 1 Instituto de Química y Bioquímica, Facultad de Ciencias, Universidad de Valparaíso, Av. Gran Bretaña 1111, Valparaíso 2360102, Chile; marcos.lorca.c@gmail.com (M.L.); gianfranco.sabadini@postgrado.uv.cl (G.S.); 2 Departamento de Ciencias Químicas, Facultad de Farmacia y Bioquímica, Universidad de Buenos Aires, Junín 956, C1113AAB Ciudad Autónoma de Buenos Aires, Buenos Aires 1113, Argentina; gmuscia@ffyb.uba.ar; 3 Departamento de Bioquímica y Biología Molecular, Facultad de Veterinaria, Universidad Complutense de Madrid, 28040 Madrid, Spain; susipz@ucm.es (S.P.-B.); jmbau@ucm.es (J.M.B.); 4 Universidad Andres Bello, Facultad de Ciencias Exactas, Departamento de Ciencias Químicas, Viña del Mar 2531015, Chile; al.acosta@uandresbello.edu; 5 Departamento de Química, Universidad Técnica Federico Santa María, Av. España 1680, Valparaíso 2390123, Chile; cesar.gonzalez@usm.cl; 6 Centro de Investigacion, Desarrollo e Innovacion de Productos Bioactivos (CInBIO), Universidad de Valparaiso, Av. Gran Bretaña 1111, Valparaíso 2360102, Chile; 7 Facultad de Medicina y Ciencias de la Salud, Universidad Central de Chile, Santiago 8330507, Chile

**Keywords:** malaria, drug design, 2D- and 3D-QSAR models, quinoline synthesis, experimental validation, biocompatibility

## Abstract

Malaria is an infectious disease caused by *Plasmodium* spp. parasites, with widespread drug resistance to most antimalarial drugs. We report the development of two 3D-QSAR models based on comparative molecular field analysis (CoMFA), comparative molecular similarity index analysis (CoMSIA), and a 2D-QSAR model, using a database of 349 compounds with activity against the *P. falciparum* 3D7 strain. The models were validated internally and externally, complying with all metrics (q^2^ > 0.5, r^2^_test_ > 0.6, r^2^_m_ > 0.5, etc.). The final models have shown the following statistical values: r^2^_test_ CoMFA = 0.878, r^2^_test_ CoMSIA = 0.876, and r^2^_test_ 2D-QSAR = 0.845. The models were experimentally tested through the synthesis and biological evaluation of ten quinoline derivatives against *P. falciparum* 3D7. The CoMSIA and 2D-QSAR models outperformed CoMFA in terms of better predictive capacity (MAE = 0.7006, 0.4849, and 1.2803, respectively). The physicochemical and pharmacokinetic properties of three selected quinoline derivatives were similar to chloroquine. Finally, the compounds showed low cytotoxicity (IC_50_ > 100 µM) on human HepG2 cells. These results suggest that the QSAR models accurately predict the toxicological profile, correlating well with experimental in vivo data.

## 1. Introduction

According to the latest World Health Organization (WHO) report, there were 247 million cases of malaria in 2022. The illness is known to spread to a great extent in Africa. The first symptoms of malaria infection are a headache, temperature, and chills. Malaria is treatable and it can be prevented. It is transmitted by female Anopheles mosquitoes. In humans, the disease may be caused by five parasite species. Among them, the most dangerous are *Plasmodium falciparum* and *Plasmodium vivax*. The former prevails in Africa, and if its infection is left untreated, it may lead to death. Some individuals are more vulnerable to malaria than others. This includes children younger than 5 years old, pregnant women and patients with HIV/AIDS, as well as people with low immunity moving to areas with intense malaria transmission. Over the two peak years of the pandemic (2020–2021), COVID-19 led to about 13 million more malaria cases and 63,000 more malaria deaths [1].

Effective vector control and the use of preventive antimalarial drugs constitute some of the strategies to prevent the disease. Vector control is of the utmost importance since it contributes to preventing infection and reducing the spread of the disease. Preventive antimalarial chemotherapy is used in vulnerable populations at a high risk of contracting the disease, regardless of whether they have the disease or not. Moreover, the WHO recommends the use of the malaria vaccine (RTS,S/AS01) among children who live in areas where *P. falciparum* represents a major threat. The use of this vaccine results in a significant reduction in deaths or infections among this age group [1].

**Figure 1:**
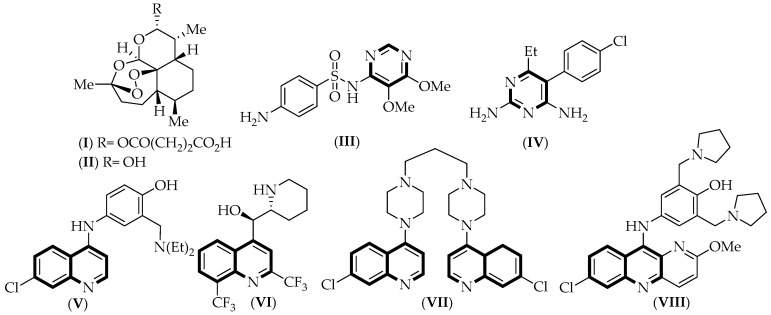
Chemical structure of the drugs used to treat malaria disease. Common structural aspects shared by all compounds are highlighted in black.

Several drugs have been used for the treatment of this disease, including artemisinin derivatives, like artesunate (**I**) and dihydroartemisinin (**II**); single-nitrogen heterocycles, such as sulfadoxine (**III**) and pyrimethamine (**IV**); and nitrogen-fused aromatic rings, like amodiaquine (**V**), mefloquine (**VI**), piperaquine (**VII**), and pyronaridine (**VIII**, Figure 1). The most effective treatment is obtained when these drugs are combined, for example, artesunate–amodiaquine, artesunate–mefloquine, artesunate–sulfadoxine–pyrimethamine, dihydroartemisinin–piperaquine, and the recent combination artesunate–pyronaridine [2]. However, antimalarial drug resistance to artemisinin-based combination therapy (ACT) has appeared, and the Greater Mekong subregion is the most affected. Therefore, new potent antimalarial molecules are required to fight drug resistance [2].

The effectiveness is limited by the emergence of multidrug-resistant parasites. The WHO Global Malaria Program (WHO/GMP) encourages and guides the development of products with a high impact on public health suitable for use in low- and middle-income countries [1]. Considering this, the design and synthesis of new antimalarial compounds is a challenge in medicinal chemistry. In this sense, bioinformatic tools are of great help in this field.

In recent years, several QSAR studies on quinolines active against *P. falciparum* have been published. Rhabori et al. [3] created models based on topological descriptors, such as atomic connectivity, carbon percentage, and density, without including experimental validation. Mahmud et al. [4] developed a QSAR equation for quinolines based on descriptors that are difficult to correlate with the biological mechanism of action, such as the compounds’ first ionization potential. Additionally, the dataset used by the authors was narrow, considering only 36 molecules and a narrow range of activities (less than 2 logarithmic units). Shallangwa et al. [5] constructed a QSAR equation for a total of 28 fluoroquinolines, but without considering the design of new molecules. None of the aforementioned studies included the synthesis and experimental validation of new molecules. A recent study by Vyas et al. [6] considered a database of 178 molecules from which they created CoMFA and CoMSIA studies, proposing 10 new molecules.

In the present work, we integrate the largest dataset of compounds available to date with activity against *P. falciparum*, comprising a total of 349 compounds with a range of activities spanning more than 8 logarithmic units. We developed CoMFA and CoMSIA models that are alignment-dependent, but we also present an alignment-independent 2D-QSAR model. Using these models, we synthesized and evaluated new compounds with promising activity on *P. falciparum* 3D7, showing a low toxicity profile in healthy cells. These results highlight that one of them showed activity comparable to that of chloroquine.

## 2. Results and Discussion

Building structure–activity relationship models (2D- and 3D-QSAR) is a fundamental tool for the rational development of new bioactive compounds with defined properties. The aforementioned method leads to a significant reduction in costs in the process of developing new antimalarial drugs as well as the time required to obtain lead compounds [7,8]. The theoretical models were developed using a database of 349 quinoline derivatives listed in Table S1. The molecules present a wide structural variability of substitution on the quinoline core. The biological activity spans more than ten logarithmic units. To obtain the best statistical values of CoMFA and CoMSIA models, a systematic sequential search was carried out (Table S2). The selection of the best model in both CoMFA and CoMSIA was based on the following standard criteria: (a) the highest possible value of r^2^, and (b) a value of q^2^ greater than 0.5 and a value of N less than 1/3 of the number of compounds [9,10]. Based on these criteria, the final selected models were CoMFA-SE (r^2^ = 0.932) and CoMSIA-SEHDA (r^2^ = 0.947). An external validation of the models was carried out, using metrics, such as q^2^ and r^2^_test_, as well as other parameters, like r^2^_0_, r’^2^_0_, k, and k’ (see Table 1). In addition, a *Y*-random test was performed (Table S4), ruling out the possibility that the developed models are the result of chance correlation [11].

**Figure 2:**
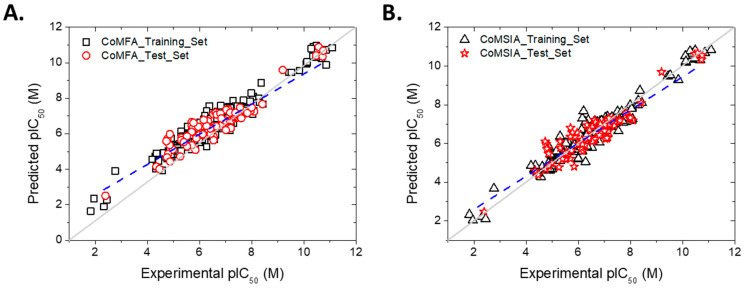
Plots of experimental versus predicted pIC_50_ values for the training (in black) and test sets (in red). For the test set, the regression line is shown as a dashed blue line. (**A**) CoMFA model. (**B**) CoMSIA model.

The experimental and predictive pIC_50_ values (−logIC_50_), with the predictions made by the selected CoMFA and CoMSIA models, are presented in Table S3. The experimental versus predictive activity plots for the best CoMFA and CoMSIA models were created to visualize whether there is an adequate linear distribution of the predictive results for both models (Figure 2). A good data distribution along the line *y* = *x* for both CoMFA and CoMSIA in the training and test sets was observed.

### 2.1. Contour Map Analysis

From the obtained CoMFA and CoMSIA models, it is possible to obtain a plot of contour maps in terms of different color-polyhedra around a molecule [12,13]. This allows us to identify the favorable/unfavorable characteristics for biological activity. In this way, the contour maps were plotted based on the best statistical models, CoMFA-SE and CoMSIA-SEHDA (CoMSIA-All, see Table S2). The projection of the contour map was carried out by using the highest active compound (*E*)-4-((*E*)-3-(4-bromophenyl)-3-oxoprop-1-en-1-yl)-2-(*tert*-butyl)-6-(((2-((7-chloroquinolin-4-yl)amino)ethyl)amino)methylene)cyclohexa-2,4-dien-1-one (**189**, pIC_50_ = 11.097) and the lowest active compound 4-(4-methoxyphenyl)-1-(4-methylquinolin-2-yl)-6-(*p*-tolyl)-1*H*-pyrazolo[3,4-b]pyridin-3-amine (**345**, pIC_50_ = 1.822).

#### 2.1.1. Steric Contour Map

**Figure 3:**
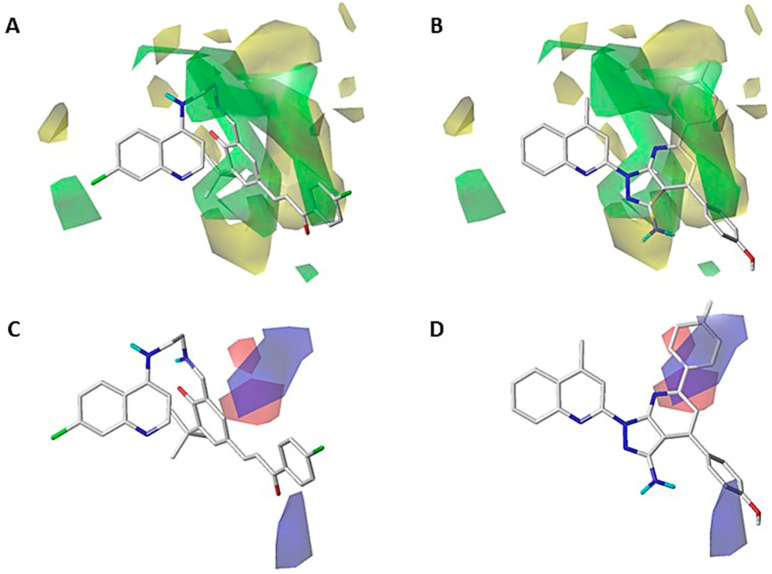
CoMFA steric (**A**,**B**) and electrostatic (**C**,**D**) contour maps around compounds **189** (**left**) and **345** (**right**), the most active and less active of the series, respectively. Color code: sterically favored (green) and disfavored (yellow). Electropositive favored (blue) and electronegative favored (red).

**Figure 4:**
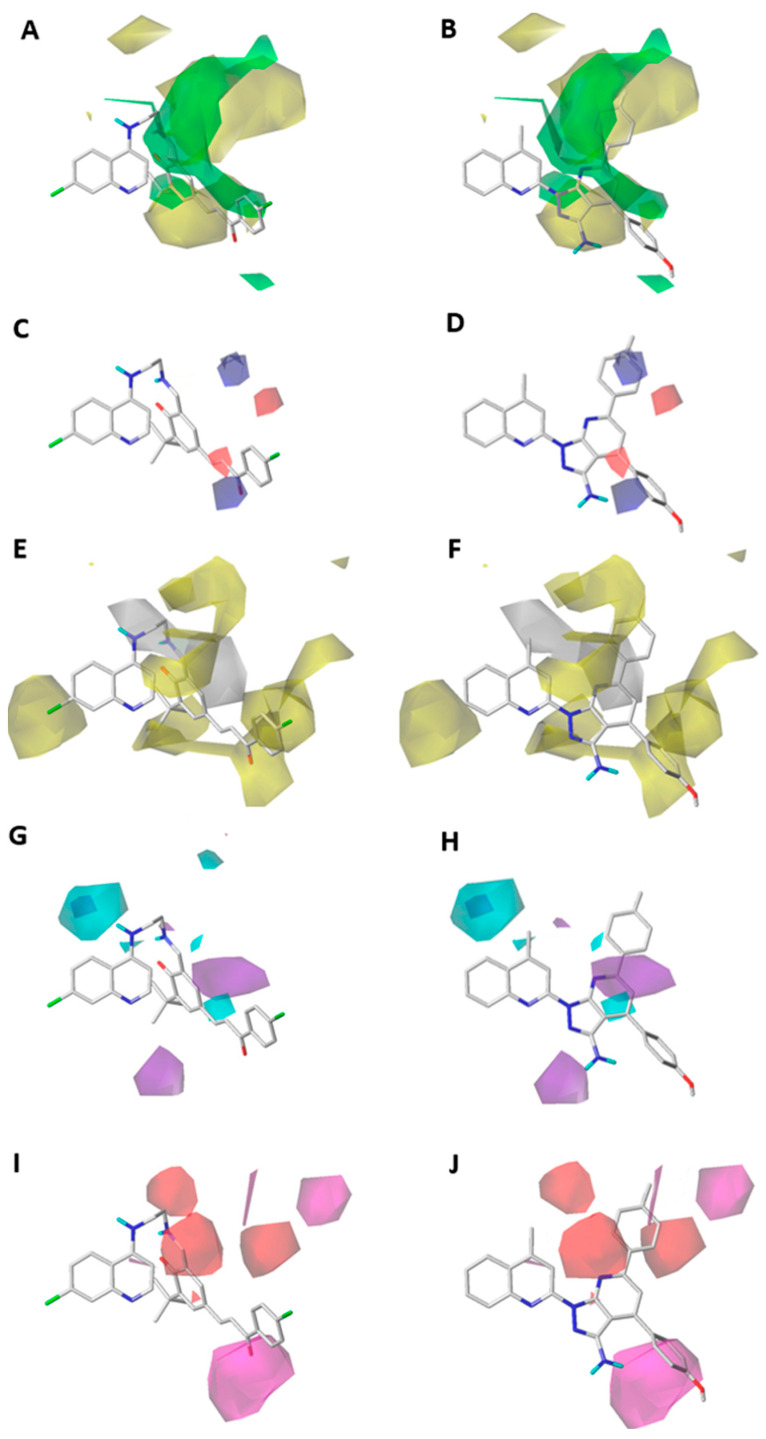
CoMSIA steric (**A**,**B**), electrostatic (**C**,**D**), hydrophobic (**E**,**F**), donor (**G**,**H**), and acceptor (**I**,**J**) contour maps around compounds **189** (**left**) and **345** (**right**), the most active and least active of the series, respectively. Color code: sterically favored areas are in green and disfavored areas are in yellow. Electropositive favored areas are in blue and electronegative favored areas are in red. Hydrophobic favored areas are in yellow and disfavored areas in grey. Donor and acceptor favored areas are in cyan and magenta, respectively, and donor and acceptor disfavored areas are in purple and red, respectively.

The steric maps for CoMFA and CoMSIA show some similarities between them (Figure 3A,B and Figure 4A,B), mainly close to positions 3 and 4 of the quinoline core. The contour map shows that a big substituent oriented to these positions favors the inhibition activity on *P. falciparum*, as seen in compound **189**. This molecule partially projects their ethylenediamine chain on the green polyhedron (Figure 3A,B and Figure 4A,B). This chain connects the fragments 4-quinoline and cyclohexa-2,4-dien-1-one. However, when a substituent bigger than a five-atom chain is bonded to positions 3 and 4 of the quinoline core, this can project partially on the yellow polyhedron placed next to the green polyhedron previously mentioned. This fact produces the decrease in the inhibitory activity on *P. falciparum*. In the case of position 2 of the quinoline core, a small green polyhedron is projected, showing that a bulky substituent, like a methyl group or similar, favors the inhibition of the *P. falciparum* (Figure 3A and Figure 4A). In this sense, when the substituent close to position 2 of the quinoline core is bigger than a methyl group (e.g., halogen or similar), this is partially projected on the yellow polyhedron. This was seen in compound **345**, which projects the fragments 4-methoxyphenyl and 6-*p*-tolyl on the yellow polyhedron previously mentioned (Figure 3B and Figure 4B).

Furthermore, there is another green polyhedral near position 7 of the quinoline core, which means that a bulky substituent in this zone favors the inhibition activity against *P. falciparum* (Figure 3A and Figure 4A). This is corroborated by the presence of a chlorine atom in the most active compound **189**, which is not present in the lowest active compound **345** (Figure 3B and Figure 4B).

#### 2.1.2. Electrostatic Contour Map

The electrostatic contour maps for CoMFA and CoMSIA are depicted in Figure 3C,D and Figure 4C,D. Some differences can be observed between them. In the case of the CoMFA contour map around compound (**189**, pIC_50_ = 11.097), a red polyhedron near the ring cyclohexa-2,4-dien-1-one (which is linked to the quinoline core through the ethylenediamine chain) indicates that an electron-rich atom favors the inhibition activity on *P. falciparum* (Figure 3C). This effect is concordant with the electron-donating property of the amine group bonded to the cyclohexa-2,4-dien-1-one ring. In contrast, in the least active compound (**345**, pIC_50_ = 1.822), there is a blue polyhedron projected on 4-methoxyphenyl and 6-*p*-tolyl (Figure 3D), which indicates that an electron-rich substituent causes a decrease in the inhibitory activity against *P. falciparum*. This is concordant with the increase in the electron density of the methyl and methoxy substituents.

On the other hand, in the CoMSIA contour map projected on the highly active compound (**189**), a red polyhedron is depicted around the α-carbon of the fragment α,β-unsaturated carbonyl bonded to the ring cyclohexa-2,4-dien-1-one (Figure 4C). This means that an electron-rich atom in this position favors the inhibitory activity on *P. falciparum*, while a blue polyhedron is placed on the carbonyl carbon from the previously mentioned fragment. This is concordant with the fact that the electron-deficiency on this atom favors the inhibitory activity on *P. falciparum*. In contrast, the CoMSIA contour map projected on the less active compound (**345**) showed two blue polyhedral shapes on the 4-methoxyphenyl and 6-*p*-tolyl electron-rich rings (Figure 4D). This can explain the low inhibitory activity on *P. falciparum* of this compound.

#### 2.1.3. Hydrophobic Contour Map

The hydrophobic contour map obtained from the CoMSIA model is in Figure 4E,F. A yellow polyhedron is shown close to position 7 of the quinoline core, which favors the inhibition activity on *P. falciparum*. This is concordant with the presence of the halogen atom in the most active compound (**189**, Figure 4E). However, in the less active compound (**345**), the substitution in position 7 of the quinoline is absent. In addition, on position 4 of the quinoline core is projected a white polyhedron, which means that a hydrophilic substituent favors the inhibitory activity on *P. falciparum*. For example, the presence of an ethylenediamine chain linked to position 4 of the quinoline (Figure 4E) of the highest active compound (**189**) would be favorable due to its polar properties. However, in the less active compound (**345**), a hydrophobic substituent, like a methyl group, is projected on this white polyhedron (Figure 4F), causing the decrease in the inhibitory activity on *P. falciparum*.

Furthermore, around positions 2 and 3 of the quinoline core, a yellow polyhedron is projected, showing that a hydrophobic substituent can increase the inhibitory activity of quinoline derivatives on *P. falciparum*. This effect is concordant with the orientation of the hydrophobic fragment cyclohexa-2,4-dien-1-one in the most active compound of the training set (**189**, Figure 4E). Meanwhile, when the substituent in position 3 of the quinoline core is bigger than three carbon atoms, the hydrophobic feature causes a decrease in the inhibitory activity on *P. falciparum* because of the insertion of the substituent in the white polyhedron next to the yellow polyhedron. This phenomenon is concordant with the orientation of a hydrophobic fragment of 1*H*-pyrazolo[3,4-b]pyridine and 4-methoxyphenyl rings of the less active compound of the training set (**345**, Figure 4F). In the case of position 2 of the quinoline core, a large yellow polyhedron is projected to the hydrophobic fragment (*E*)-3-(4-bromophenyl)-3-oxoprop-1-en-1-yl of the most active compound (**189**, Figure 4E). However, in this same zone a hydrophilic fragment of the derivative **345** is projected, concordant with the lower inhibitory activity of this compound on *P. falciparum*.

#### 2.1.4. Hydrogen-Bonding Donor Contour Map

The H-bond donor contour map is shown in Figure 4G,H. Near position 5 of the quinoline core is projected a big cyan polyhedron, which means that a polar hydrogen on this polyhedron favors the inhibition activity on *P. falciparum*. For example, the most active compound (**189**, Figure 4G) possesses a –NH group bonded to position 4 of the quinoline core in this region. In contrast, the less active compound (**345**) shows a methyl group bonded to position 4 of the quinoline core near this cyan polyhedron (Figure 4H). This is concordant with the low activity of this compound on *P. falciparum*.

On the other hand, approximately three-bonds distant from position 1 of the quinoline core, a purple polyhedron is projected, which means that a polar hydrogen causes a decrease in the inhibitory activity on *P. falciparum*. This is concordant with the projection of the hydrogen atoms from the –NH_2_ group (bonded to the fragment 1*H*-pyrazolo[3,4-b]pyridine) of the less active compound of the training set (**345**, Figure 4H).

#### 2.1.5. Hydrogen-Bonding Acceptor Contour Map

The H-bond acceptor contour map is depicted in Figure 4I,J. This map shows two red polyhedrons close to position 3 of the quinoline core, which means that a hydrogen-bonding acceptor causes the decrease in the inhibition activity on *P. falciparum*. This is observed in the projection of the nitrogen atom from 1*H*-pyrazolo[3,4-b]pyridine (Figure 4J), belonging to the least active compound of the training set (**345**), while these polyhedrons are projected mainly on alkyl fragments in the most active compound of the training set. In addition, to five-bonds distance from position 2 of the quinoline core, a magenta polyhedron is projected, which means that the hydrogen-bonding acceptor favors the inhibitory activity on *P. falciparum*. This is observed in the projection of the oxygen atom from the (4-bromophenyl)-3-oxoprop-1-en-1-yl fragment of the compound **189**. In contrast, on the same magenta polyhedron is the –NH_2_ group of the 1*H*-pyrazolo[3,4-b]pyridine-3-amine fragment of compound **345**, which is concordant with the low inhibitory activity of this compound (Figure 4J).

#### 2.1.6. 2D-QSAR Model

In order to obtain information without dependence of the conformation and molecular alignment of the compounds, we carried out a 2D-QSAR study of the compounds. After testing the combination of multiple physicochemical and topological variables, we found the following equation:

**pIC_50_** = 0.05891**AlogP** + 0.00062**AlogP^2^** + 0.30435**nCl** + 0.0591**nF** + 0.11481**nBase** + 0.27**nHBDon** + 0.00755**TopoPSA** + 0.57562**nT6HeteroRing** − 0.00013**AMR** − 0.00095**VABC** + 4.45138 (1)

**Figure 5:**
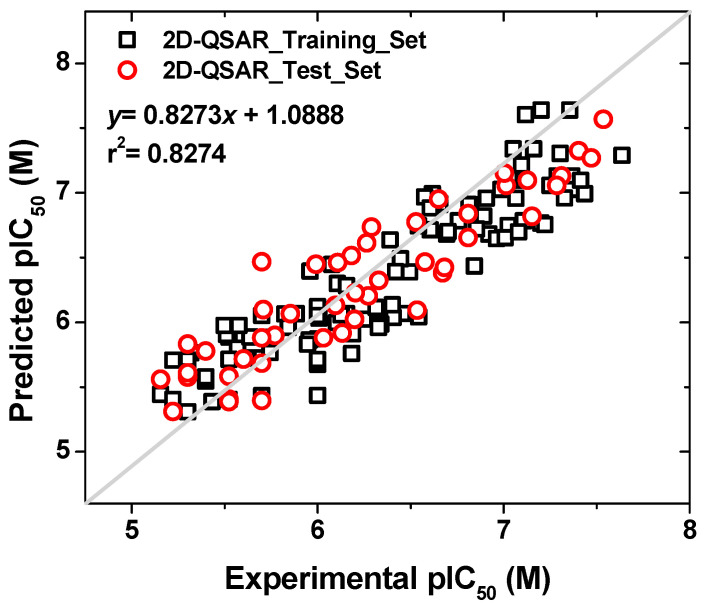
Plot of experimental versus predictive activities (pIC_50_) for the set of compounds used in the 2D-QSAR model.

The model obtained was built for a subset of 156 compounds from the database studied. A total of 110 compounds were used as the training set, and 46 were used as the test set. This is equivalent to a 70:30 split of the data set. The model presented a r^2^ = 0.8274 for the training set, and a predictive r^2^ = 0.8454 for the test set. Figure 5 shows the regression graph of the values. The activities covered were in the range of 5 to 8 (−logIC_50_).

From Equation (1), we can see that the inhibitory activity depends favorably on the lipophilicity (ALogP), the number of chlorine atoms (nCl), the number of fluorine atoms (nF), the number of basic nitrogen atoms (nBase), the number of hydrogen-bonding atoms (nHBDon), the topological polar surface area (TopoPSA), and the number of six-membered rings (includes counts from fused rings) containing heteroatoms (N, O, P, S, or halogens) (nT6HeteroRing). Moreover, the descriptors that contribute negatively to the activity are molar refractivity (AMR) and Van der Waals volume with atomic and bond contributions (VABC). This information is consistent with the 3D-QSAR models that establish that the presence of hydrophobic groups in R_2_ and R_3_ is favorable for activity. On the other hand, the increase in activity in the presence of atoms, such as chlorine, fluorine, and basic nitrogen atoms, may be due to the increase in the lipophilicity (-F or -Cl), and the hydrogen-bonding acceptor (-N) properties of the compounds. The presence of chlorine is a greater contributor than fluorine (0.30435 versus 0.0591), but larger halogens, such as bromine and iodine, should be avoided since they would increase the AMR and VABC values. Furthermore, the presence of additional heterocycles (nT6HeteroRing) favors the activity. This is consistent with the contour maps of the 3D-QSAR models in which a green polyhedron can be seen over the additional cycles connected to the quinoline nucleus. It is likely that the presence of additional rings translates into π-stacking interactions with the potential target. Recent studies show that some drugs, such as mefloquine, interact in the ribosomal 80s subunit [14], while other drugs, such as chloroquine, block the action of heme polymerase [15], so the presence of additional aromatic rings favors π-stacking type interactions with nitrogenated bases or with heme groups. Given that the nT6 descriptor has the most contributing coefficient to the activity, in the design of new compounds we took into account the incorporation of additional rings in the structure, which was reflected in compounds **350**–**354**. Among them, the designed compounds **353** and **354** were the ones with the highest experimental inhibitory activity.

#### 2.1.7. Applicability Domain of the Models

**Figure 6:**
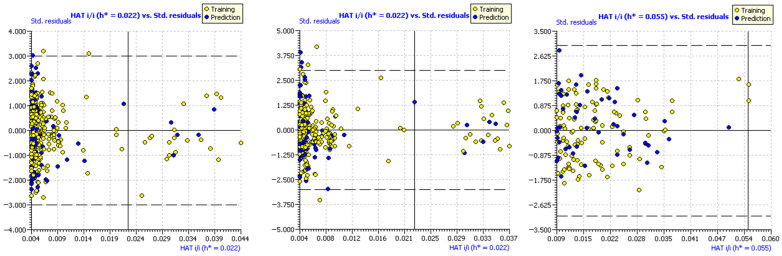
Williams plots for CoMFA (**left**), CoMSIA (**center**), and 2D−QSAR (**right**). The Hat value versus the standardized residuals are depicted in each case. Dotted lines show the limit 3σ standard deviation. Points outside those lines are outliers. The vertical line is the warning h* leverage value.

The evaluation of the applicability domain of the models was carried out by constructing Williams plots (Figure 6). In these diagrams, the leverage (Hat value) of each compound is plotted against its standardized residual. The dashed lines indicate the thresholds for the standard deviation of the models (3σ). Compounds above or below these dashed lines are considered outliers. The CoMSIA model has a total of four outliers in the training set, and three in the test set (error rate of 0.86% relative to the total number of compounds studied). The CoMFA model did not present any outliers in the test set and presented only two values at the standard deviation limit in the training set. Nevertheless, the best fit in terms of standard deviation was achieved with the 2D-QSAR model, which did not present any outliers. In each graph, a vertical line is shown corresponding to the leverage limit. Compounds from the training set above the value h* exert a significant influence on the regression. In CoMFA, there are 18 compounds above h*, and in CoMSIA, there are 19. This accounts for 5% of the total compounds studied. On the other hand, the 2D-QSAR model presents two training compounds with the same value as h*. Based on this, it can be concluded that the 2D-QSAR model exhibits a better distribution and statistical contribution of each compound to the regression. For compounds from the test set that exceed the value h*, this means that the prediction of biological activity has had a significant extrapolation. Therefore, the prediction of pIC_50_ in those cases should be seen with caution. Both CoMFA and CoMSIA had five compounds outside the applicability domain, which accounts for 1.4% of the total compounds. The 2D-QSAR model had no test set compounds outside the applicability domain.

#### 2.1.8. Summary of the Principal Results Based on the Theoretical Models

**Figure 7:**
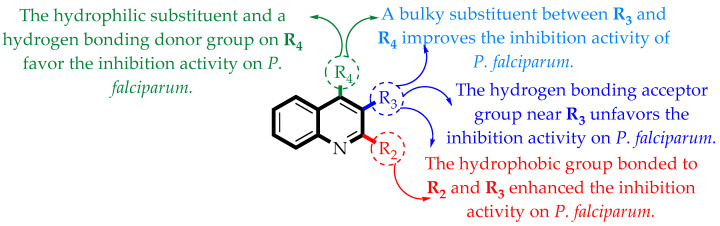
Summary of the main structure–activity relationships found in this study.

From the QSAR models, a set of important structural information has been extracted. With this information, the synthesis of some compounds was performed. This allowed the experimental validation of the QSAR models. These features are summarized in Figure 7.

#### 2.1.9. Limitations of the Models

When working with integrated databases containing numerous molecules, as in the present study, one of the major challenges is achieving appropriate molecular alignment to obtain models with good predictive statistics. In this case, this was addressed using automated alignment via the distill rigid method. This method aims to minimize the RMSD to maximize molecular overlap. Alternatively, we evaluated the creation of a 2D-QSAR model, which is independent of molecular alignment, and found its predictive statistics to be appropriate. The information provided by the descriptors in the equation was consistent with the contributions from the 3D models. It is worth noting that interpreting 3D contour maps is often challenging and can lend itself to subjective interpretations if careful visualization of the polyhedra is not undertaken. Such uncertainties can be addressed by using other methods that are independent of molecular alignment, such as the calculation of “circular fingerprints” from Pipeline Pilot and the Carhart atom pair descriptor. These strategies allow the visualization of colored molecular regions as favorable or unfavorable, facilitating a more direct interpretation in terms of structure–activity relationships. Additionally, such methods can be carried out using the random forest method, which often achieves better regression values (r^2^) compared to PLS [16].

### 2.2. Experimental Validation of the Theoretical Models

According to Table S2, the greatest contribution in the CoMFA model is the steric field with 95.1%, while in the CoMSIA model, the most important contributions are the steric, hydrophobic, hydrogen-bonding donor, and hydrogen-bonding acceptor fields, with 96.1% of the total assessed. To assess the importance of the size of substituents with steric characteristics on position R_4_ of the quinoline core, twelve compounds were designed (**350**–**360**) with a methyl group bonded to the position previously mentioned. This substitution will allow to check if the size of the methyl group of the 4-methylquinoline derivatives can affect the inhibitory activity against *P. falciparum*. In addition, to determine the importance of the size and the orientation of the hydrophobic fragment close to the position R_2_ of the quinoline, different rings were selected (e.g., substituted phenyl (**350**–**351**), naphthyl (**352**), phenanthrenyl (**353**), and indolyl (**354**)). The rings were bonded through one rotatable bond to allow projection into the hydrophobic polyhedron, which is favorable for the inhibitory activity on *P. falciparum*. In this way, five derivatives were considered without rotatable bond (**355**–**359**), yielding different quinoline-fused rings with conformational restriction to confirm if the loss of conformational freedom improves the inhibitory activity on *P. falciparum*. Finally, close to position R_3_, a hydrogen-bonding acceptor cannot improve the inhibition activity on *P. falciparum* of the 4-methylquinoline derivatives. To test this hypothesis, one derivative was designed with a carbonyl group bonded to the quinoline core (**360**).

**Scheme 1:**
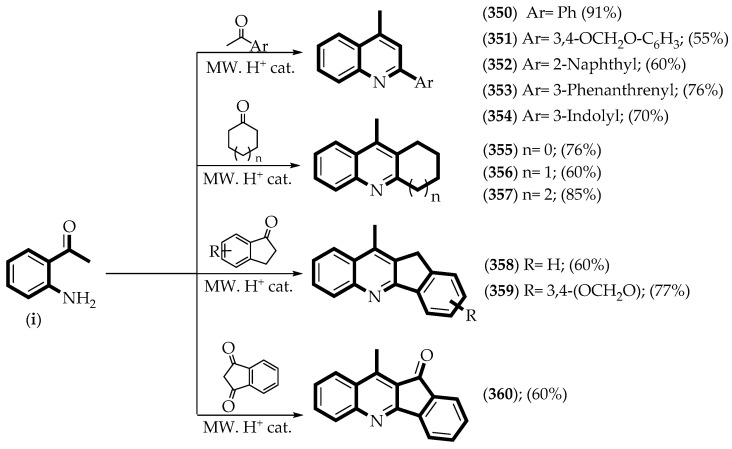
Synthetic route to obtain the experimental test set of QSAR models. MW = microwave irradiation. The yield of each product is displayed in parentheses. (**i**) 2-aminoacetophenone.

To build the quinoline fragment with the features previously defined, the Friedländer reaction was carried out, catalyzed by trifluoracetic acid (TFA), under microwave irradiation (MW, Scheme 1). In this reaction, one molar equivalent of 2-aminoacetophenone (**i**) and one molar equivalent of the proper ketone were mixed and irradiated with microwaves (1–7 min), obtaining the 2-arylquinoline derivatives (**350**–**354**), the acridine derivatives (**355**–**357**), and the 11*H*-indeno[1,2-b]quinoline derivatives (**357**–**360**) with moderate to good yields (55–91%).

The synthesized compounds were identified by spectroscopic techniques (IR and NMR). In the case of the infrared spectrum, the symmetric and asymmetric stretching bands of the –NH_2_ group are not seen (υ ~3200 cm^−1^), as well as the stretching band of the carbonyl group (CO, υ ~1650 cm^−1^) of the ketone used for the Friedländer reaction, confirming the condensation between the 2-aminoacetophenone (**i**) and the ketone fragment. In addition, in the case of the ^1^H-NMR spectrum of compounds **350**–**354**, all of them have a single signal to the low field (δ ~8.3 ppm) that corresponds to one hydrogen atom of position 3 of the quinoline core, while compounds **355**–**359** show a doublet signal of the -CH_2_- group bonded to the same position (δ ~3.5 ppm). In contrast, compound **360** has a carbonyl group bonded to position 3 of the quinoline core, which shows a small signal at δ = 192 ppm in ^13^C-NMR.

After synthesizing and characterizing compounds **350**–**360**, they were assessed as growth inhibitors of *P. falciparum*, and these results are summarized in Table 2. According to the obtained values, the CoMFA model shows an overestimated predictive activity against *P. falciparum*, that is to say, the residual values are higher than one logarithmic unit, except for compound **353**, which has a residual value Δ = −0.199. This trend can be linked to the low equilibrium between the contributions of the steric and electrostatic fields (95.1% and 4.9%, respectively, Table S2). In other words, even though the designed compounds projected part of their molecular structure on the steric green polyhedron, the high contribution of this field causes the overvaluing of the predicted inhibitory activity against *P. falciparum* (Figure 7). On the other hand, the CoMSIA model shows a balanced trend between the steric, hydrophobic, donor, and acceptor hydrogen-bonding fields (15.7%, 28.5%, 23.6%, and 28.3%, respectively), causing the predicted inhibitory activity against *P. falciparum* to show a better fit with the experimental values. However, the predictions of the 2D-QSAR model were particularly good. All estimates had a deviation of less than one logarithmic unit. Additionally, the root mean squared error of prediction (RMSEP) and mean absolute error (MAE) metrics were calculated for each model. The RMSEP metric measures the overall deviation of the models. The lower the value, the less tendency the model has to deliver drastic deviations in the predictions, whether positive or negative. The best models were CoMSIA and 2D-QSAR, while the CoMFA model has the worst metric. The MAE metric is an estimate of the point-to-point deviation of each measurement. In this case, the 2D-QSAR model generates the best particular estimates, followed by the CoMSIA model.

**Figure 8:**
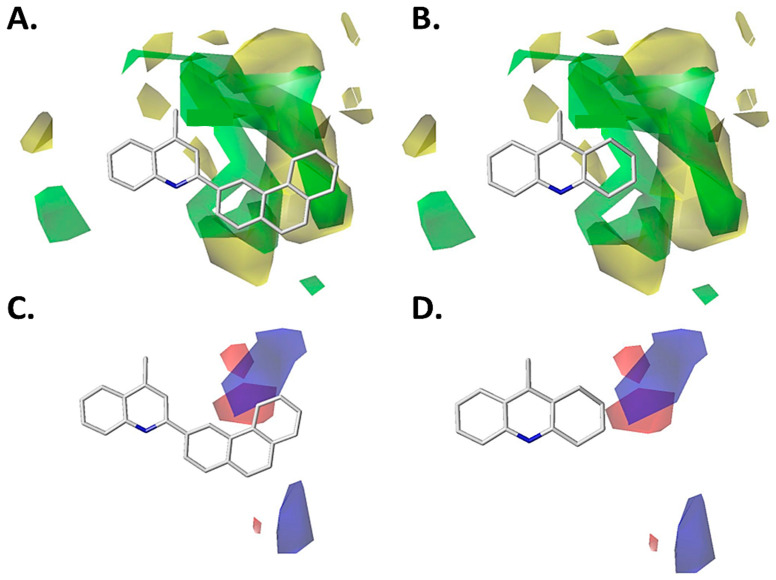
CoMFA models projected on the most active designed compound (**left**) and the least active designed compound (**right**). (**A**) Steric CoMFA model for compound **353**. (**B**) Steric CoMFA model for compound **356**. (**C**) Electrostatic CoMFA model for compound **353**. (**D**) Electrostatic CoMFA model for compound **356**. Color code: sterically favored areas are in green and disfavored areas are in yellow. Electropositive favored areas are in blue and electronegative favored areas are in red.

**Figure 9:**
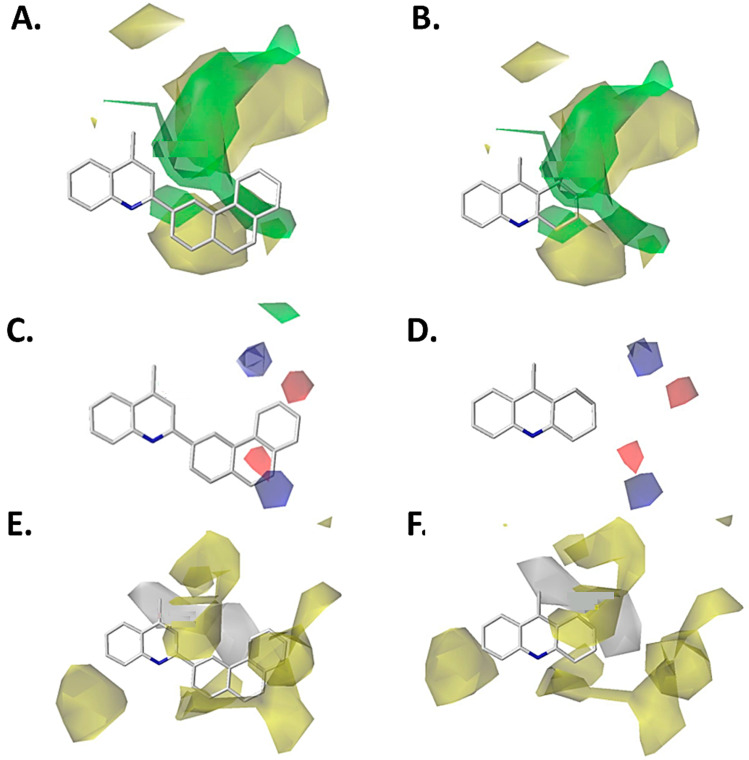
CoMSIA models projected on the most active compound designed (**left**) and the low active compound designed (**right**). (**A**) Steric CoMSIA model for compound **353**. (**B**) Steric CoMSIA model for compound **356**. (**C**) Electrostatic CoMSIA model for compound **353**. (**D**) Electrostatic CoMSIA model for compound **356**. (**E**) Hydrophobic CoMSIA model for compound **353**. (**F**) Hydrophobic CoMSIA model for compound **356**. Color code: sterically favored areas are in green and disfavored areas are in yellow. Electropositive favored areas are in blue and electronegative favored areas are in red. Hydrophobic favored areas are in yellow and disfavored areas in grey.

The compounds 4-methyl-2-(phenanthrene-3-yl)quinoline (**353**) and 9-methyl-1,2,3,4-tetrahydroacridine (**356**), which are the most and the least active designed compounds, respectively, are depicted in Figure 8 (CoMFA) and Figure 9 (CoMSIA). It can be seen that the phenanthrene-3-yl fragment is near to the green polyhedron in the steric map (Figure 8A and Figure 9A). This is concordant with the high inhibitory activity on *P. falciparum* of this compound, while compound **356** is partially projected on the green polyhedron, mainly the cyclohexane fragment (Figure 8B and Figure 9B), causing the decrease in the inhibitory activity on *P. falciparum*.

Additionally, the electrostatic map obtained from the CoMFA model is depicted for compound **353**, showing that close to positions 5 and 6 of the phenanthrene-3-yl fragment, a blue polyhedron is projected (Figure 8C). This means that an electron-deficient substituent favors the inhibition activity against *P. falciparum*. This is concordant with the electron-deficient characteristic of the carbon on position 6 of the phenanthrene-3-yl fragment due to the electron-withdrawing resonance effect of the quinoline core. Meanwhile, in compound **356**, the same polyhedron is near position 2 of the 1,2,3,4-tetrahydroacridine core (Figure 8D), which has neutral properties. Therefore, this feature does not cause the increasing or decreasing inhibition activity on *P. falciparum*. In addition, the electrostatic map of the CoMSIA model, shows a similar trend to the CoMFA model (Figure 9C,D), except that the carbon atom in position 9 of the phenanthrene-3-yl fragment has an electron-deficient property caused by the electron-withdrawing effect of the quinoline core. This carbon atom is near the blue polyhedron (Figure 9C), which is concordant with the high inhibitory activity on *P. falciparum*.

On the other hand, the hydrophobic map obtained from the CoMSIA model shows that compound **353** projects the phenanthrene-3-yl fragment on the yellow polyhedron, as well as the carbon atoms from positions 2 and 3 of the quinoline core (Figure 9E). Both features contribute to the increase in the inhibitory activity of this compound on the *P. falciparum*. By contrast, the quinoline derivative **356** only projects a part of the cyclohexane fragment of the 1,2,3,4-tetrahydroacridine core on a yellow polyhedron, explaining the diminishing inhibitory activity on *P. falciparum* of this designed compound.

The contour maps corresponding to the H-bond donor and acceptor properties show proximity to the chemical structure of compounds **353** and **356** (Figure S3). However, the compounds previously mentioned do not have substituents with these features, although compounds **354** and **351** have hydrogen donor and acceptor groups (Figure S4). In this sense, compound **354** (pIC_50_ = 5.281) shows the –NH group of the 3-indolyl fragment close to the purple polyhedron, resulting in an unfavorable effect on the inhibition activity against *P. falciparum*. However, when there is a rotation of the indole fragment, this causes the –NH projection group of the 3-indolyl fragment on the cyan polyhedron, favoring the inhibition activity on *P. falciparum* (Figure S4G). By contrast, compound **351** (pIC_50_ = 4.450) projects one oxygen atom from the benzo[*d*][1,3]dioxol fragment on the cyan polyhedron. This means a hydrogen-bonding acceptor group causes the decrease in the inhibitory activity on *P. falciparum* (Figure S4H), which is concordant with the experimental evidence shown in Table 2.

The hydrogen-bonding acceptor map obtained from the CoMSIA model and overlaying on compound **354** shows the –NH group of the 3-indolyl fragment close to the magenta polyhedron, in which a hydrogen-bonding acceptor group favors the inhibitory activity against *P. falciparum* (Figure S4I). By contrast, compound **351** projects one oxygen atom of the benzo[*d*][1,3]dioxol fragment on the purple polyhedron, concordant with the low inhibition activity on *P. falciparum* of this compound. In addition, if the rotation of the substituent through the bond with the quinoline core is considered, the –NH group of the 3-indolyl fragment of compound **354** is not near any polyhedron. However, the benzo[*d*][1,3]dioxol fragment of compound **351** is projected on the purple polyhedron, causing a decrease in the inhibitory activity against *P. falciparum*. This is concordant with the experimental values obtained from Table 2.

### 2.3. Evaluation of Physicochemical and Pharmacokinetic Properties

The physicochemical properties of the selected compounds obtained in the experimental validation of the 3D-QSAR models were analyzed in silico to find any Lipinski’s rule violation. For this purpose, we used the SwissADME web tool [17]. Furthermore, the pharmacokinetic properties were estimated in the AdmetSAR website [18,19], and they were compared with the profile of chloroquine (Table 3).

Through the use of the SwissADME online platform, it can be determined whether the compounds comply with Lipinski’s rule of five, which establishes that drug candidates can present low absorption and permeability if they violate at least two rules. Compound **353** (the most active of the experimental set) shows one violation of Lipinski’s rule of five (Table 3). Compound **353** has a Moriguchi LogP value of MLogP = 4.98 [20], which is above the recommended value of <4.5. Moreover, the compounds that are new drug candidates must present good bioavailability and good permeation through membranes, hence the importance of predicting the permeability of these derivatives. For example, topological polar surface area (TPSA) is an important descriptor for characterizing drug absorption, where a TPSA < 140 Å is an indicative of good intestinal absorption [21]. In addition, human colon adenocarcinoma cells (Caco-2) permeability, intestinal absorption, bioavailability, and penetration through the BBB were measured [22].

### 2.4. Cytotoxic Properties

**Figure 10:**
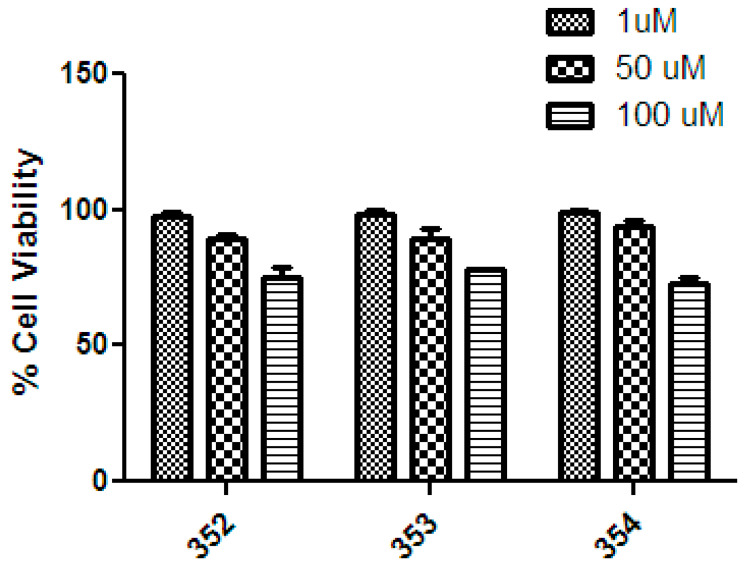
Cell viability of compounds **352**–**354** at three different concentrations (1, 50, and 100 μM) on the HepG2 cell line. For reference, the % Cell viability values for chloroquine are 1 μM = 90%; 50 μM = <10%; 100 μM = 0% [25].

One of the limitations of anti-malaria drug administration, e.g., using chloroquine, is the fact that this drug causes undesirable side effects, such as retinopathy and heart disease, among others [23]. On the other hand, the compound artesunate (**I**) is used for malaria treatment; however, this compound also has undesirable side effects, like hepatic cell growth inhibition [24]. Then, as mentioned above, the three most active compounds (**352**–**354**) were assessed as cytotoxic agents in a model of liver cells using the cell line HepG2. The results are summarized in Figure 10.

This figure shows that compounds **354**–**354** have low cytotoxic effects on HepG2 cells at all concentrations measured (1, 50, and 100 µM), between 75% to 100% of cellular viability. Therefore, all compounds assessed showed half-inhibition concentration (IC_50_) values higher than 100 μM. In consequence, these results suggest that compounds **352**–**354** have good biocompatibility, with selectivity index (IC_50_ HepG2/IC_50_ *P. falciparum*) values of >6.9, >66.2, and >10.1, respectively.

From a structural standpoint, compounds **352**–**354** could be less toxic than derivatives, such as artesunate, due to the greater stability of the compounds against metabolic degradation. The endoperoxide fragment of artesunate is highly reactive and can cleave to form reactive oxygen species (ROS) which cause cellular damage. Compounds **352**–**354** lack cytotoxic functional groups, such as endoperoxides, or electrophilic functionalities, like aldehydes, which are capable of generating protein damage through covalent bonding.

## 3. Materials and Methods

### 3.1. Theoretical Models

3D-QSAR studies were performed with Sybyl X software, version 1.2 (Tripos Inc., St. Louis, MO, USA). The general calculations were performed according to our previous reports [26,27,28]. Briefly, every compound was drawn in ChemDraw (PerkinElmer Inc., Shelton, CT, USA), and the structures were minimized by using the Tripos force field. Then, the MMFF94 charges were calculated for each atom. For the alignment of all compounds, the rigid alignment technique that minimizes RMS distance was used (Figure S1) through the distill rigid protocol implemented in Sybyl. The minimum energy conformation of the quinoline core was used as the common scaffold for alignment. The final models were performed on a set of 349 compounds with inhibition activity on *P. falciparum* 3D7. The biological activity was measured as half-inhibition concentration (IC_50_), as previously reported [29,30,31,32,33,34,35,36,37,38,39,40,41,42,43,44,45,46,47,48,49,50]. More details about the theoretical models are in the Supplementary Materials.

Regarding the 2D-QSAR, a series of physicochemical and topological descriptors were calculated for each molecule in the database. The structures were previously converted to SMILES code [51] and were then entered into the PaDEL-Descriptor program (Pharmaceutical Data Exploration Lab., Kent Ridge, Singapore) [52]. A total of 663 descriptors were calculated for each molecule. Subsequently, a search for correlations between the calculated descriptors and the inhibitory activity expressed as pIC_50_ was carried out. The combinations of descriptors were made, taking into account the structural characteristics of the molecules and the physicochemical properties that contributed to an interpretation of the mechanism of action of the compounds (the fifth principle of the OECD), and the model refinement was carried out according to the previous report [53]. A final equation with good statistics was obtained for a total of 156 compounds. The compounds were randomized in the training set (110 molecules, 70%) and the test set (46 molecules, 30%). Finally, the experimental versus predictive activities for training and test sets were plotted in Excel, where the regression equation and the r^2^ value for the training and test sets were determined.

### 3.2. Synthesis of Compounds

#### 3.2.1. Instrument and Chemicals

The structures of the synthesized compounds were established through their ^1^H and ^13^C-NMR, MS and IR spectra. Melting points were determined in a capillary Electrothermal 9100 SERIES-Digital apparatus and are uncorrected (United Kingdom). Here, ^1^H and ^13^C-NMR spectra were obtained using a Bruker 600 spectrometer (Fällanden, Switzerland). The operating frequencies for protons and carbons were 600 and 151 MHz, respectively. The chemical shifts (δ) were given in ppm. IR spectra were recorded using an FT-IR Thermo Scientific (Madison, WI, USA) from KBr discs. Mass spectra were measured on MS/DSQ II Thermo Scientific DPC (St. Louis, CO, USA) or Agilent Q-TOF 6520 model (Santa Clara, CA, USA). Elemental analysis data for synthesized compounds were consistent with calculated values. Analytical TLCs were performed on a DC-Alufolien Kieselgel 60 F254 Merck. Microwave-assisted reactions were carried out in a glass vial G10, Anton Paar Monowave 400 (Serial number: 81920884, instrument software version: 4.10.9376.7, Graz, Austria).

The following reagents were all purchased from Sigma-Aldrich/Merck (Darmstadt, Germany) and used without further purification: 2-aminoacetophenone (reagent grade, 98%), acetophenone (reagent grade, 99%), 3′,4′-(methylenedioxy)acetophenone (reagent grade, 98%), 3-acetylphenanthrene (reagent grade, 90%). 3-acetylindole (reagent grade, 98%), cyclopentanone (reagent grade, ≥99%), cyclohexanone (reagent grade, ≥99%), cycloheptanone (reagent grade, 99%), 1-indanone (reagent grade, ≥99%), 5,6-methylenedioxy-1-indanone (reagent grade, ≥95%), 1,3-indandione (reagent grade, 97%), hydrochloric acid (HCl, reagent grade 37% *w*/*w*), trifluoroacetic acid (TFA, reagent grade, ≥99%), benzene (analytical reagent grade), dichloromethane (analytical reagent grade), *iso*propanol (analytical reagent grade), cyclohexane (analytical reagent grade), ethylacetate (analytical reagent grade), and ethanol (analytical reagent grade). Finally, the 2-acetonaphthone was obtained according to our previous report [54].

#### 3.2.2. Synthetic Procedures

A neat mixture of 1 mmol (0.12 mL) of 2-amino-acetophenone (**i**) and 1 mmol of the corresponding methyl-arylketone (or cyclanone) with 0.10 mL TFA (or HCl cc) was subjected to MW irradiation at 170 °C and 850 W. The course of the reaction was monitored by TLC. The mixture was cooled to room temperature to give a solid product which was then crystallized from the appropriate solvent.

4-Methyl-2-phenylquinoline (**350**). Reaction time 6 min, cat. HCl. Pale orange powder (91% yield, from benzene), mp 208–210 °C. IR (cm^−1^) ʋ = 2922, 2852, 1643,1593, 1479, 1253, 1095, 954, 796, 761. ^1^H-NMR (CDCl_3_): δ 9.66 (1H, d, *J* = 8.5 Hz), 8.36 (1H, d, *J* = 3.2 Hz), 8.17 (1H, d, *J* = 8.3 Hz), 8.01 (1H, td, *J* = 8.4, 6.9, 1.2 Hz), 7.92 (1H, s), 7.84 (1H, t, *J* = 7.7 Hz), 7.67–7.62 (3H, m), 3.02 (3H, s). ^13^C-NMR (CDCl_3_): δ 156.02, 154.05, 138.99, 134.15, 133.13, 130.19, 130.02, 129.57, 129.35, 129.35, 128.32, 127.01, 124.14, 123.60, 121.54, 20.33. MS (M^+^+H) 220.11208 a.m.u., calc 220.11262 a.m.u., Δ = 0.00018 a.m.u. All spectroscopic information is concordant to the previous report [54].

2-(Benzo[*d*][1,3]dioxol-5-yl)-4-methylquinoline (**351**). Reaction time 4 min, cat. HCl. Beige powder (55% yield, from CH_2_Cl_2_), mp 131–133 °C. IR (cm^−1^) ʋ = 2951.6, 1740.7, 1604.0, 1340.9, 1100.9, 1031.9, 939.1. ^1^H-NMR (DMSO-d_6_) δ 8.50 (1H, d, *J* = 4.3 Hz), 8.29 (1H, d, *J* = 8.2 Hz), 8.26 (1H, s) 8.02 (1H, t, *J* = 7.4 Hz), 7.92 (1H, d, *J* = 1.8 Hz), 7.88 (1H, dd, *J* = 8.2, 1.8 Hz), 7.83 (1H, t, *J* = 7.4 Hz), 7.23 (1H, d, *J* = 8.2 Hz), 6.22 (2H, s), 2.91 (3H, s). ^13^C-NMR (DMSO-d_6_): δ 154.31, 153.81, 148.75, 136.46, 133.18, 128.54, 127.07, 125.56, 121.35, 109.01, 109.00, 102.71, 19.67. MS (M^+^) 264.1 a.m.u., calc. 264.3, Δ = 0.2 a.m.u. All spectroscopic information is concordant to the previous report [54].

4-Methyl-2-naphthalen-2-yl-quinoline (**352**). Reaction time 4 min, cat. TFA. Pale yellow powder (83% yield, from i-PrOH), mp 241–243 °C. IR (cm^−1^) ʋ = 3032.6, 1931.2, 1635.4, 1258.4, 821.2, 746.6. ^1^H-NMR (DMSO-d_6_): δ 8.90 (1H, d, *J* = 1.9 Hz), 8.46 (1H, d, *J* = 8.4 Hz), 8.42 (1H, s), 8.40 (1H, dd, *J* = 6.7, 1.9 Hz), 8.32 (1H, d, *J* = 8.4 Hz), 8.19 (1H, d, *J* = 8.6 Hz), 8.16–8.13 (1H, m), 8.09–8.05 (1H, m), 8.02 (1H, t, *J* = 7.6 Hz), 7.84 (1H, t, *J* = 7.6 Hz), 7.70–7.66 (2H, m, 2H), 2.95 (3H, s). ^13^C-NMR (DMSO-d_6_): δ 178.19, 154.67, 134.47, 133.12, 129.46, 129.19, 128.56, 128.40, 128.22, 127.64, 127.60, 127.41, 127.38, 125.46, 125.43, 121.53, 121.50, 109.99, 19.59. MS (M^+^+H) 270.12773 a.m.u., calc. 270.12827 a.m.u., Δ = 0.00054 a.m.u. All spectroscopic information is concordant to the previous report [55].

4-Methyl-2-(phenanthren-3-yl)quinoline (**353**). Reaction time 3 min, cat. TFA. Pale yellow powder (70% yield, from *i*-PrOH), mp (166.5–168.3) °C. IR (cm^−1^) ʋ = 2980.1, 1599.6, 1148.8, 1129.6, 824.9, 751.1. ^1^H-NMR (DMSO-d_6_): δ 9.67 (1H, s), 9.13 (1H, d, *J* = 8.2 Hz), 8.59 (1H, d, *J* = 8.4 Hz), 8.53 (1H, s), 8.28 (1H, d, *J* = 8.4 Hz,), 8.22 (2H, dd, *J* = 14.2, 8.3 Hz), 8.07 (1H, d, *J* = 7.9 Hz), 8.00–7.94 (2H, m), 7.92 (1H, t, *J* = 7.7 Hz), 7.81 (1H, t, *J* = 7.7 Hz), 7.77–7.71 (2H, m), 2.91 (3H, s). ^13^C NMR (DMSO-d_6_): δ 133.21, 132.37, 131.30, 130.48, 130.32, 129.66, 129.18, 128.86, 127.74, 127.74, 127.66, 127.66, 127.55, 127.55, 127.48, 127.48, 126.88, 126.28, 125.01, 123.82, 122.98, 120.88, 19.21. MS (M^+^+H) 320.1 a.m.u., calc. 320.1 a.m.u., Δ = 0.0 a.m.u. All spectroscopic information is concordant to the previous report [54].

2-(*1H*-Indol-3-yl)-4-methylquinoline (**354**). Reaction time 2 min, cat. TFA. Pale yellow powder (60% yield, from i-PrOH), mp > 300 °C. IR (cm^−1^) ʋ = 3396.1, 3114.9, 3051.6, 1642.2, 1236.2, 768.1. ^1^H-NMR (DMSO-d_6_): δ 12.57 (1H, s), 8.74 (1H, s), 8.39 (1H, d, *J* = 8.5 Hz), 8.33 (1H, d, *J* = 7.0 Hz), 8.28 (1H, d, *J* = 8.5 Hz), 8.26 (1H, s), 8.02 (1H, t, *J* = 7.8 Hz), 7.79 (1H, t, *J* = 7.8 Hz), 7.63 (1H, d, *J* = 7.3 Hz), 7.36 (2H, q, *J* = 5.6, 3.9 Hz), 2.94 (3H, s). ^13^C-NMR (DMSO-d_6_): δ 137.74, 133.56, 133.09, 132.98, 127.91, 127.88, 125.95, 125.83, 125.77, 124.74, 123.89, 122.56, 122.52, 121.23, 120.68, 113.48, 19.71. MS (M^+^+H) 259.12297 a.m.u., calc. 259.12352 a.m.u., Δ = 0.00055 a.m.u. All spectroscopic information is concordant to the previous report [54].

9-Methyl-2,3-dihydro-1*H*-cyclopenta[*b*]quinoline (**355**). Reaction time 4 min, cat. TFA. Pale grey powder (76% yield, from cyclohexane), mp 212.2–212.8 °C. IR (cm^−1^) ʋ = 3065, 2957, 1613, 908, 751. ^1^H-NMR (DMSO-d_6_): δ 8.29 (1H, d, *J* = 8.3 Hz), 8.08 (1H, d, *J* = 8.3 Hz), 7.93 (1H, t, *J* = 7.3 Hz), 7.79 (1H, t, *J* = 7.3 Hz), 3.34 (2H, t, *J* = 7.7 Hz), 3.14 (2H, t, *J* = 7.5 Hz), 2.75 (3H, s), 2.26 (2H, p, *J* = 7.6 Hz).^13^C-NMR (DMSO-d_6_): δ 164.84, 137.03, 131.79, 128.05, 127.15, 125.39, 33.24, 29.57, 22.95, 16.25. MS (M^+^+H) 184.11208 a.m.u., calc. 184.11208 a.m.u., Δ = 0.00000 a.m.u. All spectroscopic information is concordant to the previous report [56].

9-Methyl-1,2,3,4-tetrahydroacridine (**356**). Reaction time 7 min, cat. TFA. Yellowish-white powder (60% yield, from cyclohexane), mp 193.7–194 °C. IR (cm^−1^) ʋ = 3068, 2935, 1614, 1581, 1350, 755. ^1^H-NMR (DMSO-d_6_): δ 8.38 (1H, d, *J* = 8.5 Hz), 8.08 (1H, d, *J* = 8.4 Hz), 7.96 (1H, t, *J* = 7.5 Hz), 7.80 (1H, t, *J* = 7.6 Hz), 3.22 (2H, d, *J* = 5.1 Hz), 2.96 (2H, d, *J* = 5.7 Hz), 2.75 (3H, s), 1.91 (4H, p, *J* = 3.3 Hz). ^13^C-NMR (DMSO-d_6_): δ 158.67, 158.46, 156.64, 132.59, 130.74, 128.29, 126.83, 125.47, 122.43, 30.43, 26.21, 22.00, 21.07, 15.26. MS (M^+^+H) 198.12773 a.m.u., calc. 198.12773 a.m.u., Δ = 0.00000 a.m.u. All spectroscopic information is concordant to the previous report [56].

11-Methyl-7,8,9,10-tetrahydro-*6H*-cyclohepta[*b*]quinoline (**357**). Reaction time 6 min, cat. TFA. Pale brown powder (85% yield, from AcOEt), mp 164–165 °C. IR (cm^−1^) ʋ = 2925, 1643, 1217, 744. ^1^H-NMR (DMSO-d_6_): δ 8.28 (1H, d, *J* = 8.4 Hz), 8.01 (1H, d, *J* = 8.3 Hz), 7.87 (1H, t, *J* = 7.6 Hz), 7.74 (1H, t, *J* = 7.6 Hz), 3.29–3.27 (2H, m), 3.10–3.08 (2H, m), 2.77 (3H, s), 1.88–1.84 (2H, m), 1.79–1.75 (2H, m), 1.70–1.66 (2H, m). ^13^C-NMR (DMSO-d_6_): δ 162.88, 146.87, 135.74, 139.8, 131.17, 127.94, 127.20, 125.62, 124.53, 36.69, 31.18, 28.82, 27.16, 26.33, 19.00. All spectroscopic information is concordant to the previous report [56].

10-Methyl-11*H*-indeno[1,2-*b*]quinoline (**358**). Reaction time 3 min, cat. HCl. White needles (60% yield, from EtOH), mp 267–268.8 °C. IR (cm^−1^) ʋ = 1923, 1607, 1462, 1386, 1157, 934, 753, 726. ^1^H-NMR (DMSO-d_6_): δ 8.53 (1H, d, *J* = 7.8 Hz), 8.38 (1H, d, *J* = 8.4 Hz), 8.35 (1H, d, *J* = 8.4 Hz), 8.00 (1H, t, *J* = 7.8 Hz), 7.86–7.81 (2H, m), 7.74 (1H, t, *J* = 7.8 Hz), 7.66 (1H, t, *J* = 7.5 Hz), 4.27 (2H, s), 2.92 (3H, s). ^13^C-NMR (DMSO-d_6_): δ 147.25, 135.14, 133.02, 132.00, 128.50, 127.89, 127.04, 127.02, 126.67, 125.49, 124.21, 124.16, 34.13, 16.19. MS 232.3 (M^+^+H) 232.1 a.m.u., calc. 232.1 a.m.u., Δ = 0.0 a.m.u. All spectroscopic information is concordant to the previous report [54].

10-Methyl-11*H*-[1,3]dioxolo[4′,5′:5,6]indeno [1,2-*b*]quinoline (**359**). Reaction time 3 min, cat. HCl. Yellow powder (77% yield, from EtOH), mp 279–281 °C. ^1^H-NMR (DMSO-d_6_): δ 8.23 (1H, s), 8.15–8.10 (1H, m), 7.89–7.65 (3H, m), 7.35 (1H, s), 6.21 (2H, s), 4.06 (2H, s), 2.79 (3H, s). ^13^C-NMR (DMSO-d_6_) δ 146.62, 143.23, 138.59, 132.50, 130.26, 127.72, 127.01, 125.82, 124.70, 122.13, 106.73, 102.62, 55.38, 33.68. IR (cm^−1^) ʋ = 3018, 2877, 1913, 1642, 1607, 1516, 1473, 1464, 1033, 977, 771. MS (M^+^-118) 157.0 a.m.u. (100% purity HPLC/MS). All spectroscopic information is concordant to the previous report [54].

10-Methyl-11*H*-indeno[1,2-*b*]quinolin-11-one (**360**). Reaction time 1 min, cat. HCl. Pale yellow needles (55% yield, from EtOH), mp 229–230 °C. IR (cm^−1^) ʋ = 1700.0, 1578.8, 1220.6, 859.9, 770.2. ^1^H-NMR (DMSO-d_6_): δ 8.22 (1H, dd, *J* = 8.3, 1.4 Hz), 8.04 (1H, dd, *J* = 8.2, 1.2 Hz), 8.01 (1H, dt, *J* = 7.4, 1.0 Hz), 7.84 (1H, ddd, *J* = 8.3, 6.9, 1.4 Hz), 7.80–7.75 (2H, m), 7.65 (1H, ddd, *J* = 8.4, 6.9, 1.4 Hz), 7.63–7.60 (1H, m), 2.98 (3H, s). ^13^C-NMR (DMSO-d_6_): δ 192.34, 161.57, 149.33, 147.12, 142.87, 137.49, 136.28, 132.54, 132.43, 130.24, 128.49, 127.76, 126.90, 124.06, 123.44, 121.92, 12.67. MS (M^+^+H) 246.09134 a.m.u., calc. 246.09134 a.m.u., Δ = 0.00000 a.m.u. All spectroscopic information is concordant to the previous report [57].

### 3.3. Biology

#### 3.3.1. Maintenance of In Vitro Culture

The *P. falciparum* 3D7 strain was kept in culture in a humidified incubator at 37 °C in RPMI-1640 medium with 25 mM NaHCO_3_, 25 mM HEPES (pH 7.4), 11 mM D-glucose, 3.67 mM hypoxanthine, and 50 mg/mL gentamicin, supplemented with 0.5% AlbuMAX II. The culture medium was changed daily, and the parasitaemia was maintained below 5% with 2.5% haematocrit in human O+ erythrocytes [58].

#### 3.3.2. SYBR Green I Inhibition Assay for the Asexual Stages of *P. falciparum*

The parasites were synchronized through sterile 5% (*m*/*v*) D-sorbitol treatment over 10 min at 37 °C for the enrichment of ring-stage parasites [59]. The cultures were pelleted by centrifugation at 600× *g* for 5 min. The parasitaemia was determined by microscope analysis of thin blood smears stained with Giemsa 10% after methanol fixation. The initial parasitaemia was calculated from 1000 red blood cells (RBCs), and cultures were diluted to 0.5% parasitaemia and 2% haematocrit by adding the appropriate volumes of erythrocytes and medium. Parasite aliquots of 180 µL were distributed into 96-well plates previously prepared with 20 µL aliquots of a 10-fold concentrated compound. The concentration of the quinoline derivatives ranged from 100 µM to 1.5 µM (two-fold serial dilutions were carried out). Chloroquine was used as positive control and tested in a concentration ranging from 100 nM to 1.5 nM. The tested compounds were diluted to a stock concentration of 20 mM in 100% DMSO before the experiments and maintained at −20 °C. The DMSO concentration in the assay was maintained below 0.5% (*v*/*v*). Negative and positive control wells corresponding to non-parasitized erythrocytes and parasite cultures in the absence of compounds were set in parallel. The DMSO concentration was maintained below 0.5% (*v*/*v*). The plates were incubated for 72 h at 37 °C in a humidified incubator with a gas mixture of 90% N_2_, 5% O_2_, and 5% CO_2_. After incubation, the culture medium was removed, and the cells were resuspended in 100 µL PBS buffer (116 mM NaCl, 10 mM Na_2_HPO_4_, 3 mM KH_2_PO_4_) and lysed with 100 µL lysis buffer (20 mM Tris base, 5 mM EDTA, 0.0008% (*v*/*v*) Triton X-100, and 0.008% (*m*/*v*) saponin, pH 8.0) containing 0.002% (*v*/*v*) SYBR Green I. Plates were incubated at room temperature for 30 min, and the fluorescence corresponding to the parasitic density was determined using a SpectraMAX Gemini EM plate reader (Molecular Devices Corp., Sunnyvale, CA, USA) (excitation at 485 nm, emission at 535 nm). The inhibitory activity of each compound was assessed in duplicate, and the results were compared with the control cultures grown in complete medium with no inhibitors. The half maximal inhibitory concentration (IC_50_) was determined by non-linear regression analysis of the resulting concentration–response curve using the software Origin 2016 (OriginLab Corporation, Northampton, MA, USA). The reported IC_50_ values represent the average and standard deviation of two independent experiments.

#### 3.3.3. Hepatocarcinoma Cell Cultures and Cytotoxicity Evaluation

Hepatocarcinoma cells (HepG2) were cultivated in RPMI medium supplemented with 10% (*v*/*v*) fetal bovine serum and 0.2% (*v*/*v*) penicillin/streptomycin. The antibiotics were added to the medium to eliminate the potential interference of microbial contamination. Cells were cultivated at 37 °C and 5% CO_2_; the supplemented medium was changed every two days.

For the experimental procedures, cells were trypsinized and transferred to a 96-well plate at 10,000 cells per well and incubated at 37 °C for 24 h for cell adhesion. Then, serial dilutions of the inhibitor candidate were added to the plate. Cells without any compounds were used as positive growth controls. The plate was incubated at 37 °C and 5% CO_2_ for 72 h. After incubation, the highest compound concentration to be considered (highest concentration without precipitation) was observed by microscopy. The assessed concentrations of the 4-methylquinoline derivative ranged from 100 µM to 1.5 µM (two-fold serial dilutions were carried out). Cytotoxicity was evaluated by a colorimetric assay based on metabolic cell activity in the presence of 3-(4,5-dimethylthiazol-2-yl)-2,5-diphenyltetrazolium bromide (MTT) [60]. Briefly, mitochondrial enzymes can convert the MTT dye into the purple insoluble compound formazan. To each well, 20 µL MTT at 5 mg/mL was added, followed by incubation at 37 °C for 3 to 5 h. After incubation, the supernatant was removed, and formazan crystals were solubilized in 100 µL DMSO. The absorbance, which is proportional to the number of viable cells, was determined using a SpectraMAXPlus 384 plate reader (Molecular Devices Corp., Sunnyvale, CA) (λ = 570 nm) [61]. The half maximal inhibitory concentration (IC_50_) was determined by non-linear regression analysis of the resulting concentration-response curve using the software Origin 2016 (OriginLab Corporation).

## 4. Conclusions

In the current research, the QSAR models were developed based on comparative molecular field analysis (CoMFA), comparative molecular similarity index analysis (CoMSIA), and 2D-QSAR analysis using an extensive database of three hundred forty-nine quinoline derivatives with inhibition activity on *P. falciparum* 3D7. The models were validated using internal and external statistical validation, complying with all the parameters used. In addition, the models were experimentally tested to check their predictive power. For this purpose, we synthesized ten quinoline derivatives, and their activity was assessed on *P. falciparum* 3D7; we found that that the models CoMSIA-SEHDA and 2D-QSAR showed the best predictability (ΔpIC_50_ < 1.0). This phenomenon could be attributed to the balanced contribution between all the fields assessed (steric, electrostatic, hydrophobic, hydrogen-bonding donor, and acceptor).

In addition, the physicochemical and pharmacokinetic properties of three synthesized compounds and chloroquine were assessed, evidencing similar absorption and distribution properties. In this sense, the biocompatibility of these synthesized compounds was studied using a hepatic cell model, checking for a low cytotoxic effect (IC_50_ > 100 µM).

These results suggest that the CoMSIA-SEHDA and 2D-QSAR models could be used for the rational design of new quinoline derivatives with anti-malaria effect against the *P. falciparum* 3D7 strain. Finally, in the broadest sense, the present research shows that the design and synthesis of new chloroquine-type derivatives is still possible, but with an improved selectivity profile, and with the structural novelty of not having a basic nitrogen atom in the chemical structure.

## Figures and Tables

**Table 1:** Summary of external validation parameters for CoMFA and CoMSIA.

Condition	Parameters	Threshold Value	CoMFA-SE	CoMSIA-SEHDA
**1**	$q^{2}$	>0.5	0.765	0.796
**2**	$r_{test}^{2}$	>0.6	0.878	0.876
**3a**	$r_{0}^{2}$	$\mathrm{Close}\;\mathrm{to}\;\mathrm{value}\;\mathrm{of}\;r_{test}^{2}$	0.954	0.954
**3b**	${r\prime }_{0}^{2}$	$\mathrm{Close}\;\mathrm{to}\;\mathrm{value}\;\mathrm{of}\;r_{test}^{2}$	0.935	0.935
**4a**	k	0.85 < k < 1.15	0.990	0.990
**4b**	k′	$0.85\;<\;k^{\prime }$ < 1.15	1.005	1.005
**5a**	${(r}^{2}-r_{0}^{2})/r^{2}$	<0.1	0.089	0.089
**5b**	${(r}^{2}-{r\prime }_{0}^{2})/r^{2}$	<0.1	0.067	0.067
**6**	$\left|r_{0}^{2}-{r\prime }_{0}^{2}\right|$	<0.3	0.019	0.019
**7**	$r_{m}^{2}$	>0.5	0.720	0.720

**Table 2:** Experimental and predicted activity (CoMFA-SE, CoMSIA-SEHDA, and 2D-QSAR) for compounds **350**–**360** against *P. falciparum*.

Compound	IC_50_ (µM)	pIC_50_						
		Experimental	CoMFA		CoMSIA		2D-QSAR	
			Prediction	Residual	Prediction	Residual	Prediction	Residual
**350**	>40.0	−	−	−	−	−		
**351**	35.51	4.45	6.37	−1.92	5.317	−0.867	5.124	−0.67
**352**	15.14	4.82	6.235	−1.415	5.481	−0.661	5.074	−0.25
**353**	1.51	5.821	6.02	−0.199	5.432	0.389	5.067	0.75
**354**	5.24	5.281	5.91	−0.629	5.398	−0.117	5.381	−0.10
**355**	22.60	4.646	6.093	−1.447	5.391	−0.745	5.023	−0.38
**356**	30.36	4.518	6.16	−1.642	5.428	−0.91	4.989	−0.47
**357**	22.48	4.648	6.127	−1.479	5.414	−0.766	4.955	−0.31
**358**	23.00	4.638	5.91	−1.272	5.425	−0.787	5.086	−0.45
**359**	22.11	4.655	6.08	−1.425	5.384	−0.729	5.130	−0.47
**360**	27.04	4.568	5.908	−1.34	5.359	−0.791	5.165	−0.60
**CQ**	0.285	6.545	7.86	−1.315	7.49	−0.945	5.667	0.88
**RMSEP**			1.3576		0.7381		0.5315	
**MAE**			1.2803		0.7006		0.4849	

CQ = chloroquine; RMSEP = root mean squared error of prediction; MAE = mean absolute error.

**Table 3:** Summary of the calculated physicochemical and pharmacokinetic properties of the selected compounds from the experimental set.

Comp.	MW ^a^	CLogP ^b^	nOH ^c^	nOHNH ^d^	TPSA ^e^	NV ^f^	HIE(p) ^g^	Caco-2(p) ^h^	BBB(p) ^i^
**353**	319.40	3.53	1	0	12.89	1	+(1.00)	+(0.72)	+(0.98)
**354**	258.32	2.50	1	1	24.68	0	+(1.00)	+(0.78)	+(0.97)
**356**	197.28	2.61	1	0	12.89	0	+(1.00)	+(0.73)	+(0.98)
**CQ ^j^**	319.87	3.95	8	2	28.16	0	+(0.99)	+(0.66)	+(1.00)

MW ^a^: molecular weight; CLogP ^b^: calculated partition coefficient; nOH ^c^: number of hydrogen donor groups; NOHNH ^d^: number of hydrogen acceptor groups; TPSA ^e^: topological polar surface area; NV ^f^: number of violations of Lipinski’s rule; HIE(p) ^g^: human intestinal epithelium; Caco-2(p) ^h^: cell permeability in Caco-2; BBB(p) ^i^: blood–brain barrier; CQ ^j^: chloroquine.

## Data Availability

Data is contained within the article or Supplementary Materials.

## Supplementary Materials

The following supporting information can be downloaded from: https://www.mdpi.com/article/10.3390/ph17070889/s1, Section S1. Details of the theoretical models; Figure S1. The superimposed structures of all compounds used in the CoMFA/CoMSIA models using the quinoline core; Figure S2: Histogram of frequency distribution data; Table S1: Chemical structure of dataset used to develop the 3D-QSAR models; Table S2: Field combination of CoMFA and CoMSIA models of *P. falciparum* inhibitors; Table S3: Experimental and predicted pIC_50_ and residual values for analyzed compounds according to CoMFA and CoMSIA.; Table S4: *Y*-randomization test for CoMFA and CoMSIA models; Table S5. The SMILES codes for the compounds; Table S6. Experimental and predicted activities for the 2D-QSAR model; Figure S3: CoMSIA donor (A,B) and acceptor (C,D) contour maps around compounds 353 (left) and 356 (right), the most active and least active of the designed compounds series, respectively. Figure S4: CoMSIA steric (A,B), electrostatic (C,D), hydrophobic (E,F), donor (G,H), and acceptor (I,J) contour maps around compounds 354 (left) and 351 (right), the most active and least active of the series, respectively; References. References [62,63,64,65,66] are cited in the supplementary materials.
