# Supplementary material for: 2D/3D-QSAR Model Development Based on a Quinoline Pharmacophoric Core for the Inhibition of Plasmodium falciparum: An In Silico Approach with Experimental Validation
Source: Pharmaceuticals (Basel). 2024 Jul 4;17(7):889. doi: 10.3390/ph17070889 (PMC11279914; doi:10.3390/ph17070889)
Supplement: Supplementary file 1 [file pharmaceuticals-17-00889-s001.zip › pharmaceuticals-3042165-supplementary.pdf]

SUPPLEMENTARY MATERIALS

2D/3D-QSAR models development based in quinoline pharmacophoric core to inhibition of *Plasmodium falciparum*: *In silico* approach with experimental validation

Marcos Lorca[1], Gisela C. Muscia[2], Susana Pérez-Benavente[3], José M. Bautista[3], Alison Acosta[4], Cesar González[5], Gianfranco Sabadini[1], Jaime Mella[1,6,*], Silvia E. Asís[2,*], Marco Mellado[7,*].

[1] Instituto de Química y Bioquímica, Facultad de Ciencias, Universidad de Valparaíso, Av. Gran Bretaña 1111, Valparaíso 2360102, Chile; marcos.lorca.c@gmail.com (M.L.); jaime.mella@uv.cl (J.M.)

[2] Universidad de Buenos Aires. Facultad de Farmacia y Bioquímica, Departamento de Ciencias Químicas, Junín 956. C1113AAB Ciudad Autónoma de Buenos Aires, Argentina; gmuscia@ffyb.uba.ar (G.C.M.); elizabet@ffyb.uba.ar (S.E.A.)

[3] Departamento de Bioquímica y Biología Molecular, Universidad Complutense de Madrid, Facultad de Veterinaria, E28040 Madrid, Spain; susipz@ucm.es (S.P.B.); jmbau@ucm.es (J.M.B.)

[4] Universidad Andres Bello, Facultad de Ciencias Exactas, Departamento de Ciencias Químicas, Quillota 980, Viña del Mar, Chile; al.acostaq@uandresbello.edu (A.A.)

[5] Departamento de Química, Universidad Técnica Federico Santa María, Av. España 1680, 2390123 Valparaíso, Chile; cesar.gonzalez@usm.cl (C.G.)

[6] Centro de Investigación Farmacopea Chilena (CIFAR), Universidad de Valparaíso, Santa Marta 183, 2360134 Valparaíso, Chile. jaime.mella@uv.cl (J.M.)

[7] Instituto de Investigación y Postgrado, Facultad de Medicina y Ciencias de la Salud, Universidad Central de Chile, 8330507 Santiago, Chile; marco.mellado@ucentral.cl (M.M.)

* Correspondence: jaime.mella@uv.cl; Tel.: +56 32 250 8067 (J.M.); elizabet@ffyb.uba.ar; Tel.: +54 11 5287 4905 (S.E.A.); marco.mellado@ucentral.cl; Tel.: 56 2 2582 6567 (M.M.)

Abstract: Malaria is an infectious disease caused by *Plasmodium* spp. parasites, with widespread drug resistance to most antimalarials drugs. To this respect, a great challenge is now the discovery of novel antimalarial compounds with favorable properties through bioinformatics tools. Here we report the development of two 3D-QSAR models based on Comparative Molecular Field Analysis-CoMFA, and Comparative Molecular Similarity Index Analysis-CoMSIA, and a 2D-QSAR model, using a database of 349 compounds with activity against *P. falciparum* 3D7 strain. The models were validated internally and externally, complying with all metrics (q^2, r^2_{pred}, etc.). The models were experimentally tested through the synthesis of ten quinoline derivatives. The molecules were tested in *in-vivo* inhibitory activity against *P. falciparum* 3D7. The highest prediction was obtained for the models CoMSIA and 2D-QSAR. To complement this study, the physicochemical and pharmacokinetic properties of three selected quinoline derivatives were examined, finding similar properties to chloroquine. Finally, these three compounds were assessed for cytotoxicity on human HepG2 cells showing low toxicity level (IC_{50}> 100 μM). These results suggest that the CoMSIA and 2D-QSAR models are ready to use tools for the rational design of new quinoline derivatives with antimalarial activity against *P. falciparum* with a safe toxicological profile.

Table of content

1. Details of the theoretical model ……………………………….. **page 3**
1.1. Molecular Alignment ……………………………………………………………………………………... **page 3**
1.2. CoMFA and CoMSIA Field Calculation ………………………………………………………………… **page 3**
Figure S1. The superimposed structures of all compounds used in the CoMFA/CoMSIA models .. ………... **page 3**
Figure S2: Histogram of frequency distribution data ………………………………………………..…… **page 5**
Table S1: Chemical structure of dataset used to develop the 3D-QSAR models ………………………………… **page 6**
Table S2: Field combination of CoMFA and CoMSIA models of *P. falciparum* inhibitors ……..………………..…. **page 27**
Table S3: Experimental and predicted pIC_{50} and residual values for analyzed compounds according to CoMFA and CoMSIA ……………………………………………………………………………………………………... **page 28**
Table S4: *Y*-randomization test for CoMFA and CoMSIA models ……………….…………………………….. **page 35**
Table S5. The SMILES codes for the compounds……………………………………………………………… **page 38**
Table S6. Experimental and predicted activities for the 2D-QSAR model……………………………………….. **page 49**
Figure S3: CoMSIA donor (A,B) and acceptor (C,D) contour maps around compounds 353 (left) and 356 (right), the most active and least active of the designed compounds series respectively ……………………………………... **page 36**
Figure S4: CoMSIA steric (A,B), electrostatic (C,D), hydrophobic (E,F), donor (G,H) and acceptor (I,J) contour maps around com-pounds 354 (left) and 351 (right), the most active and least active of the series respectively …….. **page 37**
References …….. **page 50**

1. Details of the theoretical model

1.1. Molecular Alignment

3D-QSAR studies were performed with Sybyl X-1.2 software installed in a Windows 10 environment on a PC with an Intel core i7 CPU, according our previous reports.[1–4] Molecular alignment was carried out using the distill protocol implemented in Sybyl. The common quinoline core was used as a template for molecular overlay and alignment, as shown below

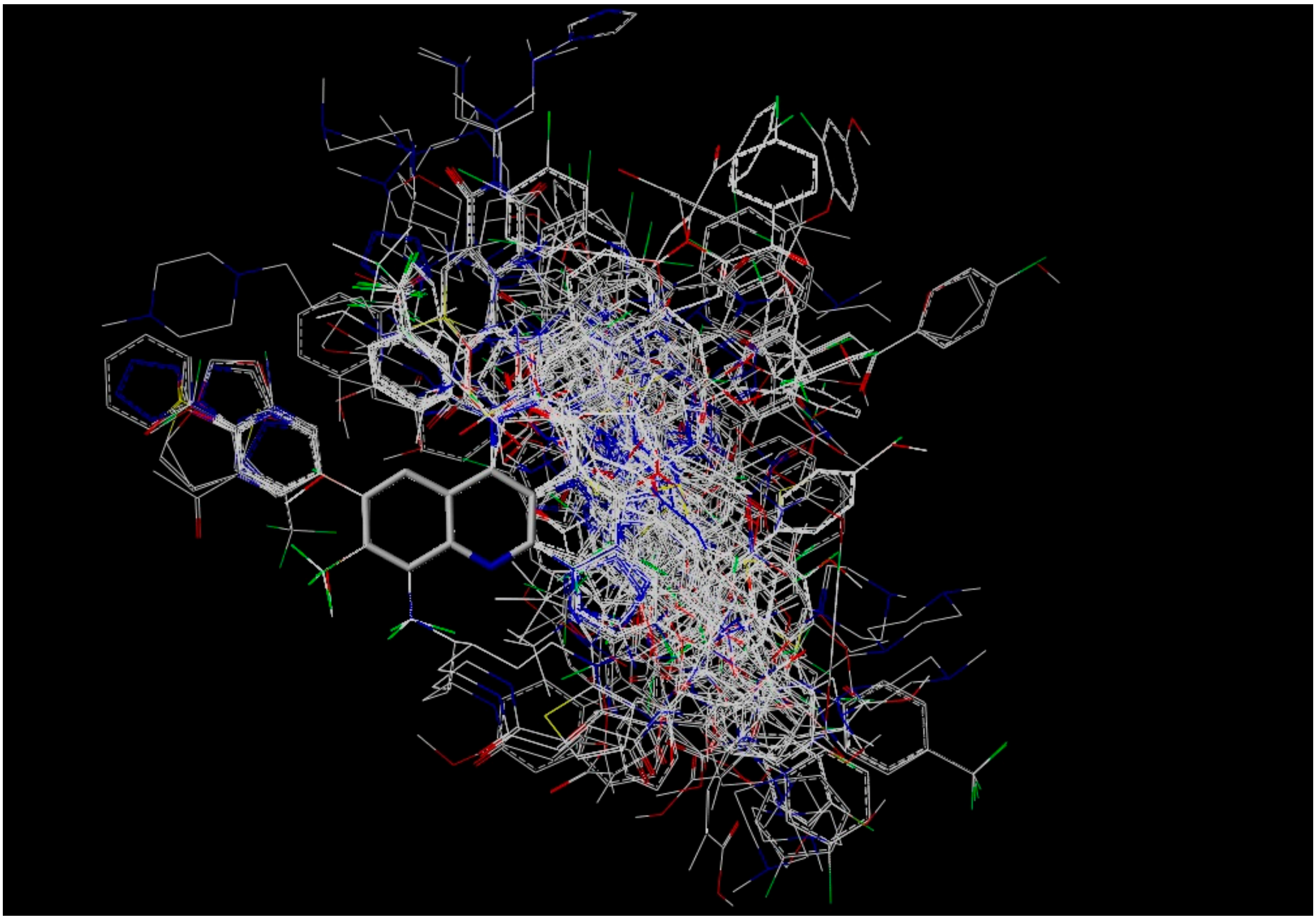

Figure S1. The superimposed structures of all compounds used in the CoMFA/CoMSIA models. The common quinoline nucleus used as the template for the alignment is highlighted in thick lines.

1.2. CoMFA and CoMSIA Field Calculation

To derive the CoMFA and CoMSIA descriptor fields, the aligned training set molecules were placed in a three-dimensional cubic lattice with a grid spacing of 2Å in the x, y and z directions such that the entire set was included on it. The CoMFA steric and electrostatic field energies were calculated using a sp^3 carbon probe atom with a van der Waals radius of 1.52Å and a charge of +1.0. Cut-off values for both steric and electrostatic fields were set to 30.0 kcal/mol. For CoMSIA analysis, the standard settings (probe with charge +1.0, radius 1Å, hydrophobicity +1.0, H-bond donating +1.0, and H-bond accepting +1.0) were used to calculate five different fields: steric, electrostatic, hydrophobic, donor and acceptor.[5] Gaussian-type distance dependence was used to measure the relative attenuation of the field position of each atom in the lattice and led to a much smoother sampling of the fields around the molecules when compared to CoMFA. The default value of 0.3 was set for attenuation factor α.

1.3. Internal Validation

PLS analysis was used to construct a linear correlation between the CoMSIA descriptors (independent variables) and the activity values (dependent variables) [46]. To select the best model, the cross-validation analysis was performed by

using the LOO method (and SAMPLS), which generates the square of the cross-validation coefficient (q^2) and the optimum number of components (N). The non-cross-validation was performed with a column filter value of 2.0 in order to speed up the analysis and reduce the noise. The q^2, which is a measure of the internal quality of the models, was obtained according to the following Equation (1):

$$q^2 = 1 - \frac{\Sigma(y_i - y_{pred})^2}{\Sigma(y_i - y_{ave})^2} \quad \text{(Eq. 1)}$$

Where y_i is the observed activity of the training set, y_{pred} is the predicted activity of the training set, and y_{ave} is the average of the activity of the training set.

1.4. External Validation

The predictive power of the models was assessed by calculation of the predictive r2 (r2pred). r2pred measures the predictive performance of a PLS model and is defined according to Equation (2):

$$r^2_{pred} = \frac{SD - PRESS}{SD} \quad \text{(Eq. 2)}$$

where SD is the sum of the squared deviations between the biological activities of the test set compounds and mean activity of the training set compounds, and PRESS is the sum of squared deviations between observed and predicted activities of the test set compounds.

In addition, the models were subjected to external validation criteria which considers a predictive QSAR model, if the following conditions are satisfied:

$$q^2 > 0.5 \quad \text{(Eq. 3)}$$

$$r^2_{test} > 0.6 \quad \text{(Eq. 4)}$$

$$\frac{(r^2_{test} - r^2_0)}{r^2_{test}} < 0.1 \text{ or } \frac{(r^2_{test} - r'^2_0)}{r^2_{test}} < 0.1 \quad \text{(Eq. 5)}$$

$$0.85 \leq k \leq 1.15 \text{ or } 0.85 \leq k' \leq 1.15 \text{ (Eq. 7)}$$

$$|r^2_0 - r'^2_0| < 0.3 \quad \text{(Eq. 8)}$$

$$r^2_m = r^2_{test}\,(1 - \left|\sqrt{r^2_{test} - r^2_0}\right|) \quad \text{(Eq. 9)}$$

Where r^2_{test} and r^2_0 are the squared correlation coefficients between the observed and predicted activities of the test set with and without the (0,0) intercept, respectively. For a significant external model validation, the value of r^2_m should be greater than 0.5.

1.5. Dataset Selection and Inhibition Activity

3D-QSAR studies were performed on a set of 349 compounds with inhibition activity on *Plasmodium falciparum* 3D7, measured as half-inhibition concentration (IC_{50}), and reported previously.[6–27] The structures of all the compounds are represented in **Table S1**. The derivatives displayed IC_{50} activities, as represented in **Table S2**. The IC_{50} values were converted to pIC_{50} (-log IC_{50}). The compounds were randomly divided into training (270 compounds, 77%) and test (79 compounds, 23%) sets. The distribution of pIC_{50} values for the whole set, the training set and the test set, is shown in **Figure S2**.

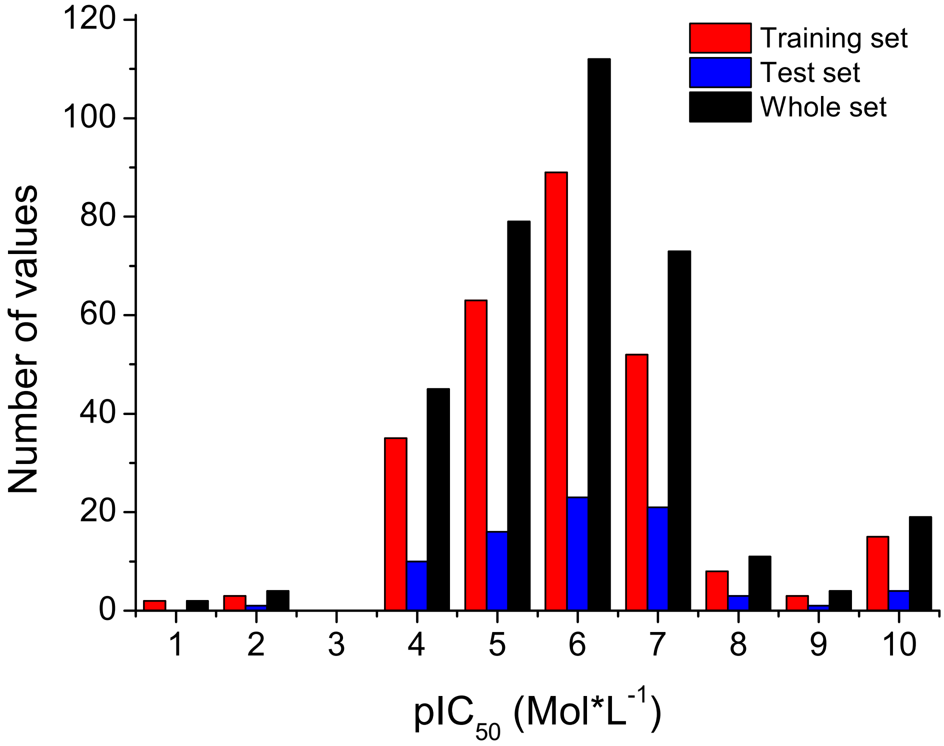

Figure S2: Histogram of frequency distribution data.

Table S1: Chemical structure of dataset used to develop the 3D-QSAR models.

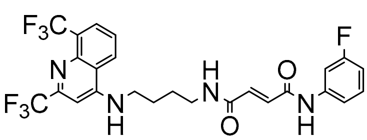 **001** IC_{50}= 2.9 μM; pIC_{50}= 5.538 M; [6]	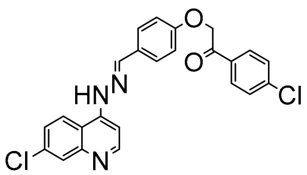 **002** IC_{50}= 0.0367 μM; pIC_{50}= 7.435 M; [9]	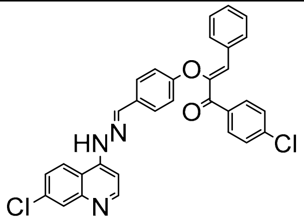 **003** IC_{50}= 0.07877 μM; pIC_{50}= 7.104 M; [9]
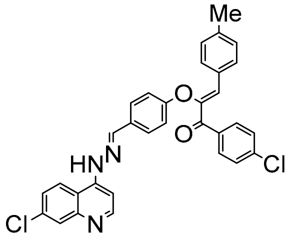 **004** IC_{50}= 0.11168 μM; pIC_{50}= 6.952 M; [9]	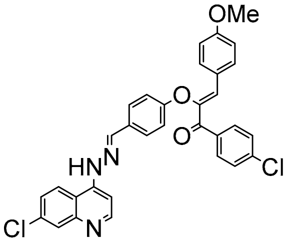 **005** IC_{50}= 0.04809 μM; pIC_{50}= 7.318 M; [9]	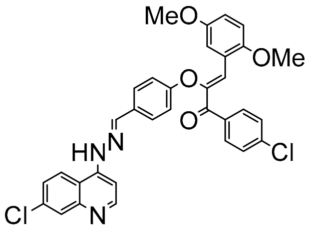 **006** IC_{50}= 0.25041 μM; pIC_{50}= 6.601 M; [9]
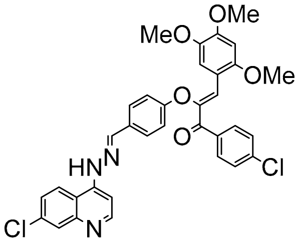 **007** IC_{50}= 0.09049 μM; pIC_{50}= 7.043 M; [9]	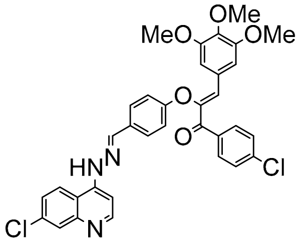 **008** IC_{50}= 0.09531 μM; pIC_{50}= 7.021 M; [9]	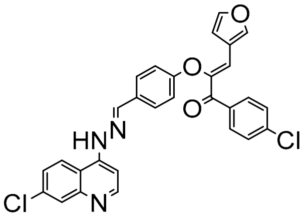 **009** IC_{50}= 0.05730 μM; pIC_{50}= 7.242 M; [9]
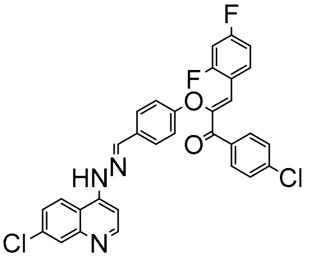 **010** IC_{50}= 0.13881 μM; pIC_{50}= 6.858 M; [9]	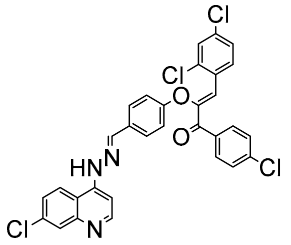 **011** IC_{50}= 0.29542 μM; pIC_{50}= 6.530 M; [9]	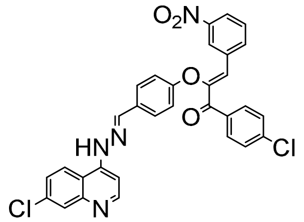 **012** IC_{50}= 0.12812 μM; pIC_{50}= 6.892 M; [9]

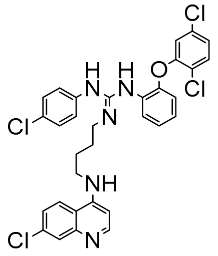<br>**013**<br>IC$_{50}$= 0.6535 μM;<br>pIC$_{50}$= 6.185 M;<br>[9]	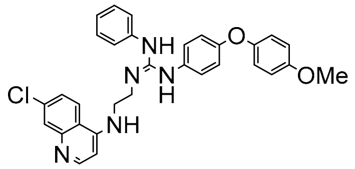<br>**014**<br>IC$_{50}$= 0.042 μM;<br>pIC$_{50}$= 7.377 M;<br>[10]	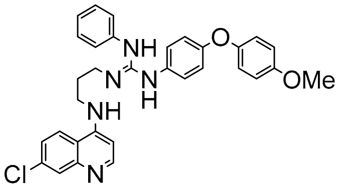<br>**015**<br>IC$_{50}$= 0.1203 μM;<br>pIC$_{50}$= 6.920 M;<br>[10]
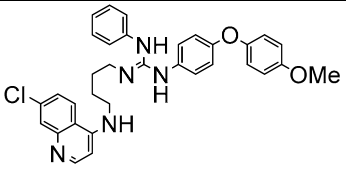<br>**016**<br>IC$_{50}$= 0.109 μM;<br>pIC$_{50}$= 6.963 M;<br>[10]	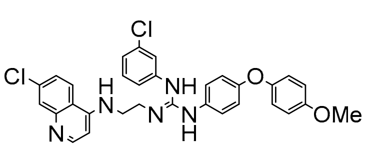<br>**017**<br>IC$_{50}$= 0.04342 μM;<br>pIC$_{50}$= 7.362 M;<br>[10]	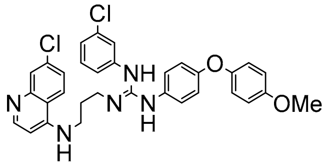<br>**018**<br>IC$_{50}$= 0.36918 μM;<br>pIC$_{50}$= 6.433 M;<br>[10]
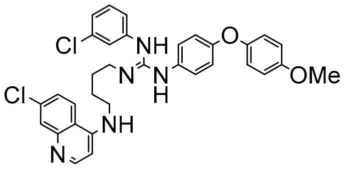<br>**019**<br>IC$_{50}$= 0.03756 μM;<br>pIC$_{50}$= 7.425 M;<br>[10]	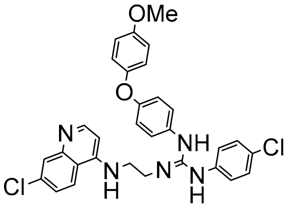<br>**020**<br>IC$_{50}$= 0.10324 μM;<br>pIC$_{50}$= 6.986 M;<br>[10]	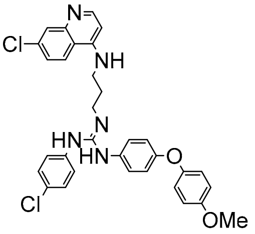<br>**021**<br>IC$_{50}$= 0.24082 μM;<br>pIC$_{50}$= 6.618 M;<br>[10]
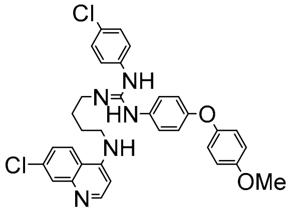<br>**022**<br>IC$_{50}$= 0.12388 μM;<br>pIC$_{50}$= 6.907 M;<br>[10]	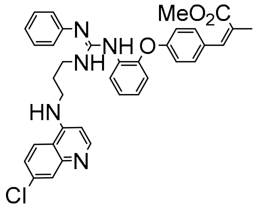<br>**023**<br>IC$_{50}$= 0.1745 μM;<br>pIC$_{50}$= 6.758 M;<br>[10]	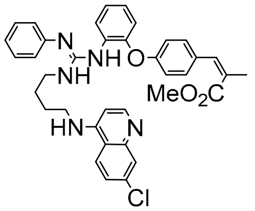<br>**024**<br>IC$_{50}$= 0.287 μM;<br>pIC$_{50}$= 6.542 M;<br>[10]
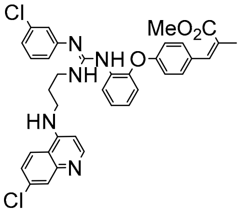<br>**025**<br>IC$_{50}$= 0.02034 μM;<br>pIC$_{50}$= 7.692 M;<br>[10]	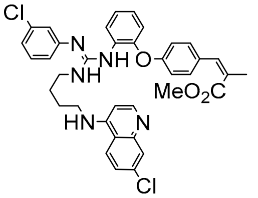<br>**026**<br>IC$_{50}$= 0.03123 μM;<br>pIC$_{50}$= 7.505 M;<br>[10]	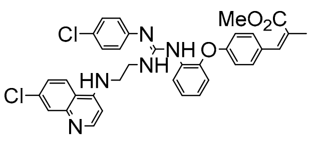<br>**027**<br>IC$_{50}$= 0.05151 μM;<br>pIC$_{50}$= 7.288 M;<br>[10]

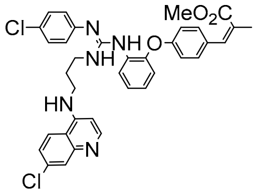<br>**028**<br>IC$_{50}$= 0.07448 μM;<br>pIC$_{50}$= 7.128 M;<br>[10]	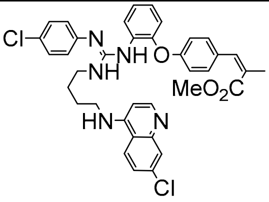<br>**029**<br>IC$_{50}$= 0.09679 μM;<br>pIC$_{50}$= 7.014 M;<br>[10]	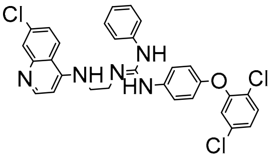<br>**030**<br>IC$_{50}$= 0.03945 μM;<br>pIC$_{50}$= 7.404 M;<br>[10]
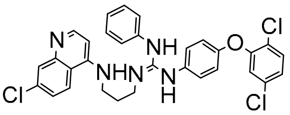<br>**031**<br>IC$_{50}$= 0.02312 μM;<br>pIC$_{50}$= 7.636 M;<br>[10]	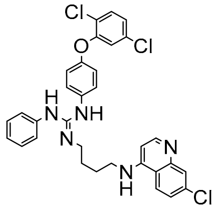<br>**032**<br>IC$_{50}$= 0.3074 μM;<br>pIC$_{50}$= 6.512 M;<br>[10]	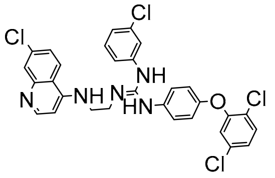<br>**033**<br>IC$_{50}$= 0.06275 μM;<br>pIC$_{50}$= 7.202 M;<br>[10]
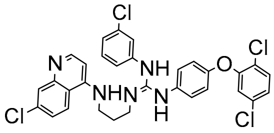<br>**034**<br>IC$_{50}$= 0.07624 μM;<br>pIC$_{50}$= 7.118 M;<br>[10]	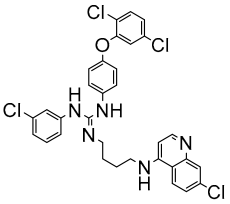<br>**035**<br>IC$_{50}$= 0.126 μM;<br>pIC$_{50}$= 6.900 M;<br>[10]	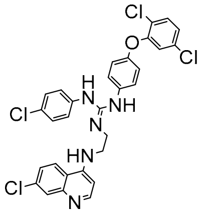<br>**036**<br>IC$_{50}$= 0.04397 μM;<br>pIC$_{50}$= 7.357 M;<br>[10]
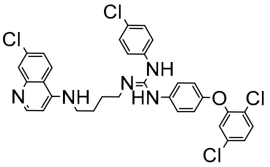<br>**037**<br>IC$_{50}$= 0.02902 μM;<br>pIC$_{50}$= 7.537 M;<br>[10]	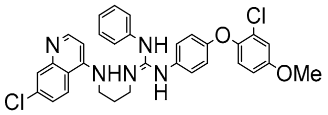<br>**038**<br>IC$_{50}$= 0.03663 μM;<br>pIC$_{50}$= 7.436 M;<br>[10]	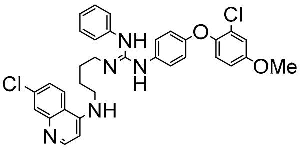<br>**039**<br>IC$_{50}$= 0.08601 μM;<br>pIC$_{50}$= 7.066 M;<br>[10]
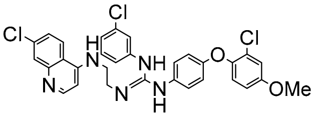<br>**040**<br>IC$_{50}$= 0.08873 μM;<br>pIC$_{50}$= 7.052 M;<br>[10]	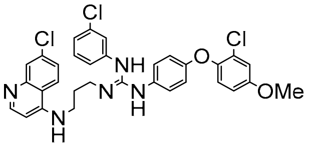<br>**041**<br>IC$_{50}$= 0.0496 μM;<br>pIC$_{50}$= 7.305 M;<br>[10]	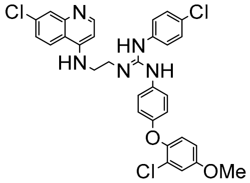<br>**042**<br>IC$_{50}$= 0.06903 μM;<br>pIC$_{50}$= 7.161 M;<br>[10]

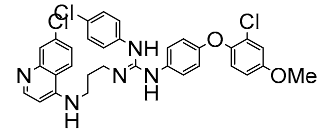<br>**043**<br>IC50= 0.2041 μM;<br>pIC50= 6.690 M;<br>[10]	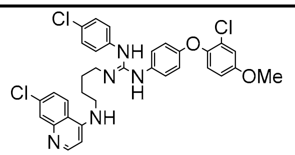<br>**044**<br>IC50= 0.0337 μM;<br>pIC50= 7.472 M;<br>[10]	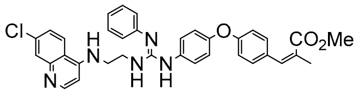<br>**045**<br>IC50= 0.07035 μM;<br>pIC50= 7.153 M;<br>[10]
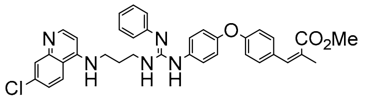<br>**046**<br>IC50= 0.07818 μM;<br>pIC50= 7.107 M;<br>[10]	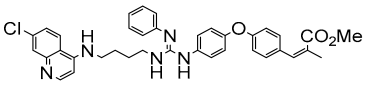<br>**047**<br>IC50= 0.09442 μM;<br>pIC50= 7.025 M;<br>[10]	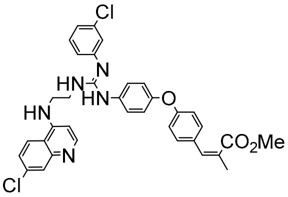<br>**048**<br>IC50= 0.04886 μM;<br>pIC50= 7.311 M;<br>[10]
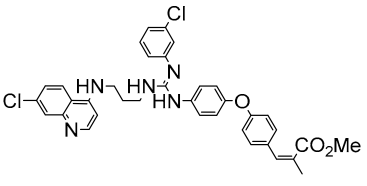<br>**049**<br>IC50= 0.07818 μM;<br>pIC50= 7.107 M;<br>[10]	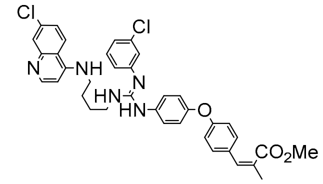<br>**050**<br>IC50= 0.05193 μM;<br>pIC50= 7.285 M;<br>[10]	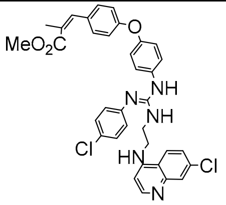<br>**051**<br>IC50= 0.04309 μM;<br>pIC50= 7.366 M;<br>[10]
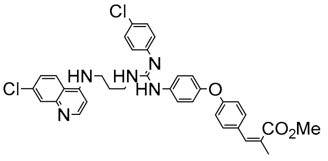<br>**052**<br>IC50= 0.03851 μM;<br>pIC50= 7.414 M;<br>[10]	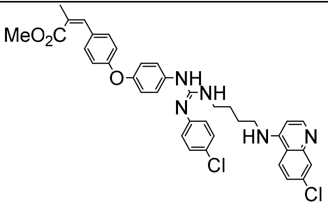<br>**053**<br>IC50= 0.05637 μM;<br>pIC50= 7.249 M;<br>[10]	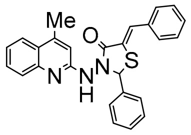<br>**054**<br>IC50= 1.012 μM;<br>pIC50= 6.000 M;<br>[11]
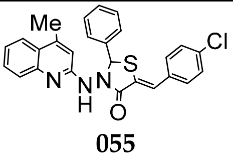<br>**055**<br>IC50= 0.731 μM;<br>pIC50= 6.136 M;<br>[11]	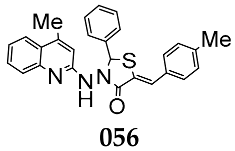<br>**056**<br>IC50= 1.212 μM;<br>pIC50= 6.000 M;<br>[11]	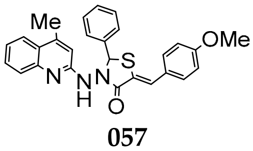<br>**057**<br>IC50= 1.230 μM;<br>pIC50= 6.000 M;<br>[11]

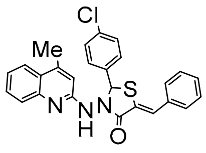<br>**058**<br>IC50= 0.734 μM;<br>pIC50= 6.134 M;<br>[11]	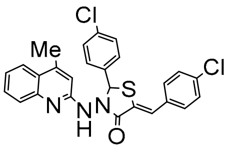<br>**059**<br>IC50= 0.783 μM;<br>pIC50= 6.106 M;<br>[11]	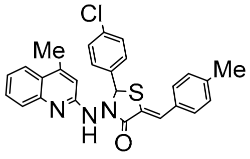<br>**060**<br>IC50= 0.423 μM;<br>pIC50= 6.374 M;<br>[11]
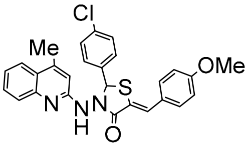<br>**061**<br>IC50= 0.791 μM;<br>pIC50= 6.102 M;<br>[11]	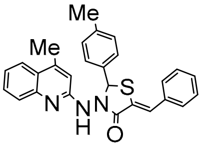<br>**062**<br>IC50= 1.501 μM;<br>pIC50= 5.699 M;<br>[11]	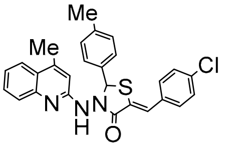<br>**063**<br>IC50= 0.562 μM;<br>pIC50= 6.250 M;<br>[11]
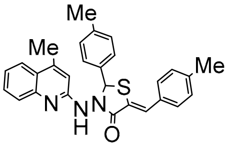<br>**064**<br>IC50= 1.732 μM;<br>pIC50= 5.699 M;<br>[11]	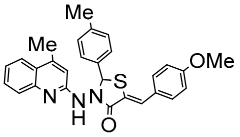<br>**065**<br>IC50= 1.621 μM;<br>pIC50= 5.699 M;<br>[11]	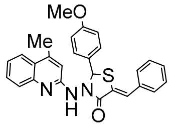<br>**066**<br>IC50= 1.414 μM;<br>pIC50= 6.000 M;<br>[11]
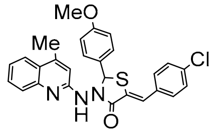<br>**067**<br>IC50= 0.632 μM;<br>pIC50= 6.199 M;<br>[11]	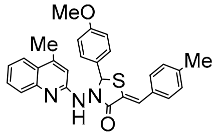<br>**068**<br>IC50= 1.536 μM;<br>pIC50= 5.699 M;<br>[11]	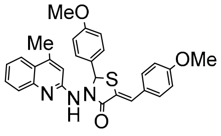<br>**069**<br>IC50= 1.801 μM;<br>pIC50= 5.699 M;<br>[11]
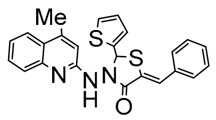<br>**070**<br>IC50= 1.931 μM;<br>pIC50= 5.699 M;<br>[11]	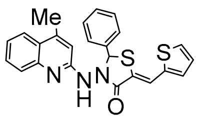<br>**071**<br>IC50= 2.672 μM;<br>pIC50= 5.523 M;<br>[11]	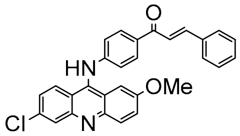<br>**072**<br>IC50= 0.75 μM;<br>pIC50= 6.125 M;<br>[12]
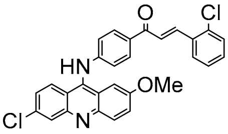<br>**073**<br>IC50= 1.5 μM;<br>pIC50= 5.824 M;<br>[12]	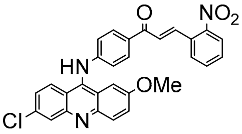<br>**074**<br>IC50= 0.35 μM;<br>pIC50= 6.456 M;<br>[12]	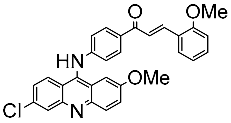<br>**075**<br>IC50= 0.52 μM;<br>pIC50= 6.284 M;<br>[12]

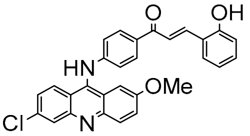<br>**076**<br>IC50= 4.5 μM;<br>pIC50= 5.347 M;<br>[12]	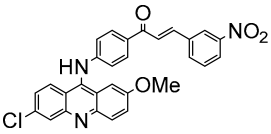<br>**077**<br>IC50= 1.3 μM;<br>pIC50= 5.886 M;<br>[12]	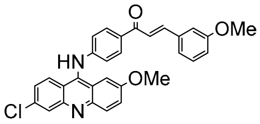<br>**078**<br>IC50= 1.8 μM;<br>pIC50= 5.745 M;<br>[12]
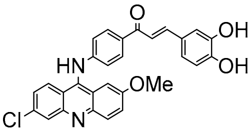<br>**079**<br>IC50= 2.0 μM;<br>pIC50= 5.699 M;<br>[12]	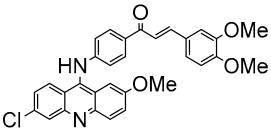<br>**080**<br>IC50= 4.0 μM;<br>pIC50= 5.398 M;<br>[12]	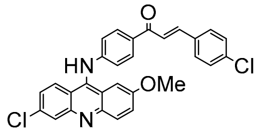<br>**081**<br>IC50= 1.3 μM;<br>pIC50= 5.886 M;<br>[12]
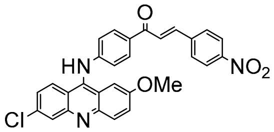<br>**082**<br>IC50= 0.7 μM;<br>pIC50= 6.155 M;<br>[12]	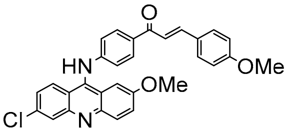<br>**083**<br>IC50= 0.3 μM;<br>pIC50= 6.523 M;<br>[12]	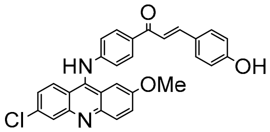<br>**084**<br>IC50= 0.8 μM;<br>pIC50= 6.097 M;<br>[12]
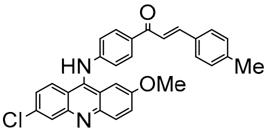<br>**085**<br>IC50= 4.8 μM;<br>pIC50= 5.319 M;<br>[12]	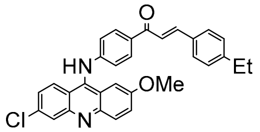<br>**086**<br>IC50= 2.4 μM;<br>pIC50= 5.620 M;<br>[12]	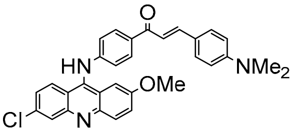<br>**087**<br>IC50= 2.5 μM;<br>pIC50= 5.602 M;<br>[12]
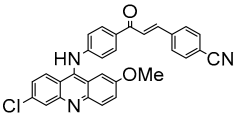<br>**088**<br>IC50= 1.7 μM;<br>pIC50= 5.770 M;<br>[12]	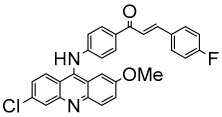<br>**089**<br>IC50= 2.7 μM;<br>pIC50= 5.569 M;<br>[12]	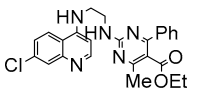<br>**090**<br>IC50= 0.0217 μM;<br>pIC50= 7.664 M;<br>[13]
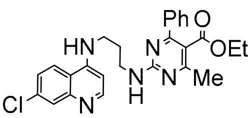<br>**091**<br>IC50= 0.2475 μM;<br>pIC50= 6.606 M;<br>[13]	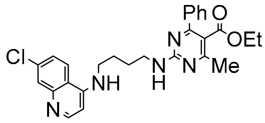<br>**092**<br>IC50= 0.2023 μM;<br>pIC50= 6.694 M;<br>[13]	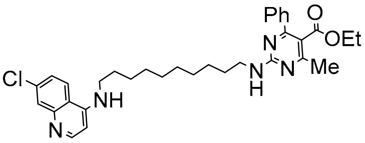<br>**093**<br>IC50= 1.7768 μM;<br>pIC50= 5.699 M;<br>[13]

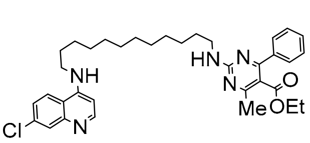<br>**094**<br>IC_{50}= 0.2648 μM;<br>pIC_{50}= 6.577 M;<br>[13]	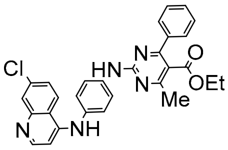<br>**095**<br>IC_{50}= 0.6444 μM;<br>pIC_{50}= 6.191 M;<br>[13]	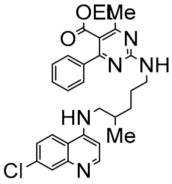<br>**096**<br>IC_{50}= 0.2867 μM;<br>pIC_{50}= 6.543 M;<br>[13]
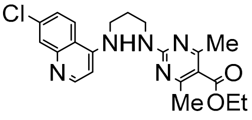<br>**097**<br>IC_{50}= 0.0631 μM;<br>pIC_{50}= 7.200 M;<br>[13]	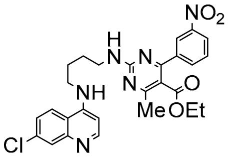<br>**098**<br>IC_{50}= 0.6972 μM;<br>pIC_{50}= 6.157 M;<br>[13]	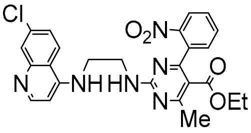<br>**099**<br>IC_{50}= 0.1729 μM;<br>pIC_{50}= 6.762 M;<br>[13]
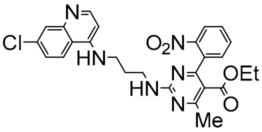<br>**100**<br>IC_{50}= 1.9551 μM;<br>pIC_{50}= 5.699 M;<br>[13]	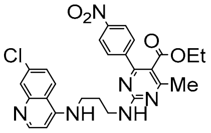<br>**101**<br>IC_{50}= 0.4999 μM;<br>pIC_{50}= 6.301 M;<br>[13]	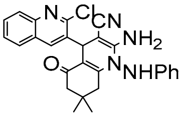<br>**102**<br>IC_{50}= 0.015 μM;<br>pIC_{50}= 7.824 M;<br>[14]
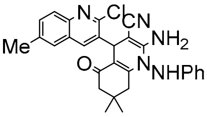<br>**103**<br>IC_{50}= 0.15<br>pIC_{50}= 6.824 M;<br>[14]	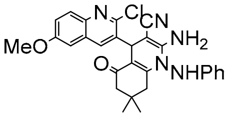<br>**104**<br>IC_{50}= 0.22<br>pIC_{50}= 6.658 M;<br>[14]	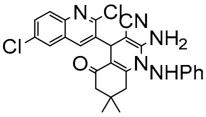<br>**105**<br>IC_{50}= 0.21<br>pIC_{50}= 6.678 M;<br>[14]
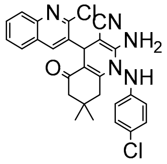<br>**106**<br>IC_{50}= 0.42 μM;<br>pIC_{50}= 6.377 M;<br>[14]	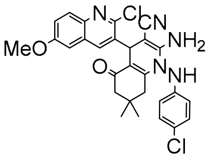<br>**107**<br>IC_{50}= 0.008 μM;<br>pIC_{50}= 8.097 M;<br>[14]	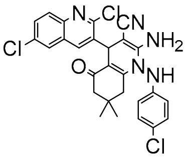<br>**108**<br>IC_{50}= 0.009 μM;<br>pIC_{50}= 8.046 M;<br>[14]
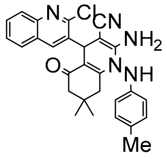<br>**109**<br>IC_{50}= 0.028 μM;<br>pIC_{50}= 7.553 M;<br>[14]	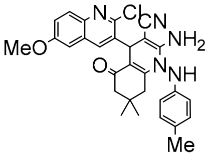<br>**110**<br>IC_{50}= 0.22 μM;<br>pIC_{50}= 6.658 M;<br>[14]	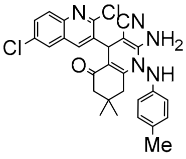<br>**111**<br>IC_{50}= 0.19 μM;<br>pIC_{50}= 6.721 M;<br>[14]

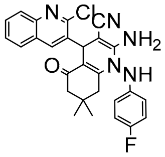<br>**112**<br>IC_{50}= 0.014 μM;<br>pIC_{50}= 7.854 M;<br>[14]	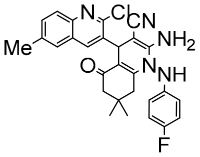<br>**113**<br>IC_{50}= 0.027 μM;<br>pIC_{50}= 7.569 M;<br>[14]	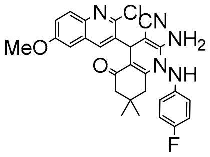<br>**114**<br>IC_{50}= 0.047 μM;<br>pIC_{50}= 7.328 M;<br>[14]
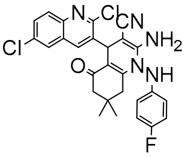<br>**115**<br>IC_{50}= 0.25 μM;<br>pIC_{50}= 6.602 M;<br>[14]	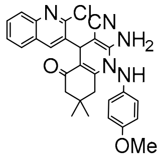<br>**116**<br>IC_{50}= 0.154 μM;<br>pIC_{50}= 6.813 M;<br>[14]	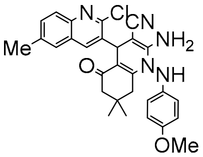<br>**117**<br>IC_{50}= 0.025 μM;<br>pIC_{50}= 7.602 M;<br>[14]
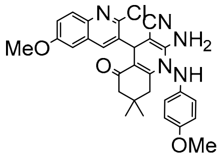<br>**118**<br>IC_{50}= 0.54 μM;<br>pIC_{50}= 6.268 M;<br>[14]	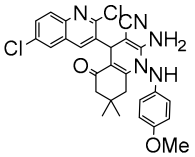<br>**119**<br>IC_{50}= 0.019 μM;<br>pIC_{50}= 7.721 M;<br>[14]	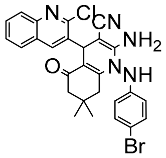<br>**120**<br>IC_{50}= 0.026 μM;<br>pIC_{50}= 7.585 M;<br>[14]
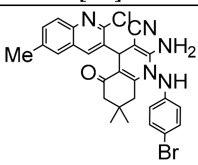<br>**121**<br>IC_{50}= 0.012 μM;<br>pIC_{50}= 7.921 M;<br>[14]	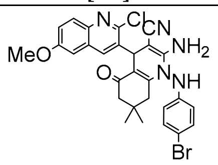<br>**122**<br>IC_{50}= 0.014 μM;<br>pIC_{50}= 7.854 M;<br>[14]	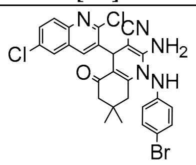<br>**123**<br>IC_{50}= 0.08 μM;<br>pIC_{50}= 7.097 M;<br>[14]
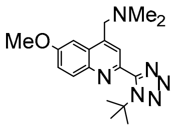<br>**124**<br>IC_{50}= 1.310 μM;<br>pIC_{50}= 6.000 M;<br>[15]	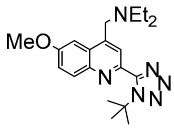<br>**125**<br>IC_{50}= 2.393 μM;<br>pIC_{50}= 5.699 M;<br>[15]	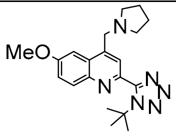<br>**126**<br>IC_{50}= 13.84 μM;<br>pIC_{50}= 4.859 M;<br>[15]
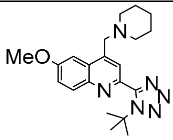<br>**127**<br>IC_{50}= 0.647 μM;<br>pIC_{50}= 6.189 M;<br>[15]	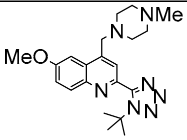<br>**128**<br>IC_{50}= 16.790 μM;<br>pIC_{50}= 4.770 M;<br>[15]	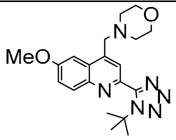<br>**129**<br>IC_{50}= 0.980 μM;<br>pIC_{50}= 6.009 M;<br>[15]
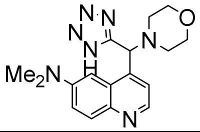	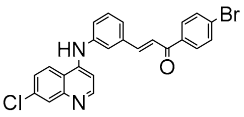	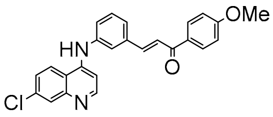

**130**<br>IC_{50}= 8.625 μM;<br>pIC_{50}= 5.046 M;<br>[15]	**131**<br>IC_{50}= 10.32 μM;<br>pIC_{50}= 4.986 M;<br>[16]	**132**<br>IC_{50}= 10.54 μM;<br>pIC_{50}= 4.977 M;<br>[16]
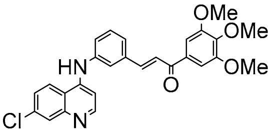<br>**133**<br>IC_{50}= 14.12 μM;<br>pIC_{50}= 4.850 M;<br>[16]	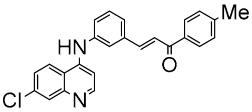<br>**134**<br>IC_{50}= 10.26 μM;<br>pIC_{50}= 4.989 M;<br>[16]	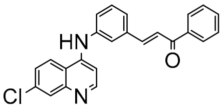<br>**135**<br>IC_{50}= 10.26 μM;<br>pIC_{50}= 4.989 M;<br>[16]
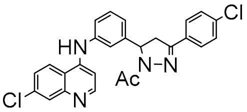<br>**136**<br>IC_{50}= 26.33 μM;<br>pIC_{50}= 4.580 M;<br>[16]	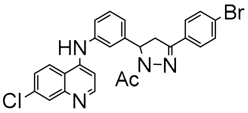<br>**137**<br>IC_{50}= 50.08 μM;<br>pIC_{50}= 4.300 M;<br>[16]	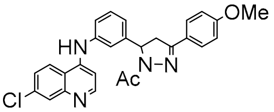<br>**138**<br>IC_{50}= 13.82 μM;<br>pIC_{50}= 4.860 M;<br>[16]
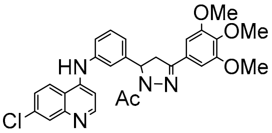<br>**139**<br>IC_{50}= 22.95 μM;<br>pIC_{50}= 4.639 M;<br>[16]	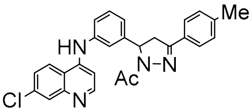<br>**140**<br>IC_{50}= 17.51 μM;<br>pIC_{50}= 4.757 M;<br>[16]	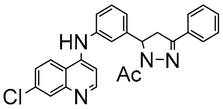<br>**141**<br>IC_{50}= 12.66 μM;<br>pIC_{50}= 4.898 M;<br>[16]
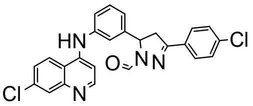<br>**142**<br>IC_{50}= 24.74 μM;<br>pIC_{50}= 4.607 M;<br>[16]	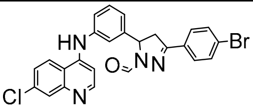<br>**143**<br>IC_{50}= 15.68 μM;<br>pIC_{50}= 4.805 M;<br>[16]	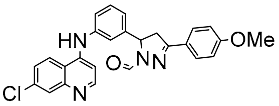<br>**144**<br>IC_{50}= 16.13 μM;<br>pIC_{50}= 4.792 M;<br>[16]
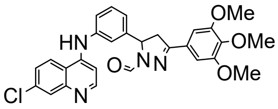<br>**145**<br>IC_{50}= 24.28 μM;<br>pIC_{50}= 4.615 M;<br>[16]	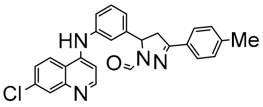<br>**146**<br>IC_{50}= 16.20 μM;<br>pIC_{50}= 4.791 M;<br>[16]	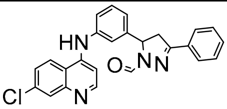<br>**147**<br>IC_{50}= 17.67 μM;<br>pIC_{50}= 4.753 M;<br>[16]
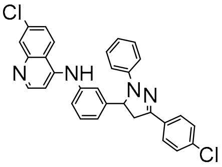<br>**148**<br>IC_{50}= 11.71 μM;<br>pIC_{50}= 4.931 M;<br>[16]	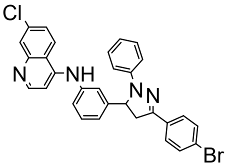<br>**149**<br>IC_{50}= 5.54 μM;<br>pIC_{50}= 5.257 M;<br>[16]	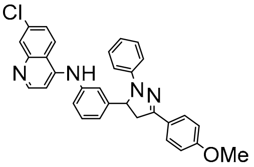<br>**150**<br>IC_{50}= 13.66 μM;<br>pIC_{50}= 4.865 M;<br>[16]

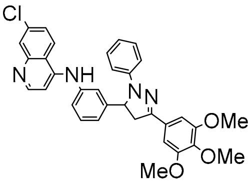<br>**151**<br>IC_{50}= 15.50 μM;<br>pIC_{50}= 4.810 M;<br>[16]	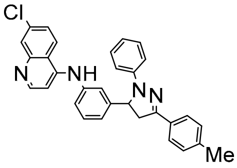<br>**152**<br>IC_{50}= 13.53 μM;<br>pIC_{50}= 4.869 M;<br>[16]	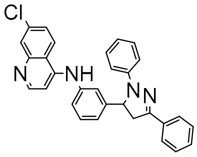<br>**153**<br>IC_{50}= 13.36 μM;<br>pIC_{50}= 4.874 M;<br>[16]
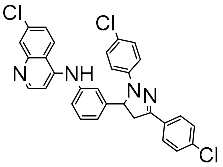<br>**154**<br>IC_{50}= 10.26 μM;<br>pIC_{50}= 4.989 M;<br>[16]	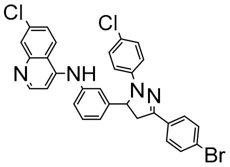<br>**155**<br>IC_{50}= 10.26 μM;<br>pIC_{50}= 4.989 M;<br>[16]	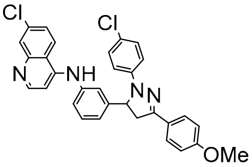<br>**156**<br>IC_{50}= 44.56 μM;<br>pIC_{50}= 4.351 M;<br>[16]
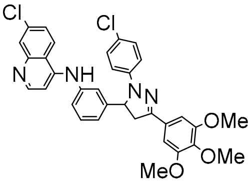<br>**157**<br>IC_{50}= 44.56 μM;<br>pIC_{50}= 4.351 M;<br>[16]	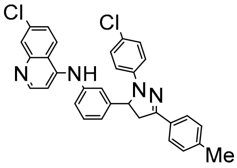<br>**158**<br>IC_{50}= 13.69 μM;<br>pIC_{50}= 4.864 M;<br>[16]	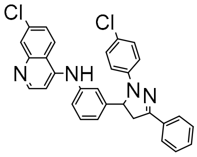<br>**159**<br>IC_{50}= 10.26 μM;<br>pIC_{50}= 4.989 M;<br>[16]
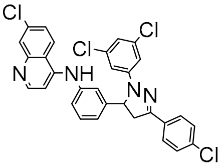<br>**160**<br>IC_{50}= 11.35 μM;<br>pIC_{50}= 4.945 M;<br>[16]	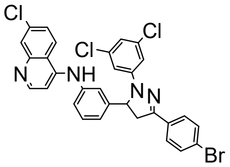<br>**161**<br>IC_{50}= 14.40 μM;<br>pIC_{50}= 4.842 M;<br>[16]	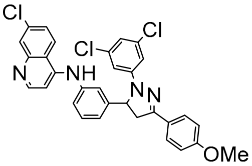<br>**162**<br>IC_{50}= 10.39 μM;<br>pIC_{50}= 4.983 M;<br>[16]
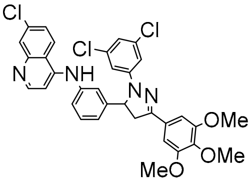<br>**163**<br>IC_{50}= 10.89 μM;<br>pIC_{50}= 4.963 M;<br>[16]	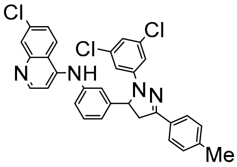<br>**164**<br>IC_{50}= 65.17 μM;<br>pIC_{50}= 4.186 M;<br>[16]	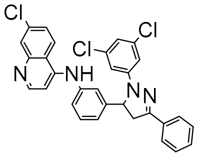<br>**165**<br>IC_{50}= 12.55 μM;<br>pIC_{50}= 4.901 M;<br>[16]
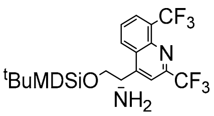<br>**166**<br>IC_{50}= 0.746 μM;<br>pIC_{50}= 6.127 M;<br>[17]	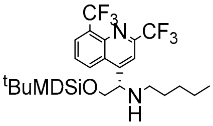<br>**167**<br>IC_{50}= 5.042 μM;<br>pIC_{50}= 5.301 M;<br>[17]	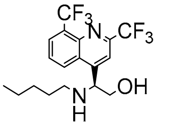<br>**168**<br>IC_{50}= 1.0535 μM;<br>pIC_{50}= 6.000 M;<br>[17]

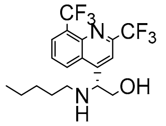<br>**169**<br>IC_{50}= 5.454 μM;<br>pIC_{50}= 5.301 M;<br>[17]	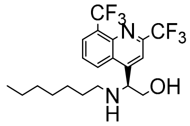<br>**170**<br>IC_{50}= 2.6541 μM;<br>pIC_{50}= 5.523 M;<br>[17]	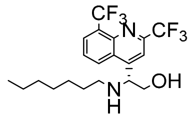<br>**171**<br>IC_{50}= 1.5532 μM;<br>pIC_{50}= 5.699 M;<br>[17]
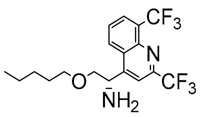<br>**172**<br>IC_{50}= 4.069 μM;<br>pIC_{50}= 5.398 M;<br>[17]	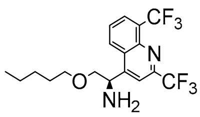<br>**173**<br>IC_{50}= 1.3605 μM;<br>pIC_{50}= 6.000 M;<br>[17]	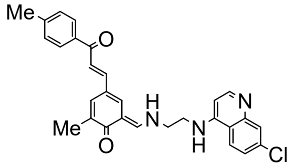<br>**174**<br>IC_{50}= 0.00002 μM;<br>pIC_{50}= 10.699 M;<br>[18]
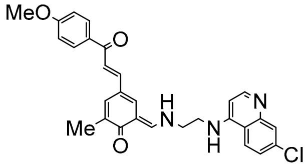<br>**175**<br>IC_{50}= 0.000033 μM;<br>pIC_{50}= 10.482 M;<br>[18]	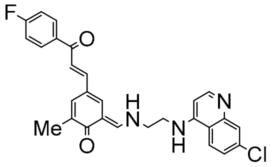<br>**176**<br>IC_{50}= 0.000022 μM;<br>pIC_{50}= 10.658 M;<br>[18]	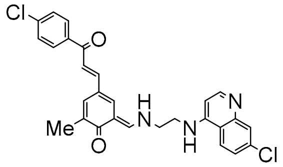<br>**177**<br>IC_{50}= 0.000044 μM;<br>pIC_{50}= 10.357 M;<br>[18]
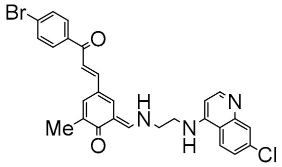<br>**178**<br>IC_{50}= 0.000034 μM;<br>pIC_{50}= 10.469 M;<br>[18]	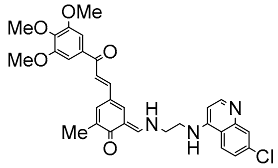<br>**179**<br>IC_{50}= 0.000019 μM;<br>pIC_{50}= 10.721 M;<br>[18]	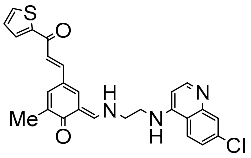<br>**180**<br>IC_{50}= 0.000021 μM;<br>pIC_{50}= 10.678 M;<br>[18]
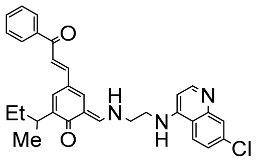<br>**181**<br>IC_{50}= 0.000027 μM;<br>pIC_{50}= 10.569 M;<br>[18]	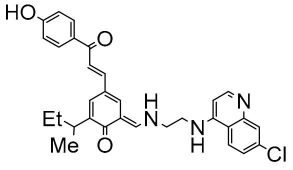<br>**182**<br>IC_{50}= 0.000042 μM;<br>pIC_{50}= 10.377 M;<br>[18]	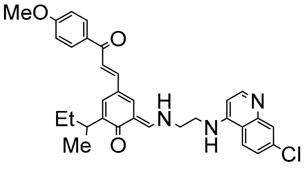<br>**183**<br>IC_{50}= 0.000034 μM;<br>pIC_{50}= 10.469 M;<br>[18]
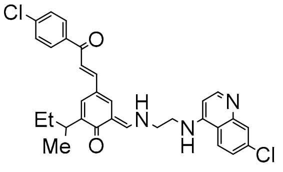<br>**184**<br>IC_{50}= 0.000033 μM;<br>pIC_{50}= 10.482 M;<br>[18]	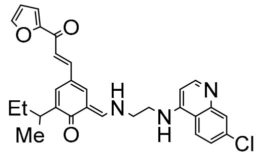<br>**185**<br>IC_{50}= 0.000014 μM;<br>pIC_{50}= 10.854 M;<br>[18]	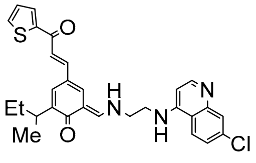<br>**186**<br>IC_{50}= 0.000018 μM;<br>pIC_{50}= 10.745 M;<br>[18]

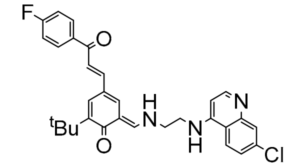<br>**187**<br>IC_{50}= 0.000014 μM;<br>pIC_{50}= 10.854 M;<br>[18]	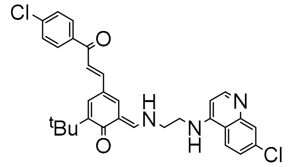<br>**188**<br>IC_{50}= 0.000052 μM;<br>pIC_{50}= 10.284 M;<br>[18]	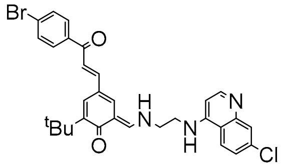<br>**189**<br>IC_{50}= 0.000008 μM;<br>pIC_{50}= 11.097 M;<br>[18]
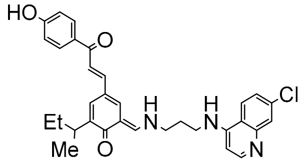<br>**190**<br>IC_{50}= 0.000078 μM;<br>pIC_{50}= 10.108 M;<br>[18]	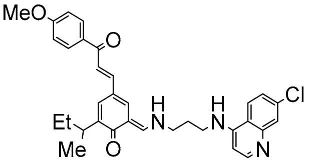<br>**191**<br>IC_{50}= 0.000075 μM;<br>pIC_{50}= 10.125 M;<br>[18]	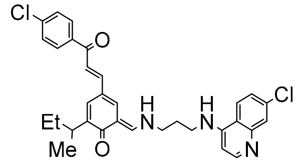<br>**192**<br>IC_{50}= 0.000075 μM;<br>pIC_{50}= 10.125 M;<br>[18]
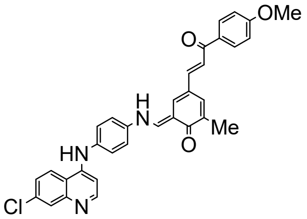<br>**193**<br>IC_{50}= 0.000366 μM;<br>pIC_{50}= 9.437 M;<br>[18]	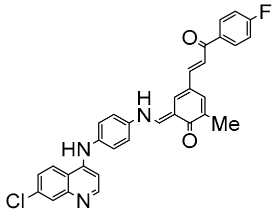<br>**194**<br>IC_{50}= 0.0003 μM;<br>pIC_{50}= 9.523 M;<br>[18]	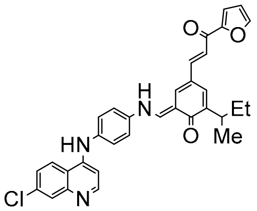<br>**195**<br>IC_{50}= 0.000146 μM;<br>pIC_{50}= 9.836 M;<br>[18]
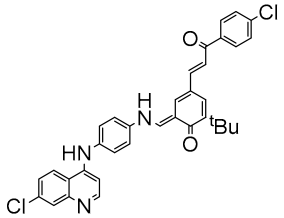<br>**196**<br>IC_{50}= 0.000641 μM;<br>pIC_{50}= 9.193 M;<br>[18]	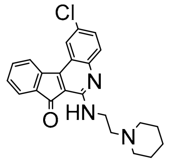<br>**197**<br>IC_{50}= 1713 μM;<br>pIC_{50}= 2.766 M;<br>[19]	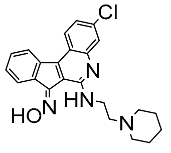<br>**198**<br>IC_{50}= 0.530 μM;<br>pIC_{50}= 6.276 M;<br>[19]
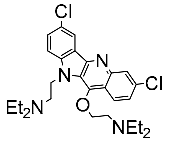<br>**199**<br>IC_{50}= 0.158 μM;<br>pIC_{50}= 6.801 M;<br>[19]	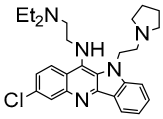<br>**200**<br>IC_{50}= 0.656 μM;<br>pIC_{50}= 6.183 M;<br>[19]	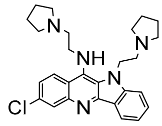<br>**201**<br>IC_{50}= 0.468 μM;<br>pIC_{50}= 6.330 M;<br>[19]

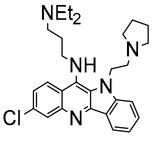<br>**202**<br>IC50= 0.48 μM;<br>pIC50= 6.319 M;<br>[19]	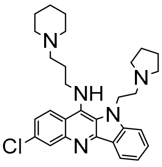<br>**203**<br>IC50= 0.535 μM;<br>pIC50= 6.272 M;<br>[19]	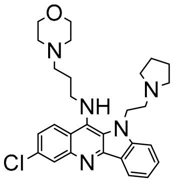<br>**204**<br>IC50= 0.469 μM;<br>pIC50= 6.329 M;<br>[19]
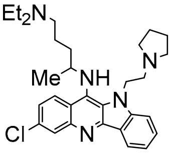<br>205<br>IC50= 0.245 μM;<br>pIC50= 6.611 M;<br>[19]	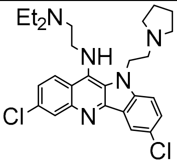<br>206<br>IC50= 0.245 μM;<br>pIC50= 6.611 M;<br>[19]	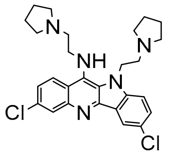<br>207<br>IC50= 0.375 μM;<br>pIC50= 6.426 M;<br>[19]
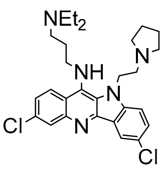<br>**208**<br>IC50= 0.392 μM;<br>pIC50= 6.407 M;<br>[19]	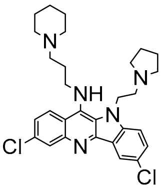<br>**209**<br>IC50= 0.66 μM;<br>pIC50= 6.181 M;<br>[19]	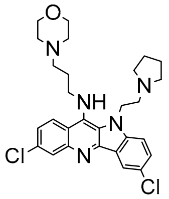<br>**210**<br>IC50= 0.407 μM;<br>pIC50= 6.390 M;<br>[19]
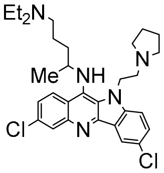<br>**211**<br>IC50= 0.456 μM;<br>pIC50= 6.341 M;<br>[19]	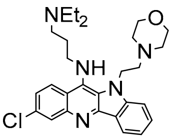<br>**212**<br>IC50= 0.32 μM;<br>pIC50= 6.495 M;<br>[19]	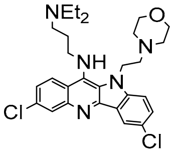<br>**213**<br>IC50= 0.199 μM;<br>pIC50= 6.701 M;<br>[19]
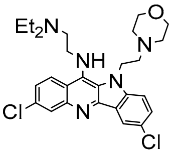<br>**214**<br>IC50= 0.513 μM;<br>pIC50= 6.290 M;<br>[19]	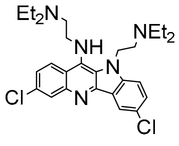<br>**215**<br>IC50= 0.397 μM;<br>pIC50= 6.401 M;<br>[19]	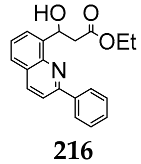<br>**216**<br>IC50= 45.1 μM;<br>pIC50= 4.346 M;<br>[20]
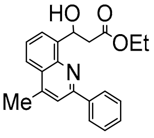<br>**217**<br>IC50= 34.3 μM;<br>pIC50= 4.465 M;<br>[20]	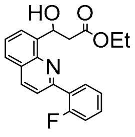<br>**218**<br>IC50= 47.1 μM;<br>pIC50= 4.327 M;<br>[20]	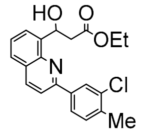<br>**219**<br>IC50= 27.1 μM;<br>pIC50= 4.567 M;<br>[20]

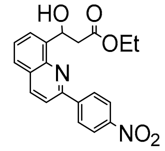<br>**220**<br>IC_{50}= 36.8 μM;<br>pIC_{50}= 4.434 M;<br>[20]	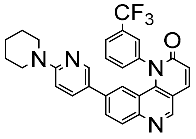<br>**221**<br>IC_{50}= 5.215 μM;<br>pIC_{50}= 5.301 M;<br>[21]	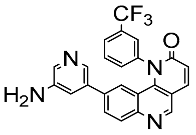<br>**222**<br>IC_{50}= 0.155 μM;<br>pIC_{50}= 6.810 M;<br>[21]
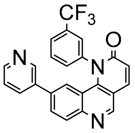<br>**223**<br>IC_{50}= 0.072 μM;<br>pIC_{50}= 7.143 M;<br>[21]	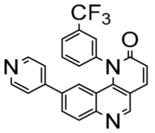<br>**224**<br>IC_{50}= 0.092 μM;<br>pIC_{50}= 7.036 M;<br>[21]	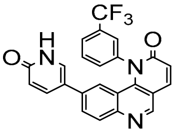<br>**225**<br>IC_{50}= 0.128 μM;<br>pIC_{50}= 6.893 M;<br>[21]
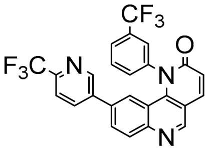<br>**226**<br>IC_{50}= 0.155 μM;<br>pIC_{50}= 6.810 M;<br>[21]	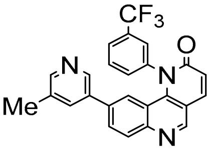<br>**227**<br>IC_{50}= 1.962 μM;<br>pIC_{50}= 5.699 M;<br>[21]	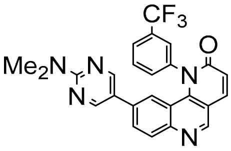<br>**228**<br>IC_{50}= 1.856 μM;<br>pIC_{50}= 5.699 M;<br>[21]
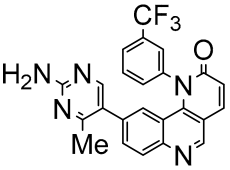<br>**229**<br>IC_{50}= 0.748 μM;<br>pIC_{50}= 6.126 M;<br>[21]	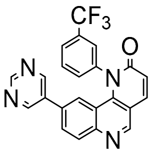<br>**230**<br>IC_{50}= 0.359 μM;<br>pIC_{50}= 6.445 M;<br>[21]	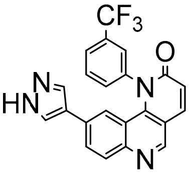<br>**231**<br>IC_{50}= 0.08 μM;<br>pIC_{50}= 7.097 M;<br>[21]
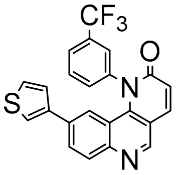<br>**232**<br>IC_{50}= 0.286 μM;<br>pIC_{50}= 6.544 M;<br>[21]	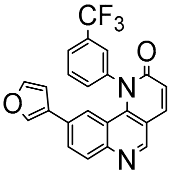<br>**233**<br>IC_{50}= 0.326 μM;<br>pIC_{50}= 6.487 M;<br>[21]	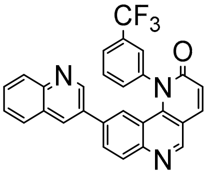<br>**234**<br>IC_{50}= 1.02 μM;<br>pIC_{50}= 5.991 M;<br>[21]
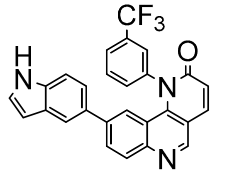<br>**235**<br>IC_{50}= 0.148 μM;<br>pIC_{50}= 6.830 M;<br>[21]	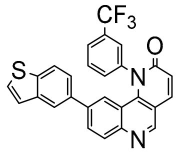<br>**236**<br>IC_{50}= 1.134 μM;<br>pIC_{50}= 6.000 M;<br>[21]	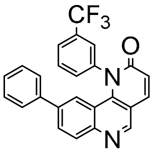<br>**237**<br>IC_{50}= 0.229 μM;<br>pIC_{50}= 6.640 M;<br>[21]

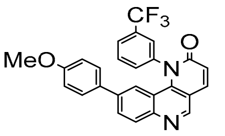<br>**238**<br>IC$_{50}$= 2.31 μM;<br>pIC$_{50}$= 5.636 M;<br>[21]	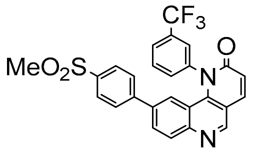<br>**239**<br>IC$_{50}$= 0.292 μM;<br>pIC$_{50}$= 6.535 M;<br>[21]	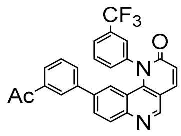<br>**240**<br>IC$_{50}$= 0.392 μM;<br>pIC$_{50}$= 6.407 M;<br>[21]
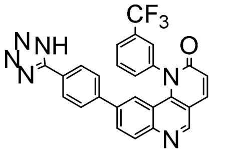<br>**241**<br>IC$_{50}$= 18.7 μM;<br>pIC$_{50}$= 4.728 M;<br>[21]	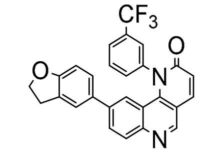<br>**242**<br>IC$_{50}$= 1.734 μM;<br>pIC$_{50}$= 5.699 M;<br>[21]	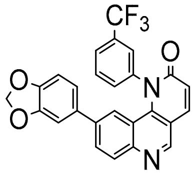<br>**243**<br>IC$_{50}$= 0.736 μM;<br>pIC$_{50}$= 6.133 M;<br>[21]
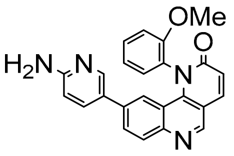<br>**244**<br>IC$_{50}$= 0.069 μM;<br>pIC$_{50}$= 7.161 M;<br>[21]	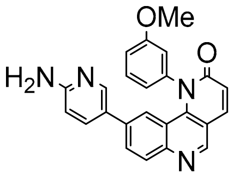<br>**245**<br>IC$_{50}$= 0.063 μM;<br>pIC$_{50}$= 7.201 M;<br>[21]	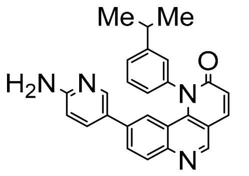<br>**246**<br>IC$_{50}$= 0.098 μM;<br>pIC$_{50}$= 7.009 M;<br>[21]
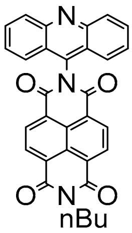<br>**247**<br>IC$_{50}$= 4.197 μM;<br>pIC$_{50}$= 5.398 M;<br>[22]	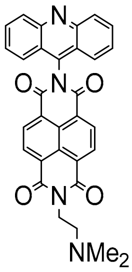<br>**248**<br>IC$_{50}$= 0.545 μM;<br>pIC$_{50}$= 6.264 M;<br>[22]	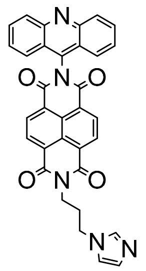<br>**249**<br>IC$_{50}$= 0.78 μM;<br>pIC$_{50}$= 6.108 M;<br>[22]
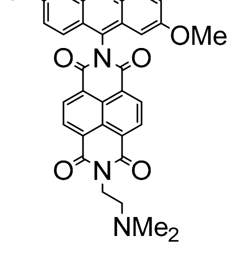<br>**250**<br>IC$_{50}$= 0.419 μM;<br>pIC$_{50}$= 6.378 M;<br>[22]	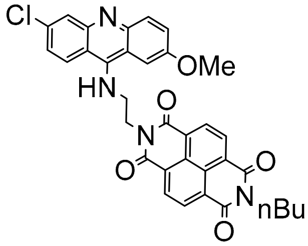<br>**251**<br>IC$_{50}$= 0.00365 μM;<br>pIC$_{50}$= 8.438 M;<br>[22]	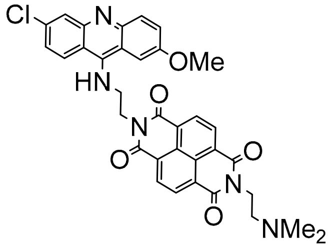<br>**252**<br>IC$_{50}$= 0.00433 μM;<br>pIC$_{50}$= 8.364 M;<br>[22]

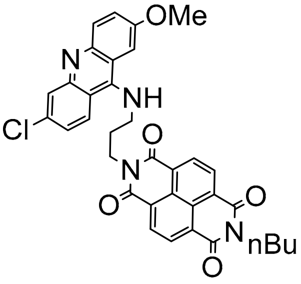<br>**253**<br>IC_{50}= 0.00389 μM;<br>pIC_{50}= 8.410 M;<br>[22]	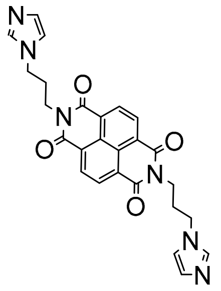<br>**254**<br>IC_{50}= 0.2602 μM;<br>pIC_{50}= 6.585 M;<br>[22]	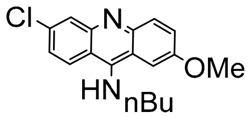<br>**255**<br>IC_{50}= 0.0264 μM;<br>pIC_{50}= 7.578 M;<br>[22]
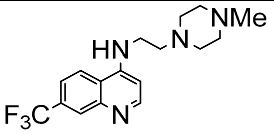<br>**256**<br>IC_{50}= 0.0251 μM;<br>pIC_{50}= 7.600 M;<br>[23]	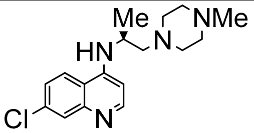<br>**257**<br>IC_{50}= 0.01688 μM;<br>pIC_{50}= 7.773 M;<br>[23]	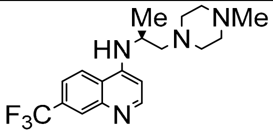<br>**258**<br>IC_{50}= 0.09215 μM;<br>pIC_{50}= 7.036 M;<br>[23]
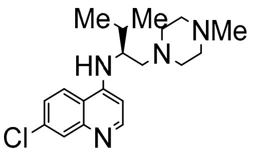<br>**259**<br>IC_{50}= 0.01518 μM;<br>pIC_{50}= 7.819 M;<br>[23]	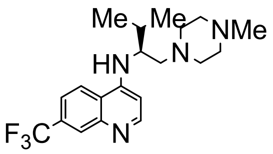<br>**260**<br>IC_{50}= 0.7 μM;<br>pIC_{50}= 6.155 M;<br>[23]	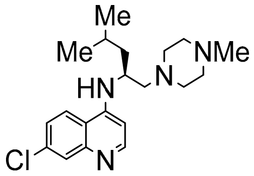<br>**261**<br>IC_{50}= 0.0088 μM;<br>pIC_{50}= 8.056 M;<br>[23]
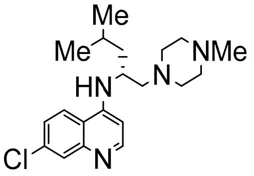<br>**262**<br>IC_{50}= 0.0109 μM;<br>pIC_{50}= 7.963 M;<br>[23]	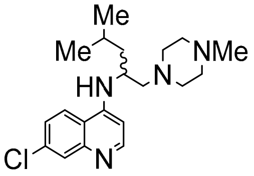<br>**263**<br>IC_{50}= 0.00836 μM;<br>pIC_{50}= 8.078 M;<br>[23]	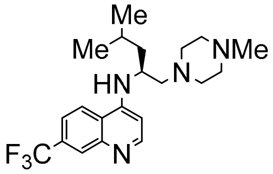<br>**264**<br>IC_{50}= 0.01136 μM;<br>pIC_{50}= 7.945 M;<br>[23]
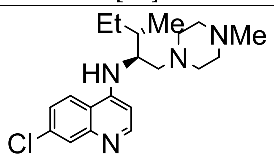<br>**265**<br>IC_{50}= 0.01469 μM;<br>pIC_{50}= 7.833 M;<br>[23]	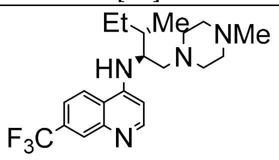<br>**266**<br>IC_{50}= 0.627 μM;<br>pIC_{50}= 6.203 M;<br>[23]	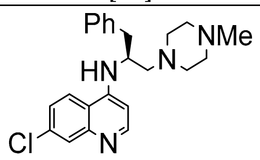<br>**267**<br>IC_{50}= 0.01027 μM;<br>pIC_{50}= 7.988 M;<br>[23]

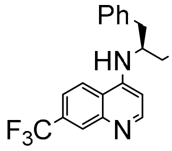<br>**268**<br>IC_{50}= 0.0053 μM;<br>pIC_{50}= 8.276 M;<br>[23]	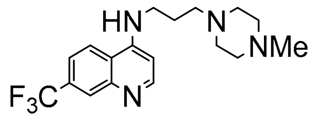<br>**269**<br>IC_{50}= 0.08122 μM;<br>pIC_{50}= 7.090 M;<br>[23]	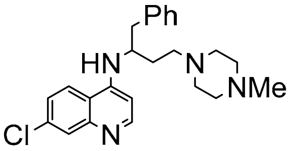<br>**270**<br>IC_{50}= 0.00503 μM;<br>pIC_{50}= 8.298 M;<br>[23]
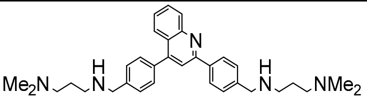<br>**271**<br>IC_{50}= 0.47 μM;<br>pIC_{50}= 6.328 M;<br>[24]	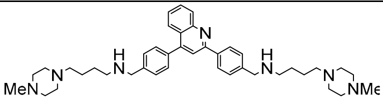<br>**272**<br>IC_{50}= 0.69 μM;<br>pIC_{50}= 6.161 M;<br>[24]	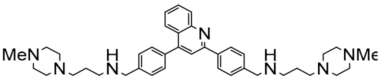<br>**273**<br>IC_{50}= 0.47 μM;<br>pIC_{50}= 6.328 M;<br>[24]
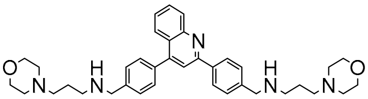<br>**274**<br>IC_{50}= 0.099 μM;<br>pIC_{50}= 7.004 M;<br>[24]	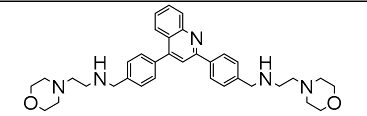<br>**275**<br>IC_{50}= 1.34 μM;<br>pIC_{50}= 5.873 M;<br>[24]	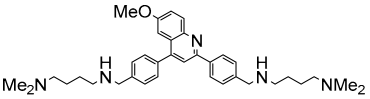<br>**276**<br>IC_{50}= 3.09 μM;<br>pIC_{50}= 5.510 M;<br>[24]
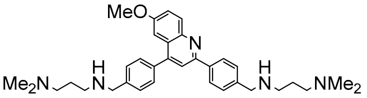<br>**277**<br>IC_{50}= 2.65 μM;<br>pIC_{50}= 5.577 M;<br>[24]	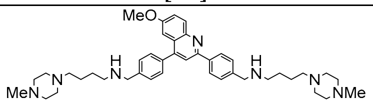<br>**278**<br>IC_{50}= 2.59 μM;<br>pIC_{50}= 5.587 M;<br>[24]	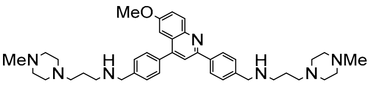<br>**279**<br>IC_{50}= 1.80 μM;<br>pIC_{50}= 5.745 M;<br>[24]
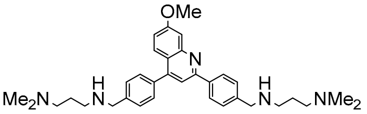<br>**280**<br>IC_{50}= 3.18 μM;<br>pIC_{50}= 5.498 M;<br>[24]	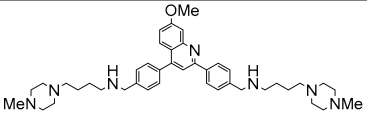<br>**281**<br>IC_{50}= 2.41 μM;<br>pIC_{50}= 5.618 M;<br>[24]	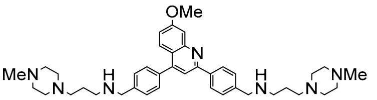<br>**282**<br>IC_{50}= 4.34 μM;<br>pIC_{50}= 5.363 M;<br>[24]
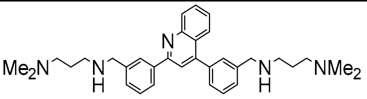<br>**283**<br>IC_{50}= 1.44 μM;<br>pIC_{50}= 5.842 M;<br>[24]	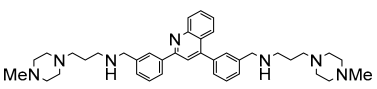<br>**284**<br>IC_{50}= 1.95 μM;<br>pIC_{50}= 5.710 M;<br>[24]	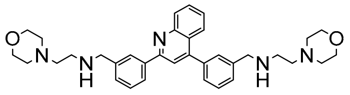<br>**285**<br>IC_{50}= 0.23 μM;<br>pIC_{50}= 6.638 M;<br>[24]
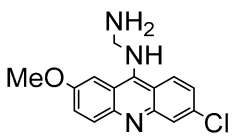<br>**286**<br>IC_{50}= 0.042 μM;<br>pIC_{50}= 7.377 M;<br>[25]	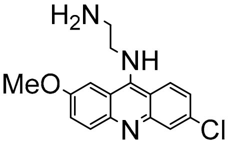<br>**287**<br>IC_{50}= 0.067 μM;<br>pIC_{50}= 7.174 M;<br>[25]	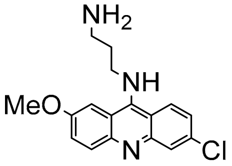<br>**288**<br>IC_{50}= 0.13 μM;<br>pIC_{50}= 6.886 M;<br>[25]

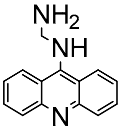<br>**289**<br>IC_{50}= 0.93 μM;<br>pIC_{50}= 6.032 M;<br>[25]	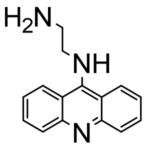<br>**290**<br>IC_{50}= 1.11 μM;<br>pIC_{50}= 5.955 M;<br>[25]	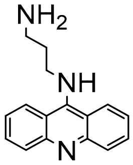<br>**291**<br>IC_{50}= 1.13 μM;<br>pIC_{50}= 5.947 M;<br>[25]
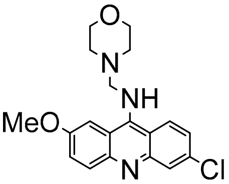<br>**298**<br>IC_{50}= 0.213 μM;<br>pIC_{50}= 6.672 M;<br>[25]	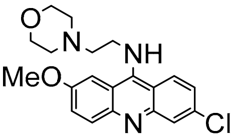<br>**299**<br>IC_{50}= 0.01 μM;<br>pIC_{50}= 8.000 M;<br>[25]	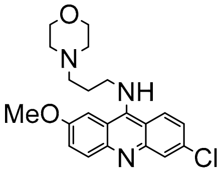<br>**300**<br>IC_{50}= 0.009 μM;<br>pIC_{50}= 8.046 M;<br>[25]
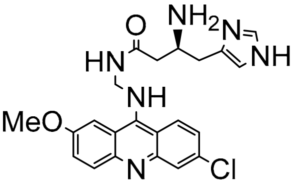<br>**301**<br>IC_{50}= 0.266 μM;<br>pIC_{50}= 6.575 M;<br>[25]	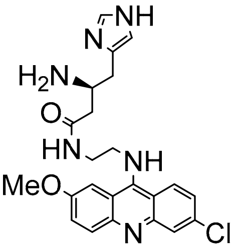<br>**302**<br>IC_{50}= 0.224 μM;<br>pIC_{50}= 6.650 M;<br>[25]	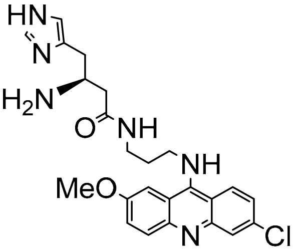<br>**303**<br>IC_{50}= 0.248 μM;<br>pIC_{50}= 6.606 M;<br>[25]
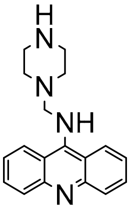<br>**295**<br>IC_{50}= 0.84 μM;<br>pIC_{50}= 6.076 M;<br>[25]	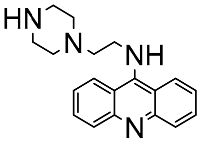<br>**296**<br>IC_{50}= 1.7 μM;<br>pIC_{50}= 5.770 M;<br>[25]	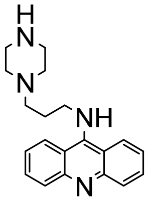<br>**297**<br>IC_{50}= 1.1 μM;<br>pIC_{50}= 5.959 M;<br>[25]
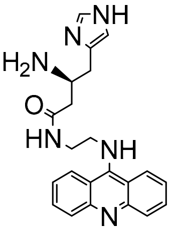<br>**304**<br>IC_{50}= 1.47 μM;<br>pIC_{50}= 5.833 M;<br>[25]	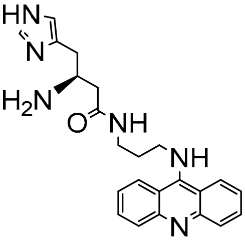<br>**305**<br>IC_{50}= 2.366 μM;<br>pIC_{50}= 5.699 M;<br>[25]	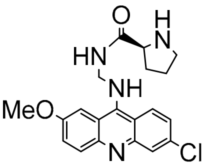<br>**306**<br>IC_{50}= 0.042 μM;<br>pIC_{50}= 7.377 M;<br>[25]

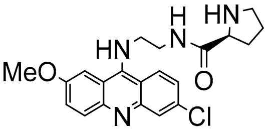<br>**307**<br>IC_{50}= 0.208 μM;<br>pIC_{50}= 6.682 M;<br>[25]	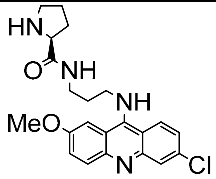<br>**308**<br>IC_{50}= 0.38 μM;<br>pIC_{50}= 6.420 M;<br>[25]	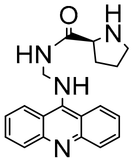<br>**309**<br>IC_{50}= 0.484 μM;<br>pIC_{50}= 6.315 M;<br>[25]
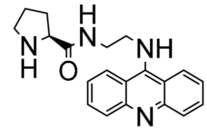<br>**310**<br>IC_{50}= 1.96 μM;<br>pIC_{50}= 5.708 M;<br>[25]	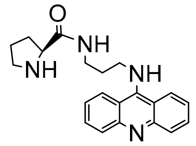<br>**311**<br>IC_{50}= 1.4 μM;<br>pIC_{50}= 5.854 M;<br>[25]	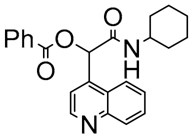<br>**312**<br>IC_{50}= 1.511 μM;<br>pIC_{50}= 5.699 M;<br>[26]
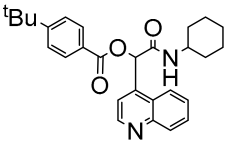<br>**313**<br>IC_{50}= 2.635 μM;<br>pIC_{50}= 5.523 M;<br>[26]	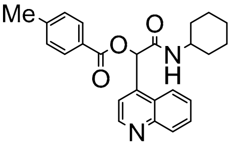<br>**314**<br>IC_{50}= 2.781 μM;<br>pIC_{50}= 5.523 M;<br>[26]	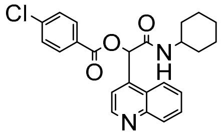<br>**315**<br>IC_{50}= 5.017 μM;<br>pIC_{50}= 5.301 M;<br>[26]
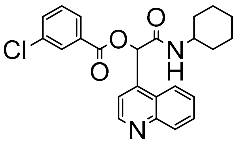<br>**316**<br>IC_{50}= 6.413 μM;<br>pIC_{50}= 5.222 M;<br>[26]	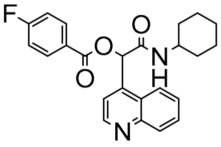<br>**317**<br>IC_{50}= 7.255 μM;<br>pIC_{50}= 5.155 M;<br>[26]	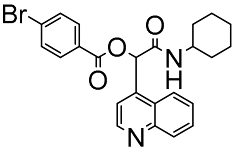<br>**318**<br>IC_{50}= 5.792 μM;<br>pIC_{50}= 5.222 M;<br>[26]
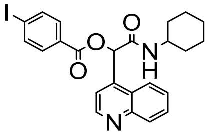<br>**319**<br>IC_{50}= 3.497 μM;<br>pIC_{50}= 5.523 M;<br>[26]	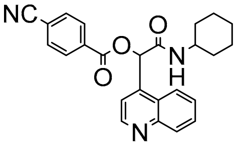<br>**320**<br>IC_{50}= 3.645 μM;<br>pIC_{50}= 5.398 M;<br>[26]	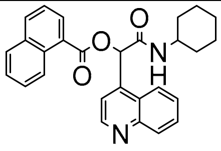<br>**321**<br>IC_{50}= 3.7 μM;<br>pIC_{50}= 5.432 M;<br>[26]
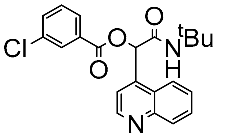<br>**325**<br>IC_{50}= 6.417 μM;<br>pIC_{50}= 5.222 M;<br>[26]	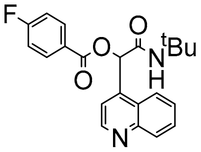<br>**326**<br>IC_{50}= 5.056 μM;<br>pIC_{50}= 5.301 M;<br>[26]	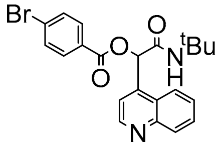<br>**327**<br>IC_{50}= 5.103 μM;<br>pIC_{50}= 5.301 M;<br>[26]

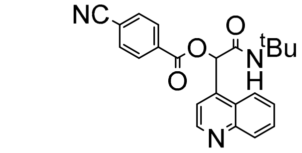

328
IC_{50}= 3.085 μM;
pIC_{50}= 5.523 M;
[26]

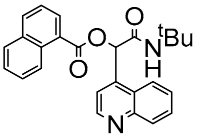

329
IC_{50}= 6.953 μM;
pIC_{50}= 5.155 M;
[26]

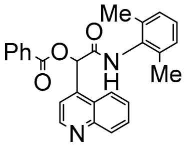

330
IC_{50}= 4.314 μM;
pIC_{50}= 5.398 M;
[26]

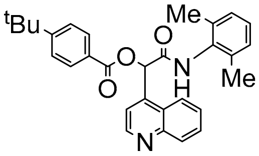

331
IC_{50}= 1.373 μM;
pIC_{50}= 6.000 M;
[26]

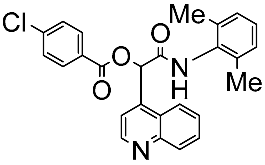

332
IC_{50}= 4.177 μM;
pIC_{50}= 5.398 M;
[26]

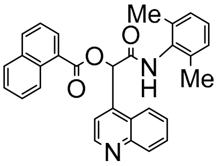

333
IC_{50}= 1.325 μM;
pIC_{50}= 6.000 M;
[26]

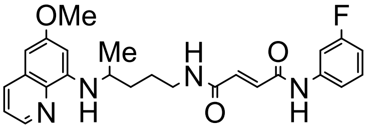

334
IC_{50}= 13.77 μM;
pIC_{50}= 4.861 M;
[27]

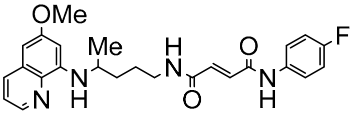

335
IC_{50}= 7.74 μM;
pIC_{50}= 5.111 M;
[27]

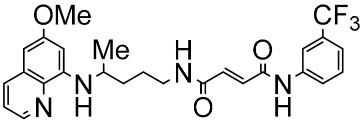

336
IC_{50}= 13.91 μM;
pIC_{50}= 4.857 M;
[27]

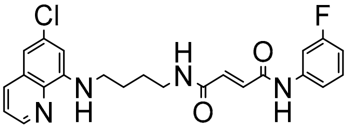

337
IC_{50}= 0.144 μM;
pIC_{50}= 6.842 M;
[27]

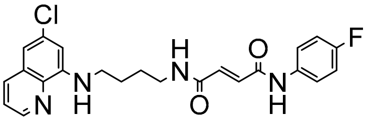

338
IC_{50}= 0.067 μM;
pIC_{50}= 7.174 M;
[27]

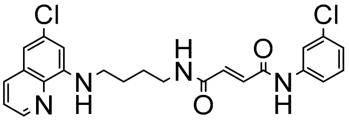

339
IC_{50}= 0.083 μM;
pIC_{50}= 7.081 M;
[27]

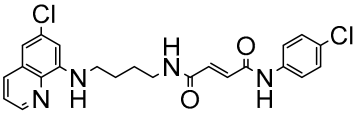

340
IC_{50}= 0.035 μM;
pIC_{50}= 7.456 M;
[27]

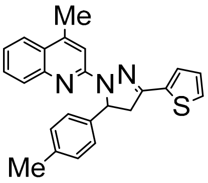

341
IC_{50}= 5.379 μM;
pIC_{50}= 5.301 M;
[7]

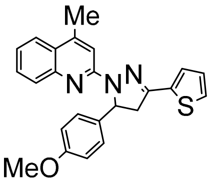

342
IC_{50}= 5.539 μM;
pIC_{50}= 5.222 M;
[7]

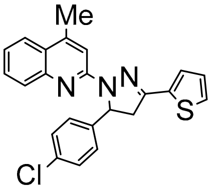

343
IC_{50}= 4.938 μM;
pIC_{50}= 5.301 M;
[7]

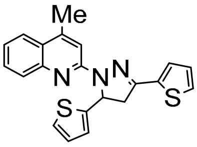

344
IC_{50}= 11.893 μM;
pIC_{50}= 4.921 M;
[7]

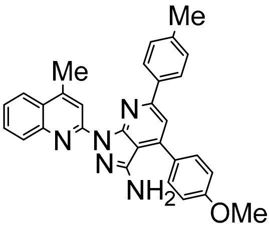

345
IC_{50}= 15076.433 μM;
pIC_{50}= 1.822 M;
[8]

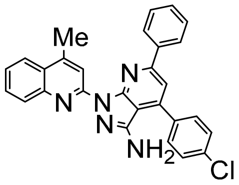<br>**346**<br>IC_{50}= 4763.557 μM;<br>pIC_{50}= 2.322 M;<br>[8]	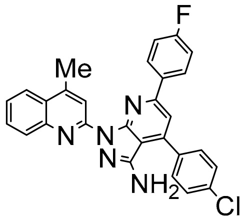<br>**347**<br>IC_{50}= 3620.042 μM;<br>pIC_{50}= 2.441 M;<br>[8]	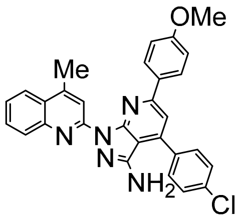<br>**348**<br>IC_{50}= 4038.697 μM;<br>pIC_{50}= 2.394 M;<br>[8]
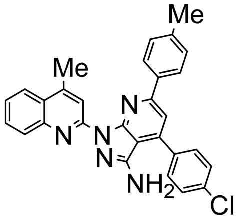<br>**349**<br>IC_{50}= 11252.632 μM;<br>pIC_{50}= 1.949 M;<br>[8]		

Table S2: Field combination of CoMFA and CoMSIA models of *P. falciparum* inhibitors

Model	q^2	N	SEP	SEE	r^2_{ncv}	F	Field Contributions				
							S	E	H	D	A
CoMFA-S	0.773	7	0.725	0.429	0.921	433.933	1				
CoMFA-E	-0.009	1	1.512	1.501	0.006	1.569		1			
**CoMFA-SE**	**0.765**	**12**	**0.795**	**0.400**	**0.932**	**294.497**	**0.951**	**0.049**			
CoMSIA-S	0.727	7	0.549	0.870	0.870	250.004	1				
CoMSIA-E	0	0	0	0	0	0		1			
CoMSIA-H	0.686	7	0.853	0.534	0.877	267.338			1		
CoMSIA-D	0.543	5	1.026	0.823	0.706	126.707				0	
CoMSIA-A	0.724	9	0.804	0.519	0.885	221.872					1
CoMSIA-SE	0.724	13	0.809	0.528	0.882	147.893	0.969	0.031			
CoMSIA-SEH	0.751	11	0.766	0.407	0.930	309.353	0.409	0.027	0.565		
CoMSIA-SEHD	0.746	9	0.770	0.412	0.927	369.075	0.245	0.025	0.390	0.340	
CoMSIA-SEHA	0.803	6	0.674	0.452	0.911	450.622	0.214	0.047	0.354		0.385
CoMSIA-SED	0.676	13	0.877	0.415	0.924	239.348	0.463	0.020		0.517	
CoMSIA-SEA	0.771	8	0.729	0.493	0.895	279.534	0.357	0.046			0.597
CoMSIA-SEDA	0.749	7	0.764	0.483	0.899	334.453	0.223	0.044		0.345	0.387
CoMSIA-SH	0.751	8	0.761	0.369	0.933	400.706	0.422		0.578		
CoMSIA-SD	0.680	10	0.865	0.422	0.924	314.392	0.475			0.525	
CoMSIA-SA	0.782	10	0.716	0.386	0.937	382.132	0.394				0.606
CoMSIA-SHD	0.746	8	0.769	0.415	0.926	408.420	0.251		0.401	0.348	
CoMSIA-SHA	0.701	6	0.678	0.431	0.919	499.926	0.228		0.365		0.407
CoMSIA-SDA	0.745	8	0.770	0.422	0.924	393.851	0.250			0.343	0.406
CoMSIA-SHDA	0.792	7	0.754	0.389	0.935	535.209	0.164		0.288	0.250	0.299
CoMSIA-EH	0.687	12	0.860	0.499	0.895	182.112		0.029	0.971		
CoMSIA-ED	0.545	7	1.027	0.806	0.720	96.166		0.073		0.927	
CoMSIA-EA	0.708	9	0.826	0.579	0.856	172.391		0.090			0.910
CoMSIA-EHD	0.725	10	0.803	0.428	0.922	305.158		0.031	0.515	0.454	
CoMSIA-EHA	0.738	7	0.685	0.447	0.914	397.571		0.065	0.445		0.491
CoMSIA-EDA	0.739	8	0.779	0.513	0.887	256.218		0.071		0.425	0.504
CoMSIA-EHDA	0.796	9	0.690	0.374	0.940	453.180		0.047	0.334	0.280	0.339
CoMSIA-HD	0.725	7	0.798	0.470	0.905	355.310			0.523	0.477	
CoMSIA-HA	0.800	7	0.681	0.409	0.928	480.589			0.480		0.520
CoMSIA-HDA	0.792	8	0.696	0.378	0.939	497.902			0.358	0.290	0.353
CoMSIA-DA	0.733	5	0.783	0.578	0.855	310.173				0.476	0.524
**CoMSIA-ALL**	**0.769**	**9**	**0.691**	**0.352**	**0.947**	**516.848**	**0.157**	**0.039**	**0.285**	**0.236**	**0.283**

q^2 = the square of the LOO cross-validation (CV) coefficient; N = the optimum number of components; SEP = standard error of prediction; SEE is the standard error of estimation of non CV analysis; r^2_{ncv} is the square of the non CV coefficient; F is the F-test value; S, E, H, D and A are the steric, electrostatic, hydrophobic, hydrogen-bond donor, and hydrogen-bond acceptor contributions respectively.

Table S3: Experimental and predicted pIC_{50} and residual values for analyzed compounds according to CoMFA and CoMSIA.

Compound	Experimental pIC_{50}	CoMFA		CoMSIA	
		Predicted pIC_{50}	Residual	Predicted pIC_{50}	Residual
1	5.538	6.399	-0.86	5.590	-0.05
2	7.435	7.367	0.07	7.059	0.38
3	7.104	6.883	0.22	6.779	0.33
4	6.952	6.883	0.07	6.779	0.17
5	7.318	6.987	0.33	7.023	0.30
6	6.601	6.987	-0.39	7.023	-0.42
7	7.043	7.045	0.00	7.145	-0.10
8	7.021	7.045	-0.02	7.145	-0.12
9^{t}	7.242	6.762	0.48	6.946	0.30
10^{t}	6.858	6.772	0.09	6.965	-0.11
11^{t}	6.530	7.100	-0.57	6.944	-0.41
12^{t}	6.892	6.628	0.26	6.571	0.32
13^{t}	6.185	5.897	0.29	5.611	0.57
14	7.377	7.299	0.08	6.885	0.49
15	6.920	7.495	-0.58	7.133	-0.21
16^{t}	6.963	6.762	0.20	7.063	-0.10
17	7.362	7.355	0.01	7.019	0.34
18	6.433	6.610	-0.18	6.862	-0.43
19^{t}	7.425	6.897	0.53	7.183	0.24
20	6.986	7.242	-0.26	6.943	0.04
21^{t}	6.618	7.185	-0.57	7.213	-0.60
22	6.907	6.963	-0.06	7.029	-0.12
23	6.758	7.022	-0.26	6.957	-0.20
24	6.542	6.739	-0.20	6.785	-0.24
25	7.692	7.892	-0.20	7.485	0.21
26	7.505	7.519	-0.01	7.656	-0.15
27	7.288	7.856	-0.57	7.282	0.01
28	7.128	7.039	0.09	7.062	0.07
29	7.014	6.881	0.13	6.856	0.16
30	7.404	6.950	0.45	7.129	0.28
31	7.636	7.449	0.19	7.373	0.26
32	6.512	6.489	0.02	6.648	-0.14
33	7.202	7.047	0.16	7.266	-0.06
34	7.118	7.523	-0.40	7.395	-0.28
35	6.900	6.607	0.29	6.747	0.15
36t	7.357	6.941	0.42	7.222	0.14
37	7.537	7.290	0.25	6.637	0.90
38^{t}	7.436	7.122	0.31	7.104	0.33
39	7.066	6.855	0.21	7.149	-0.08
40	7.052	7.456	-0.40	7.330	-0.28
41	7.305	6.610	0.69	6.899	0.41
42^{t}	7.161	6.620	0.54	6.642	0.52
43	6.690	7.243	-0.55	6.956	-0.27
44	7.472	7.056	0.42	7.115	0.36
45	7.153	7.135	0.02	7.113	0.04
46^{t}	7.107	7.379	-0.27	7.205	-0.10

47t	7.025	7.346	-0.32	6.934	0.09
48t	7.311	7.003	0.31	7.274	0.04
49	7.107	7.226	-0.12	7.181	-0.07
50	7.285	7.177	0.11	7.374	-0.09
51	7.366	7.448	-0.08	7.211	0.15
52	7.414	7.233	0.18	7.255	0.16
53	7.249	7.469	-0.22	7.099	0.15
54t	6.000	5.943	0.06	5.954	0.05
55	6.136	5.963	0.17	6.015	0.12
56	6.000	5.996	0.00	5.970	0.03
57	6.000	5.921	0.08	5.907	0.09
58	6.134	5.810	0.32	5.923	0.21
59	6.106	5.830	0.28	5.985	0.12
60	6.374	5.863	0.51	5.939	0.44
61	6.102	5.788	0.31	5.876	0.23
62	5.699	5.814	-0.12	5.925	-0.23
63	6.250	5.835	0.42	5.987	0.26
64	5.699	5.868	-0.17	5.942	-0.24
65	5.699	5.793	-0.09	5.879	-0.18
66	6.000	5.907	0.09	5.930	0.07
67	6.199	5.927	0.27	5.992	0.21
68	5.699	5.960	-0.26	5.947	-0.25
69	5.699	5.885	-0.19	5.883	-0.18
70	5.699	5.984	-0.29	6.029	-0.33
71	5.523	6.001	-0.48	5.931	-0.41
72t	6.125	5.968	0.16	5.778	0.35
73	5.824	5.995	-0.17	5.567	0.26
74	6.456	6.194	0.26	6.267	0.19
75	6.284	6.127	0.16	5.878	0.41
76	5.347	6.043	-0.70	5.553	-0.21
77	5.886	5.987	-0.10	5.765	0.12
78	5.745	5.989	-0.24	5.802	-0.06
79	5.699	6.203	-0.50	5.816	-0.12
80	5.398	5.828	-0.43	5.817	-0.42
81	5.886	5.886	0.00	5.684	0.20
82	6.155	5.833	0.32	5.591	0.56
83	6.523	5.807	0.72	5.792	0.73
84	6.097	5.943	0.15	6.144	-0.05
85	5.319	5.703	-0.38	5.658	-0.34
86	5.620	5.662	-0.04	5.616	0.00
87	5.602	5.724	-0.12	5.689	-0.09
88	5.770	5.900	-0.13	6.167	-0.40
89	5.569	5.965	-0.40	5.754	-0.19
90	7.664	7.139	0.52	7.653	0.01
91	6.606	6.577	0.03	7.055	-0.45
92	6.694	6.674	0.02	6.747	-0.05
93t	5.699	6.663	-0.96	6.801	-1.10
94	6.577	6.343	0.23	6.645	-0.07
95	6.191	6.138	0.05	5.839	0.35

96^t	6.543	5.847	0.70	6.882	-0.34
97	7.200	6.633	0.57	7.031	0.17
98	6.157	5.798	0.36	6.080	0.08
99	6.762	6.310	0.45	6.780	-0.02
100	5.699	5.692	0.01	5.648	0.05
101	6.301	6.656	-0.36	6.165	0.14
102	7.824	7.388	0.44	7.113	0.71
103	6.824	7.175	-0.35	7.148	-0.32
104^t	6.658	7.078	-0.42	7.101	-0.44
105	6.678	7.102	-0.42	7.094	-0.42
106	6.377	7.560	-1.18	7.212	-0.84
107^t	8.097	7.251	0.85	7.200	0.90
108	8.046	7.275	0.77	7.193	0.85
109^t	7.553	7.478	0.08	7.145	0.41
110	6.658	7.174	-0.52	7.136	-0.48
111	6.721	7.193	-0.47	7.127	-0.41
112	7.854	7.425	0.43	7.148	0.71
113	7.569	7.212	0.36	7.183	0.39
114^t	7.328	7.115	0.21	7.136	0.19
115	6.602	7.140	-0.54	7.129	-0.53
116	6.813	7.581	-0.77	7.120	-0.31
117	7.602	7.369	0.23	7.156	0.45
118	6.268	7.272	-1.00	7.108	-0.84
119	7.721	7.296	0.43	7.102	0.62
120	7.585	7.541	0.04	7.256	0.33
121	7.921	7.328	0.59	7.291	0.63
122	7.854	7.231	0.62	7.244	0.61
123	7.097	7.255	-0.16	7.237	-0.14
124	6.000	6.371	-0.37	6.014	-0.01
125	5.699	5.487	0.21	6.079	-0.38
126	4.859	5.235	-0.38	5.846	-0.99
127	6.189	5.537	0.65	5.838	0.35
128^t	4.770	5.702	-0.93	5.965	-1.20
129	6.009	6.061	-0.05	5.885	0.12
130	5.046	5.383	-0.34	5.368	-0.32
131	4.986	5.368	-0.38	5.323	-0.34
132^t	4.977	5.113	-0.14	4.966	0.01
133^t	4.850	5.362	-0.51	5.738	-0.89
134	4.989	5.264	-0.28	4.933	0.06
135	4.989	4.928	0.06	5.391	-0.40
136	4.580	4.696	-0.12	4.649	-0.07
137	4.300	4.735	-0.44	4.656	-0.36
138	4.860	4.675	0.19	4.691	0.17
139	4.639	4.810	-0.17	4.762	-0.12
140	4.757	4.617	0.14	4.617	0.14
141	4.898	4.569	0.33	4.612	0.29
142	4.607	4.799	-0.19	4.694	-0.09
143	4.805	4.839	-0.03	4.701	0.10
144	4.792	4.779	0.01	4.736	0.06

145	4.615	4.913	-0.30	4.807	-0.19
146t	4.791	4.858	-0.07	5.724	-0.93
147^{t}	4.753	4.673	0.08	4.658	0.09
148	4.931	4.703	0.23	4.750	0.18
149^{t}	5.257	4.716	0.54	4.772	0.48
150	4.865	4.702	0.16	4.680	0.19
151	4.810	5.014	-0.20	4.828	-0.02
152^{t}	4.869	4.435	0.43	4.843	0.03
153	4.874	4.706	0.17	4.697	0.18
154	4.989	4.902	0.09	4.748	0.24
155	4.989	4.914	0.08	4.769	0.22
156	4.351	4.901	-0.55	4.678	-0.33
157	4.351	4.465	-0.11	4.863	-0.51
158	4.864	4.634	0.23	4.841	0.02
159	4.989	4.905	0.08	4.694	0.30
160	4.945	4.831	0.11	4.755	0.19
161	4.842	4.843	0.00	4.777	0.06
162	4.983	4.830	0.15	4.685	0.30
163	4.963	5.141	-0.18	4.833	0.13
164	4.186	4.562	-0.38	4.848	-0.66
165	4.901	4.834	0.07	4.702	0.20
166	6.127	6.243	-0.12	6.394	-0.27
167^{t}	5.301	5.768	-0.47	5.769	-0.47
168	6.000	6.147	-0.15	6.044	-0.04
169^{t}	5.301	5.644	-0.34	6.090	-0.79
170	5.523	5.505	0.02	5.624	-0.10
171	5.699	5.557	0.14	5.692	0.01
172	5.398	5.306	0.09	5.487	-0.09
173	6.000	5.507	0.49	5.230	0.77
174	10.699	10.381	0.32	10.522	0.18
175	10.482	10.371	0.11	10.346	0.14
176	10.658	10.296	0.36	10.463	0.20
177	10.357	10.367	-0.01	10.555	-0.20
178^{t}	10.469	10.411	0.06	10.615	-0.15
179^{t}	10.721	10.354	0.37	10.330	0.39
180	10.678	10.258	0.42	10.315	0.36
181^{t}	10.569	10.886	-0.32	10.683	-0.11
182	10.377	10.917	-0.54	10.645	-0.27
183	10.469	10.300	0.17	10.395	0.07
184	10.482	10.966	-0.48	10.816	-0.33
185	10.854	9.891	0.96	10.705	0.15
186^{t}	10.745	10.686	0.06	10.545	0.20
187	10.854	10.723	0.13	10.675	0.18
188	10.284	10.794	-0.51	10.768	-0.48
189	11.097	10.838	0.26	10.827	0.27
190	10.108	9.935	0.17	10.522	-0.41
191	10.125	10.019	0.11	10.191	-0.07
192	10.125	10.044	0.08	10.247	-0.12
193	9.437	9.462	-0.03	9.471	-0.03

194	9.523	9.462	0.06	9.532	-0.01
195	9.836	9.564	0.27	9.294	0.54
196[t]	9.193	9.594	-0.40	9.689	-0.50
197	2.766	3.903	-1.14	3.674	-0.91
198	6.276	5.274	1.00	5.047	1.23
199[t]	6.801	7.143	-0.34	6.800	0.00
200	6.183	6.416	-0.23	6.179	0.00
201[t]	6.330	6.710	-0.38	6.947	-0.62
202	6.319	6.438	-0.12	6.310	0.01
203	6.272	6.013	0.26	6.535	-0.26
204	6.329	6.058	0.27	6.273	0.06
205	6.611	6.248	0.36	6.432	0.18
206[t]	6.611	6.357	0.25	6.162	0.45
207[t]	6.426	5.996	0.43	6.700	-0.27
208	6.407	6.179	0.23	6.552	-0.15
209	6.181	6.262	-0.08	6.334	-0.15
210	6.390	6.736	-0.35	6.502	-0.11
211	6.341	6.177	0.16	6.410	-0.07
212	6.495	6.693	-0.20	6.460	0.04
213	6.701	6.631	0.07	6.443	0.26
214	6.290	6.432	-0.14	6.329	-0.04
215	6.401	6.739	-0.34	6.427	-0.03
216[t]	4.346	4.146	0.20	4.591	-0.25
217[t]	4.465	4.009	0.46	4.383	0.08
218	4.327	4.028	0.30	4.583	-0.26
219	4.567	3.931	0.64	4.280	0.29
220	4.434	4.473	-0.04	4.458	-0.02
221	5.301	6.143	-0.84	5.901	-0.60
222[t]	6.810	6.446	0.36	6.426	0.38
223[t]	7.143	6.400	0.74	6.311	0.83
224[t]	7.036	6.360	0.68	6.233	0.80
225	6.893	6.414	0.48	6.666	0.23
226	6.810	6.410	0.40	6.053	0.76
227[t]	5.699	6.452	-0.75	6.336	-0.64
228	5.699	5.633	0.07	5.797	-0.10
229	6.126	6.402	-0.28	6.714	-0.59
230	6.445	6.392	0.05	6.196	0.25
231[t]	7.097	6.321	0.78	6.274	0.82
232	6.544	6.379	0.17	6.253	0.29
233[t]	6.487	6.343	0.14	6.254	0.23
234	5.991	5.985	0.01	6.148	-0.16
235	6.830	6.537	0.29	6.638	0.19
236	6.000	6.284	-0.28	6.135	-0.14
237[t]	6.640	6.416	0.22	6.208	0.43
238	5.636	5.530	0.11	6.056	-0.42
239[t]	6.535	5.633	0.90	6.097	0.44
240[t]	6.407	6.294	0.11	6.279	0.13
241[t]	4.728	5.579	-0.85	6.099	-1.37
242	5.699	6.277	-0.58	6.209	-0.51

243[t]	6.133	6.447	-0.31	6.194	-0.06
244	7.161	6.188	0.97	7.055	0.11
245[t]	7.201	6.319	0.88	6.983	0.22
246[t]	7.009	6.210	0.80	6.647	0.36
247	5.398	5.696	-0.30	5.707	-0.31
248[t]	6.264	6.445	-0.18	5.914	0.35
249	6.108	5.698	0.41	5.815	0.29
250	6.378	6.617	-0.24	6.649	-0.27
251	8.438	7.675	0.76	8.092	0.35
252	8.364	8.867	-0.50	8.740	-0.38
253[t]	8.410	7.675	0.74	8.092	0.32
254	6.585	6.639	-0.05	6.403	0.18
255[t]	7.578	6.957	0.62	7.240	0.34
256[t]	7.600	6.876	0.72	7.179	0.42
257	7.773	7.476	0.30	7.337	0.44
258	7.036	7.203	-0.17	7.334	-0.30
259	7.819	7.422	0.40	7.635	0.18
260	6.155	6.824	-0.67	7.303	-1.15
261	8.056	8.090	-0.03	7.847	0.21
262[t]	7.963	7.297	0.67	7.280	0.68
263	8.078	7.297	0.78	7.280	0.80
264	7.945	7.817	0.13	7.843	0.10
265[t]	7.833	7.170	0.66	7.580	0.25
266	6.203	7.009	-0.81	7.673	-1.47
267	7.988	8.285	-0.30	7.974	0.01
268	8.276	7.999	0.28	7.975	0.30
269	7.090	6.489	0.60	7.216	-0.13
270	8.298	7.770	0.53	8.224	0.07
271	6.328	6.079	0.25	5.858	0.47
272[t]	6.161	6.779	-0.62	5.950	0.21
273	6.328	6.123	0.21	6.156	0.17
274	7.004	6.808	0.20	7.106	-0.10
275[t]	5.873	5.743	0.13	6.337	-0.46
276	5.510	5.829	-0.32	5.763	-0.25
277	5.577	5.571	0.01	5.836	-0.26
278	5.587	5.591	0.00	5.622	-0.04
279[t]	5.745	6.334	-0.59	5.620	0.13
280	5.498	5.687	-0.19	5.308	0.19
281	5.618	5.916	-0.30	6.062	-0.44
282	5.363	5.883	-0.52	5.721	-0.36
283[t]	5.842	5.093	0.75	4.804	1.04
284	5.710	5.104	0.61	5.660	0.05
285	6.638	6.281	0.36	6.942	-0.30
286	7.377	7.573	-0.20	7.061	0.32
287[t]	7.174	7.192	-0.02	7.303	-0.13
288	6.886	6.884	0.00	7.184	-0.30
289	6.032	6.662	-0.63	6.308	-0.28
290[t]	5.955	6.396	-0.44	6.553	-0.60
291	5.947	5.916	0.03	6.049	-0.10

292	7.699	7.334	0.37	7.166	0.53
293	7.222	7.166	0.06	6.930	0.29
294	6.854	6.980	-0.13	6.751	0.10
295	6.076	6.273	-0.20	6.412	-0.34
296[t]	5.770	6.617	-0.85	6.556	-0.79
297	5.959	6.062	-0.10	5.997	-0.04
298	6.672	7.173	-0.50	6.915	-0.24
299[t]	8.000	7.479	0.52	7.329	0.67
300	8.046	7.118	0.93	7.794	0.25
301	6.575	5.675	0.90	6.436	0.14
302	6.650	6.857	-0.21	6.592	0.06
303	6.606	5.935	0.67	6.181	0.43
304	5.833	5.274	0.56	6.016	-0.18
305[t]	5.699	6.111	-0.41	6.359	-0.66
306[t]	7.377	7.184	0.19	6.918	0.46
307	6.682	6.496	0.19	6.560	0.12
308	6.420	6.844	-0.42	6.801	-0.38
309	6.315	6.244	0.07	6.414	-0.10
310	5.708	6.244	-0.54	5.720	-0.01
311	5.854	6.197	-0.34	5.938	-0.08
312[t]	5.699	5.361	0.34	5.217	0.48
313[t]	5.523	5.857	-0.33	5.497	0.03
314[t]	5.523	5.283	0.24	5.209	0.31
315	5.301	5.292	0.01	5.199	0.10
316	5.222	5.414	-0.19	5.297	-0.07
317	5.155	5.316	-0.16	5.210	-0.05
318	5.222	5.282	-0.06	5.191	0.03
319[t]	5.523	5.283	0.24	5.180	0.34
320	5.398	5.294	0.10	5.244	0.15
321	5.432	5.670	-0.24	5.463	-0.03
322	5.398	5.273	0.13	5.321	0.08
323	5.523	5.870	-0.35	6.011	-0.49
324	5.222	5.235	-0.01	5.248	-0.03
325	5.222	5.217	0.01	5.241	-0.02
326	5.301	5.257	0.04	5.299	0.00
327	5.301	5.237	0.06	5.214	0.09
328	5.523	5.241	0.28	5.315	0.21
329	5.155	5.194	-0.04	5.594	-0.44
330[t]	5.398	5.533	-0.14	5.202	0.20
331	6.000	5.637	0.36	5.210	0.79
332	5.398	5.544	-0.15	5.189	0.21
333	6.000	5.926	0.07	5.212	0.79
334[t]	4.861	5.979	-1.12	5.468	-0.61
335	5.111	5.058	0.05	4.992	0.12
336	4.857	5.061	-0.20	4.987	-0.13
337[t]	6.842	6.866	-0.02	7.005	-0.16
338	7.174	7.444	-0.27	7.154	0.02
339	7.081	6.882	0.20	7.047	0.03
340	7.456	7.556	-0.10	7.156	0.30

341	5.301	5.820	-0.52	6.370	-1.07
342	5.222	5.015	0.21	5.089	0.13
343	5.301	5.015	0.29	5.089	0.21
344	4.921	5.747	-0.83	5.449	-0.53
345	1.822	1.637	0.19	2.326	-0.50
346	2.322	1.893	0.43	2.235	0.09
347	2.441	2.288	0.15	2.105	0.34
348[t]	2.394	2.506	-0.11	2.475	-0.08
349	1.949	2.337	-0.39	2.035	-0.09

[t]: Compound used to the test set

Table S4: *Y*-randomization test for CoMFA and CoMSIA models

Iteración	CoMFA-SE		CoMSIA-All	
	q^2	r^2_{ncv}	q^2	r^2_{ncv}
**random_1**	-0.247	0.365	-0.354	0.231
**random_2**	-0.362	0.432	-0.036	0.118
**random_3**	-0.234	0.353	-0.364	0.324
**random_4**	-0.420	0.301	-0.273	0.365
**random_5**	-0.354	0.283	-0.321	0.287
**random_6**	-0.552	0.424	-0.014	0.523
**random_7**	-0.334	0.402	-0.103	0.364
**random_8**	-0.463	0.241	-0.367	0.427
**random_9**	-0.145	0.516	-0.423	0.325
**random_10**	-0.236	0.342	-0.526	0.324

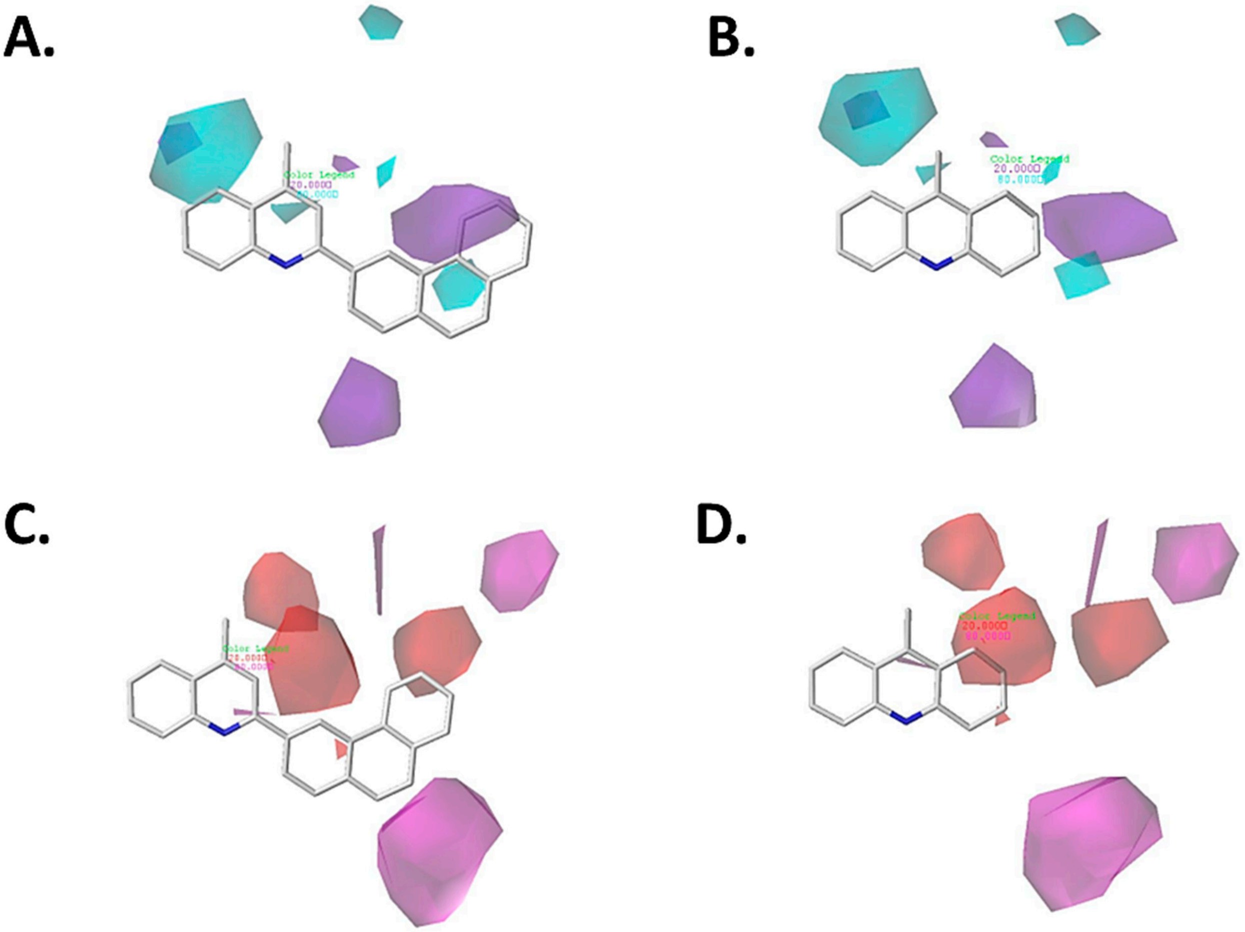

Figure S3: CoMSIA donor (A,B) and acceptor (C,D) contour maps around compounds **353** (left) and **356** (right), the most active and least active of the designed compounds series respectively. Color code: Donor and acceptor favored areas are in cyan and magenta respectively, and donor and acceptor disfavored areas are in purple and red, respectively.

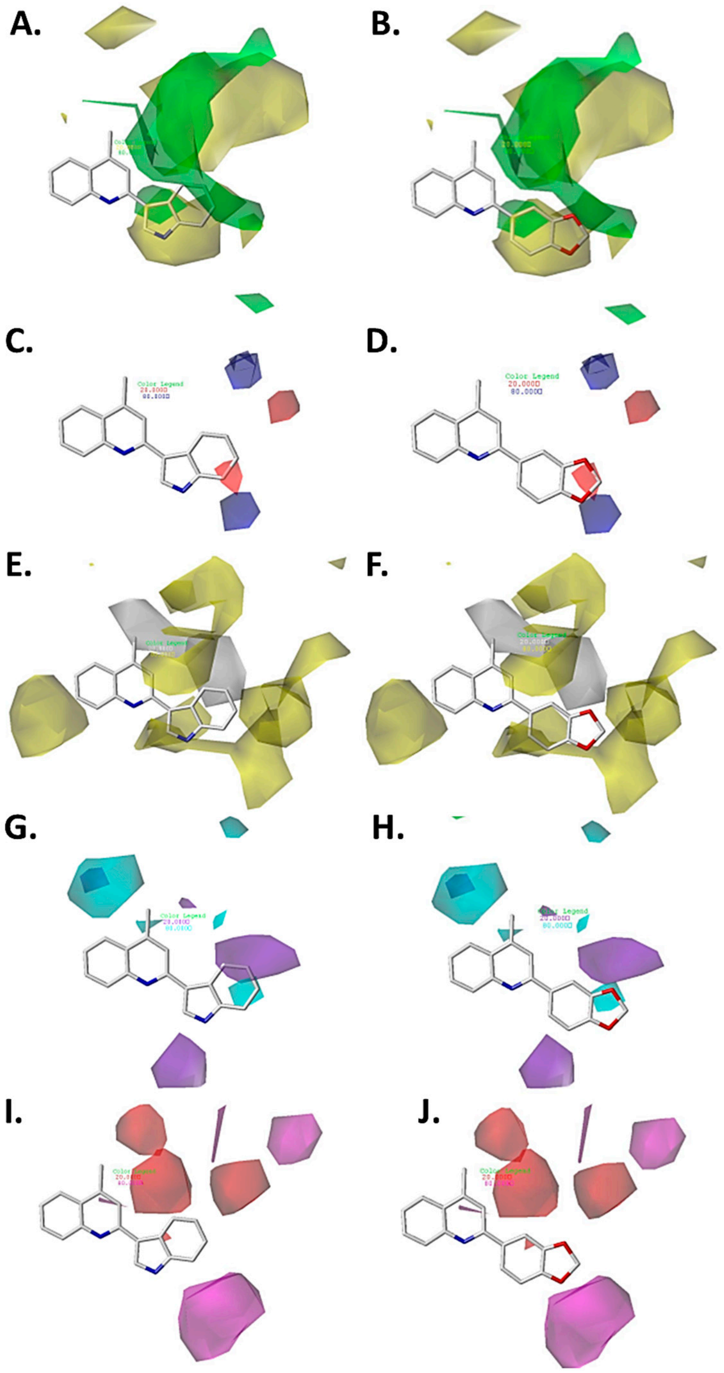

Figure S4: CoMSIA steric (A,B), electrostatic (C,D), hydrophobic (E,F), donor (G,H) and acceptor (I,J) contour maps around compounds **354** (left) and **351** (right), the most active and least active of the series respectively. Color code: Sterically favored areas are in green and disfavored areas are in yellow. Electropositive favoured areas are in blue and electronegative favoured areas are in red. Hydrophobic favored areas are in yellow and disfavored areas in grey. Donor and acceptor favored areas are in cyan and magenta respectively, and donor and acceptor disfavored areas are in purple and red, respectively.

Table S5. The SMILES codes for the compounds.

Molecule	SMILES	pIC_{50}
1	O=C(/C=C/C(NC1=CC=CC(F)=C1)=O)NCCCCNC2=CC(C(F)(F)F)=NC3=C(C(F)(F)F)C=CC=C32	5.538
2	ClC1=CC=C2C(N=CC=C2N/N=C/C3=CC=C(OCC(C4=CC=C(Cl)C=C4)=O)C=C3)=C1	7.435
3	ClC1=CC=C2C(N=CC=C2N/N=C/C3=CC=C(O/C(C(C4=CC=C(Cl)C=C4)=O)=C\C5=CC=CC=C5)C=C3)=C1	7.104
4	ClC1=CC=C2C(N=CC=C2N/N=C/C3=CC=C(O/C(C(C4=CC=C(Cl)C=C4)=O)=C\C5=CC=C(C)C=C5)C=C3)=C1	6.952
5	ClC1=CC=C2C(N=CC=C2N/N=C/C3=CC=C(O/C(C(C4=CC=C(Cl)C=C4)=O)=C\C5=CC=C(OC)C=C5)C=C3)=C1	7.318
6	ClC1=CC=C2C(N=CC=C2N/N=C/C3=CC=C(O/C(C(C4=CC=C(Cl)C=C4)=O)=C\C5=C(OC)C=CC(OC)=C5)C=C3)=C1	6.601
7	ClC1=CC=C2C(N=CC=C2N/N=C/C3=CC=C(O/C(C(C4=CC=C(Cl)C=C4)=O)=C\C5=C(OC)C=C(OC)C(OC)=C5)C=C3)=C1	7.043
8	ClC1=CC=C2C(N=CC=C2N/N=C/C3=CC=C(O/C(C(C4=CC=C(Cl)C=C4)=O)=C\C5=CC(OC)=C(OC)C(OC)=C5)C=C3)=C1	7.021
9	ClC1=CC=C2C(N=CC=C2N/N=C/C3=CC=C(O/C(C(C4=CC=C(Cl)C=C4)=O)=C\C5=COC=C5)C=C3)=C1	7.242
10	ClC1=CC=C2C(N=CC=C2N/N=C/C3=CC=C(O/C(C(C4=CC=C(Cl)C=C4)=O)=C\C5=CC=C(F)C=C5F)C=C3)=C1	6.858
11	ClC1=CC=C2C(N=CC=C2N/N=C/C3=CC=C(O/C(C(C4=CC=C(Cl)C=C4)=O)=C\C5=CC=C(Cl)C=C5Cl)C=C3)=C1	6.530
12	ClC1=CC=C2C(N=CC=C2N/N=C/C3=CC=C(O/C(C(C4=CC=C(Cl)C=C4)=O)=C\C5=CC=CC([N+]([O-])=O)=C5)C=C3)=C1	6.892
13	ClC1=CC=C(Cl)C=C1OC2=C(N/C(NC3=CC=C(Cl)C=C3)=N/CCCCNC4=CC=NC5=CC(Cl)=CC=C54)C=CC=C2	6.185
14	ClC1=CC=C2C(N=CC=C2NCC/N=C(NC3=CC=C(OC4=CC=C(OC)C=C4)C=C3)/NC5=CC=CC=C5)=C1	7.377
15	ClC1=CC=C2C(N=CC=C2NCCC/N=C(NC3=CC=C(OC4=CC=C(OC)C=C4)C=C3)\NC5=CC=CC=C5)=C1	6.920
16	ClC1=CC=C2C(N=CC=C2NCCCC/N=C(NC3=CC=C(OC4=CC=C(OC)C=C4)C=C3)\NC5=CC=CC=C5)=C1	6.963
17	ClC1=CC=C2C(N=CC=C2NCC/N=C(NC3=CC=C(OC4=CC=C(OC)C=C4)C=C3)\NC5=CC=CC(Cl)=C5)=C1	7.362
18	ClC1=CC=C2C(N=CC=C2NCCC/N=C(NC3=CC=C(OC4=CC=C(OC)C=C4)C=C3)\NC5=CC=CC(Cl)=C5)=C1	6.433
19	ClC1=CC=C2C(N=CC=C2NCCCC/N=C(NC3=CC=C(OC4=CC=C(OC)C=C4)C=C3)\NC5=CC=CC(Cl)=C5)=C1	7.425
20	ClC1=CC=C(NC(NC2=CC=C(OC3=CC=C(OC)C=C3)C=C2)=NCCNC4=CC=NC5=CC(Cl)=CC=C54)C=C1	6.986
21	ClC1=CC=C(N/C(NC2=CC=C(OC3=CC=C(OC)C=C3)C=C2)=N\CCCNC4=CC=NC5=CC(Cl)=CC=C54)C=C1	6.618
22	ClC1=CC=C(NC(NC2=CC=C(OC3=CC=C(OC)C=C3)C=C2)=NCCCCNC4=CC=NC5=CC(Cl)=CC=C54)C=C1	6.907
23	C/C(C(OC)=O)=C/C1=CC=C(OC2=C(N/C(NCCCNC3=C(C=CC(Cl)=C4)C4=NC=C3)=N/C5=CC=CC=C5)C=CC=C2)C=C1	6.758
24	C/C(C(OC)=O)=C/C1=CC=C(OC2=C(N/C(NCCCCNC3=C(C=CC(Cl)=C4)C4=NC=C3)=N/C5=CC=CC=C5)C=CC=C2)C=C1	6.542
25	C/C(C(OC)=O)=C/C1=CC=C(OC2=C(N/C(NCCCNC3=C(C=CC(Cl)=C4)C4=NC=C3)=N/C5=CC(Cl)=CC=C5)C=CC=C2)C=C1	7.692

**26**	C/C(C(OC)=O)=C/C1=CC=C(OC2=C(N/C(NCCCCNC3=C(C=CC(Cl)=C4)C4=NC=C3)=N/C5=CC(Cl)=CC=C5)C=CC=C2)C=C1	7.505
**27**	C/C(C(OC)=O)=C/C1=CC=C(OC2=C(N/C(NCCNC3=C(C=CC(Cl)=C4)C4=NC=C3)=N/C5=CC=C(Cl)C=C5)C=CC=C2)C=C1	7.288
**28**	C/C(C(OC)=O)=C/C1=CC=C(OC2=C(N/C(NCCCNC3=C(C=CC(Cl)=C4)C4=NC=C3)=N/C5=CC=C(Cl)C=C5)C=CC=C2)C=C1	7.128
**29**	C/C(C(OC)=O)=C/C1=CC=C(OC2=C(N/C(NCCCCNC3=C(C=CC(Cl)=C4)C4=NC=C3)=N/C5=CC=C(Cl)C=C5)C=CC=C2)C=C1	7.014
**30**	ClC1=CC=C(Cl)C=C1OC2=CC=C(N/C(NC3=CC=CC=C3)=N\CCNC4=CC=NC5=CC(Cl)=CC=C54)C=C2	7.404
**31**	ClC1=CC=C(Cl)C=C1OC2=CC=C(N/C(NC3=CC=CC=C3)=N\CCCNC4=CC=NC5=CC(Cl)=CC=C54)C=C2	7.636
**32**	ClC1=CC=C(Cl)C=C1OC2=CC=C(N/C(NC3=CC=CC=C3)=N\CCCCNC4=CC=NC5=CC(Cl)=CC=C54)C=C2	6.512
**33**	ClC1=CC=C(Cl)C=C1OC2=CC=C(N/C(NC3=CC(Cl)=CC=C3)=N\CCNC4=CC=NC5=CC(Cl)=CC=C54)C=C2	7.202
**34**	ClC1=CC=C(Cl)C=C1OC2=CC=C(N/C(NC3=CC(Cl)=CC=C3)=N\CCCNC4=CC=NC5=CC(Cl)=CC=C54)C=C2	7.118
**35**	ClC1=CC=C(Cl)C=C1OC2=CC=C(N/C(NC3=CC(Cl)=CC=C3)=N\CCCCNC4=CC=NC5=CC(Cl)=CC=C54)C=C2	6.900
**36**	ClC1=CC=C(Cl)C=C1OC2=CC=C(N/C(NC3=CC=C(Cl)C=C3)=N\CCNC4=CC=NC5=CC(Cl)=CC=C54)C=C2	7.357
**37**	ClC1=CC=C(Cl)C=C1OC2=CC=C(N/C(NC3=CC=C(Cl)C=C3)=N\CCCCNC4=CC=NC5=CC(Cl)=CC=C54)C=C2	7.537
**38**	ClC1=CC(OC)=CC=C1OC2=CC=C(N/C(NC3=CC=CC=C3)=N\CCCNC4=CC=NC5=CC(Cl)=CC=C54)C=C2	7.436
**39**	ClC1=CC(OC)=CC=C1OC2=CC=C(N/C(NC3=CC=CC=C3)=N/CCCCNC4=CC=NC5=CC(Cl)=CC=C54)C=C2	7.066
**40**	ClC1=CC(OC)=CC=C1OC2=CC=C(N/C(NC3=CC(Cl)=CC=C3)=N/CCNC4=CC=NC5=CC(Cl)=CC=C54)C=C2	7.052
**41**	ClC1=CC(OC)=CC=C1OC2=CC=C(N/C(NC3=CC(Cl)=CC=C3)=N/CCCNC4=CC=NC5=CC(Cl)=CC=C54)C=C2	7.305
**42**	ClC1=CC(OC)=CC=C1OC2=CC=C(N/C(NC3=CC=C(Cl)C=C3)=N/CCNC4=CC=NC5=CC(Cl)=CC=C54)C=C2	7.161
**43**	ClC1=CC(OC)=CC=C1OC2=CC=C(N/C(NC3=CC=C(Cl)C=C3)=N/CCCNC4=CC=NC5=CC(Cl)=CC=C54)C=C2	6.690
**44**	ClC1=CC(OC)=CC=C1OC2=CC=C(N/C(NC3=CC=C(Cl)C=C3)=N/CCCCNC4=CC=NC5=CC(Cl)=CC=C54)C=C2	7.472
**45**	C/C(C(OC)=O)=C/C1=CC=C(OC2=CC=C(N/C(NCCNC3=C(C=CC(Cl)=C4)C4=NC=C3)=N/C5=CC=CC=C5)C=C2)C=C1	7.153
**46**	C/C(C(OC)=O)=C/C1=CC=C(OC2=CC=C(N/C(NCCCNC3=C(C=CC(Cl)=C4)C4=NC=C3)=N/C5=CC=CC=C5)C=C2)C=C1	7.107
**47**	C/C(C(OC)=O)=C/C1=CC=C(OC2=CC=C(N/C(NCCCCNC3=C(C=CC(Cl)=C4)C4=NC=C3)=N/C5=CC=CC=C5)C=C2)C=C1	7.025
**48**	C/C(C(OC)=O)=C/C1=CC=C(OC2=CC=C(N/C(NCCNC3=C(C=CC(Cl)=C4)C4=NC=C3)=N/C5=CC(Cl)=CC=C5)C=C2)C=C1	7.311
**49**	C/C(C(OC)=O)=C/C1=CC=C(OC2=CC=C(N/C(NCCCNC3=C(C=CC(Cl)=C4)C4=NC=C3)=N/C5=CC(Cl)=CC=C5)C=C2)C=C1	7.107
**50**	C/C(C(OC)=O)=C/C1=CC=C(OC2=CC=C(N/C(NCCCCNC3=C(C=CC(Cl)=C4)C4=NC=C3)=N/C5=CC(Cl)=CC=C5)C=C2)C=C1	7.285
**51**	C/C(C(OC)=O)=C/C1=CC=C(OC2=CC=C(N/C(NCCNC3=C(C=CC(Cl)=C4)C4=NC=C3)=N/C5=CC=C(Cl)C=C5)C=C2)C=C1	7.366

**52**	C/C(C(OC)=O)=C/C1=CC=C(OC2=CC=C(N/C(NCCCNC3=C(C=CC(Cl)=C4)C4=NC=C3)=N/C5=CC=C(Cl)C=C5)C=C2)C=C1	7.414
**53**	C/C(C(OC)=O)=C/C1=CC=C(OC2=CC=C(N/C(NCCCCNC3=C(C=CC(Cl)=C4)C4=NC=C3)=N/C5=CC=C(Cl)C=C5)C=C2)C=C1	7.249
**54**	O=C(/C(SC1C2=CC=CC=C2)=C/C3=CC=CC=C3)N1NC4=NC5=CC=CC=C5C(C)=C4	6.000
**55**	O=C(/C(SC1C2=CC=CC=C2)=C/C3=CC=C(Cl)C=C3)N1NC4=NC5=CC=CC=C5C(C)=C4	6.136
**56**	O=C(/C(SC1C2=CC=CC=C2)=C/C3=CC=C(C)C=C3)N1NC4=NC5=CC=CC=C5C(C)=C4	6.000
**57**	O=C(/C(SC1C2=CC=CC=C2)=C/C3=CC=C(OC)C=C3)N1NC4=NC5=CC=CC=C5C(C)=C4	6.000
**58**	O=C(/C(SC1C2=CC=C(Cl)C=C2)=C/C3=CC=CC=C3)N1NC4=NC5=CC=CC=C5C(C)=C4	6.134
**59**	CC1=CC(NN2C(/C(SC2C3=CC=C(Cl)C=C3)=C/C4=CC=C(Cl)C=C4)=O)=NC5=CC=CC=C51	6.106
**60**	O=C(/C(SC1C2=CC=C(Cl)C=C2)=C/C3=CC=C(C)C=C3)N1NC4=NC5=CC=CC=C5C(C)=C4	6.374
**61**	CC1=CC(NN2C(/C(SC2C3=CC=C(Cl)C=C3)=C/C4=CC=C(OC)C=C4)=O)=NC5=CC=CC=C51	6.102
**62**	Cc5ccc(C2S/C(=C\c1ccccc1)C(=O)N2Nc4cc(C)c3ccccc3n4)cc5	5.699
**63**	CC1=CC=C(C=C1)C2S\C(=C/C3=CC=C(Cl)C=C3)C(=O)N2NC4=CC(=C5C=CC=CC5=N4)C	6.250
**64**	CC1=CC=C(C=C1)\C=C2/SC(N(NC3=CC(=C4C=CC=CC4=N3)C)C2=O)C5=CC=C(C)C=C5	5.699
**65**	COC1=CC=C(C=C1)\C=C2/SC(N(NC3=CC(=C4C=CC=CC4=N3)C)C2=O)C5=CC=C(C)C=C5	5.699
**66**	COC1=CC=C(C=C1)C2S\C(=C/C3=CC=CC=C3)C(=O)N2NC4=CC(=C5C=CC=CC5=N4)C	6.000
**67**	COC1=CC=C(C=C1)C2S\C(=C/C3=CC=C(Cl)C=C3)C(=O)N2NC4=CC(=C5C=CC=CC5=N4)C	6.199
**68**	COC1=CC=C(C=C1)C2S\C(=C/C3=CC=C(C)C=C3)C(=O)N2NC4=CC(=C5C=CC=CC5=N4)C	5.699
**69**	COC1=CC=C(C=C1)\C=C2/SC(N(NC3=CC(=C4C=CC=CC4=N3)C)C2=O)C5=CC=C(OC)C=C5	5.699
**70**	CC1=C2C=CC=CC2=NC(=C1)NN3C(S\C(=C/C4=CC=CC=C4)C3=O)C5=CC=CS5	5.699
**71**	CC1=C2C=CC=CC2=NC(=C1)NN3C(S\C(=C/C4=CC=CS4)C3=O)C5=CC=CC=C5	5.523
**72**	COC1=CC2=C(NC3=CC=C(C=C3)C(=O)\C=C\C4=CC=CC=C4)C5=CC=C(Cl)C=C5N=C2C=C1	6.125
**73**	COC1=CC2=C(NC3=CC=C(C=C3)C(=O)\C=C\C4=CC=CC=C4Cl)C5=CC=C(Cl)C=C5N=C2C=C1	5.824
**74**	COC1=CC2=C(NC3=CC=C(C=C3)C(=O)\C=C\C4=CC=CC=C4[N+]([O-])=O)C5=CC=C(Cl)C=C5N=C2C=C1	6.456
**75**	COC1=CC2=C(NC3=CC=C(C=C3)C(=O)\C=C\C4=CC=CC=C4OC)C5=CC=C(Cl)C=C5N=C2C=C1	6.284
**76**	COC1=CC2=C(NC3=CC=C(C=C3)C(=O)\C=C\C4=CC=CC=C4O)C5=CC=C(Cl)C=C5N=C2C=C1	5.347
**77**	COC1=CC2=C(NC3=CC=C(C=C3)C(=O)\C=C\C4=CC=CC(=C4)[N+]([O-])=O)C5=CC=C(Cl)C=C5N=C2C=C1	5.886
**78**	COC1=CC(=CC=C1)\C=C\C(=O)C2=CC=C(NC3=C4C=C(OC)C=CC4=NC5=CC(=CC=C35)Cl)C=C2	5.745
**79**	COC1=CC2=C(NC3=CC=C(C=C3)C(=O)\C=C\C4=CC=C(O)C(=C4)O)C5=CC=C(Cl)C=C5N=C2C=C1	5.699
**80**	COC1=CC2=C(NC3=CC=C(C=C3)C(=O)\C=C\C4=CC=C(OC)C(=C4)OC)C5=CC=C(Cl)C=C5N=C2C=C1	5.398
**81**	COC1=CC2=C(NC3=CC=C(C=C3)C(=O)\C=C\C4=CC=C(Cl)C=C4)C5=CC=C(Cl)C=C5N=C2C=C1	5.886
**82**	COC1=CC2=C(NC3=CC=C(C=C3)C(=O)\C=C\C4=CC=C(C=C4)[N+]([O-])=O)C5=CC=C(Cl)C=C5N=C2C=C1	6.155
**83**	COC1=CC=C(\C=C\C(=O)C2=CC=C(NC3=C4C=C(OC)C=CC4=NC5=CC(=CC=C35)Cl)C=C2)C=C1	6.523

**84**	COC1=CC2=C(NC3=CC=C(C=C3)C(=O)\C=C\C4=CC=C(O)C=C4)C5=CC=C(Cl)C=C5N=C2C=C1	6.097
**85**	COC1=CC2=C(NC3=CC=C(C=C3)C(=O)\C=C\C4=CC=C(C)C=C4)C5=CC=C(Cl)C=C5N=C2C=C1	5.319
**86**	CCC1=CC=C(\C=C\C(=O)C2=CC=C(NC3=C4C=C(OC)C=CC4=NC5=CC(=CC=C35)Cl)C=C2)C=C1	5.620
**87**	COC1=CC2=C(NC3=CC=C(C=C3)C(=O)\C=C\C4=CC=C(C=C4)N(C)C)C5=CC=C(Cl)C=C5N=C2C=C1	5.602
**88**	COC1=CC2=C(NC3=CC=C(C=C3)C(=O)\C=C\C4=CC=C(C=C4)C#N)C5=CC=C(Cl)C=C5N=C2C=C1	5.770
**89**	COC1=CC2=C(NC3=CC=C(C=C3)C(=O)\C=C\C4=CC=C(F)C=C4)C5=CC=C(Cl)C=C5N=C2C=C1	5.569
**90**	CCOC(=O)C1=C(N=C(NCCNC2=C3C=CC(=CC3=NC=C2)Cl)N=C1C)C4=CC=CC=C4	7.664
**91**	CCOC(=O)C1=C(N=C(NCCCNC2=C3C=CC(=CC3=NC=C2)Cl)N=C1C)C4=CC=CC=C4	6.606
**92**	CCOC(=O)C1=C(N=C(NCCCCNC2=C3C=CC(=CC3=NC=C2)Cl)N=C1C)C4=CC=CC=C4	6.694
**93**	CCOC(=O)C1=C(N=C(NCCCCCCCCCCNC2=C3C=CC(=CC3=NC=C2)Cl)N=C1C)C4=CC=CC=C4	5.699
**94**	O=C(OCC)C1=C(C)N=C(NCCCCCCCCCCCCNC2=CC=NC3=CC(Cl)=CC=C32)N=C1C4=CC=CC=C4	6.577
**95**	O=C(OCC)C1=C(C)N=C(NC(C=C2)=CC=C2NC3=CC=NC4=CC(Cl)=CC=C43)N=C1C5=CC=CC=C5	6.191
**96**	O=C(OCC)C1=C(C)N=C(NCCCC(C)CNC2=CC=NC3=CC(Cl)=CC=C32)N=C1C4=CC=CC=C4	6.543
**97**	O=C(OCC)C1=C(C)N=C(NCCCNC2=CC=NC3=CC(Cl)=CC=C32)N=C1C	7.200
**98**	O=C(OCC)C1=C(C)N=C(NCCCCNC2=CC=NC3=CC(Cl)=CC=C32)N=C1C4=CC=CC([N+]([O-])=O)=C4	6.157
**99**	O=C(OCC)C1=C(C)N=C(NCCNC2=CC=NC3=CC(Cl)=CC=C32)N=C1C4=CC=CC=C4[N+]([O-])=O	6.762
**100**	O=C(OCC)C1=C(C)N=C(NCCCNC2=CC=NC3=CC(Cl)=CC=C32)N=C1C4=CC=CC=C4[N+]([O-])=O	5.699
**101**	O=C(OCC)C1=C(C)N=C(NCCCNC2=CC=NC3=CC(Cl)=CC=C32)N=C1C4=CC=C([N+]([O-])=O)C=C4	6.301
**102**	O=C1C2=C(N(NC3=CC=CC=C3)C(N)=C(C#N)C2C4=CC5=CC=CC=C5N=C4Cl)CC(C)(C)C1	7.824
**103**	O=C1C2=C(N(NC3=CC=CC=C3)C(N)=C(C#N)C2C4=CC5=CC(C)=CC=C5N=C4Cl)CC(C)(C)C1	6.824
**104**	O=C1C2=C(N(NC3=CC=CC=C3)C(N)=C(C#N)C2C4=CC5=CC(OC)=CC=C5N=C4Cl)CC(C)(C)C1	6.658
**105**	O=C1C2=C(N(NC3=CC=CC=C3)C(N)=C(C#N)C2C4=CC5=CC(Cl)=CC=C5N=C4Cl)CC(C)(C)C1	6.678
**106**	O=C1C2=C(N(NC3=CC=C(Cl)C=C3)C(N)=C(C#N)C2C4=CC5=CC=CC=C5N=C4Cl)CC(C)(C)C1	6.377
**107**	O=C1C2=C(N(NC3=CC=C(Cl)C=C3)C(N)=C(C#N)C2C4=CC5=CC(OC)=CC=C5N=C4Cl)CC(C)(C)C1	8.097
**108**	O=C1C2=C(N(NC3=CC=C(Cl)C=C3)C(N)=C(C#N)C2C4=CC5=CC(OC)=CC=C5N=C4Cl)CC(C)(C)C1	8.046
**109**	O=C1C2=C(N(NC3=CC=C(C)C=C3)C(N)=C(C#N)C2C4=CC5=CC=CC=C5N=C4Cl)CC(C)(C)C1	7.553
**110**	O=C1C2=C(N(NC3=CC=C(C)C=C3)C(N)=C(C#N)C2C4=CC5=CC(OC)=CC=C5N=C4Cl)CC(C)(C)C1	6.658
**111**	O=C1C2=C(N(NC3=CC=C(C)C=C3)C(N)=C(C#N)C2C4=CC5=CC(Cl)=CC=C5N=C4Cl)CC(C)(C)C1	6.721

**112**	O=C1C2=C(N(NC3=CC=C(F)C=C3)C(N)=C(C#N)C2C4=CC5=CC=CC=C5N=C4Cl)CC(C)(C)C1	7.854
**113**	O=C1C2=C(N(NC3=CC=C(F)C=C3)C(N)=C(C#N)C2C4=CC5=CC(C)=CC=C5N=C4Cl)CC(C)(C)C1	7.569
**114**	O=C1C2=C(N(NC3=CC=C(F)C=C3)C(N)=C(C#N)C2C4=CC5=CC(OC)=CC=C5N=C4Cl)CC(C)(C)C1	7.328
**115**	O=C1C2=C(N(NC3=CC=C(F)C=C3)C(N)=C(C#N)C2C4=CC5=CC(Cl)=CC=C5N=C4Cl)CC(C)(C)C1	6.602
**116**	O=C1C2=C(N(NC3=CC=C(OC)C=C3)C(N)=C(C#N)C2C4=CC5=CC=CC=C5N=C4Cl)CC(C)(C)C1	6.813
**117**	O=C1C2=C(N(NC3=CC=C(OC)C=C3)C(N)=C(C#N)C2C4=CC5=CC(C)=CC=C5N=C4Cl)CC(C)(C)C1	7.602
**118**	O=C1C2=C(N(NC3=CC=C(OC)C=C3)C(N)=C(C#N)C2C4=CC5=CC(OC)=CC=C5N=C4Cl)CC(C)(C)C1	6.268
**119**	O=C1C2=C(N(NC3=CC=C(OC)C=C3)C(N)=C(C#N)C2C4=CC5=CC(Cl)=CC=C5N=C4Cl)CC(C)(C)C1	7.721
**120**	O=C1C2=C(N(NC3=CC=C(Br)C=C3)C(N)=C(C#N)C2C4=CC5=CC=CC=C5N=C4Cl)CC(C)(C)C1	7.585
**121**	O=C1C2=C(N(NC3=CC=C(Br)C=C3)C(N)=C(C#N)C2C4=CC5=CC(C)=CC=C5N=C4Cl)CC(C)(C)C1	7.921
**122**	O=C1C2=C(N(NC3=CC=C(Br)C=C3)C(N)=C(C#N)C2C4=CC5=CC(OC)=CC=C5N=C4Cl)CC(C)(C)C1	7.854
**123**	O=C1C2=C(N(NC3=CC=C(Br)C=C3)C(N)=C(C#N)C2C4=CC5=CC(Cl)=CC=C5N=C4Cl)CC(C)(C)C1	7.097
**124**	CC(N1N=NN=C1C2=NC3=CC=C(OC)C=C3C(CN(C)C)=C2)(C)C	6.000
**125**	CC(N1N=NN=C1C2=NC3=CC=C(OC)C=C3C(CN(CC)CC)=C2)(C)C	5.699
**126**	CC(N1N=NN=C1C2=NC3=CC=C(OC)C=C3C(CN4CCCC4)=C2)(C)C	4.859
**127**	CC(N1N=NN=C1C2=NC3=CC=C(OC)C=C3C(CN4CCCCC4)=C2)(C)C	6.189
**128**	CC(N1N=NN=C1C2=NC3=CC=C(OC)C=C3C(CN(CC4)CCN4C)=C2)(C)C	4.770
**129**	CC(N1N=NN=C1C2=NC3=CC=C(OC)C=C3C(CN4CCOCC4)=C2)(C)C	6.009
**130**	CN(C1=CC=C2N=CC=C(C(N3CCOCC3)C4=NN=NN4)C2=C1)C	5.046
**131**	O=C(C1=CC=C(Br)C=C1)/C=C/C2=CC=CC(NC3=CC=NC4=CC(Cl)=CC=C34)=C2	4.986
**132**	O=C(C1=CC=C(OC)C=C1)/C=C/C2=CC=CC(NC3=CC=NC4=CC(Cl)=CC=C34)=C2	4.977
**133**	O=C(C1=CC(OC)=C(OC)C(OC)=C1)/C=C/C2=CC=CC(NC3=CC=NC4=CC(Cl)=CC=C34)=C2	4.850
**134**	O=C(C1=CC=C(C)C=C1)/C=C/C2=CC=CC(NC3=CC=NC4=CC(Cl)=CC=C34)=C2	4.989
**135**	O=C(C1=CC=CC=C1)/C=C/C2=CC=CC(NC3=CC=NC4=CC(Cl)=CC=C34)=C2	4.989
**136**	ClC1=CC=C2C(NC3=CC(C4CC(C5=CC=C(Cl)C=C5)=NN4C(C)=O)=CC=C3)=CC=NC2=C1	4.580
**137**	ClC1=CC=C2C(NC3=CC(C4CC(C5=CC=C(Br)C=C5)=NN4C(C)=O)=CC=C3)=CC=NC2=C1	4.300
**138**	ClC1=CC=C2C(NC3=CC(C4CC(C5=CC=C(OC)C=C5)=NN4C(C)=O)=CC=C3)=CC=NC2=C1	4.860
**139**	ClC1=CC=C2C(NC3=CC(C4CC(C5=CC(OC)=C(OC)C(OC)=C5)=NN4C(C)=O)=CC=C3)=CC=NC2=C1	4.639
**140**	ClC1=CC=C2C(NC3=CC(C4CC(C5=CC=C(C)C=C5)=NN4C(C)=O)=CC=C3)=CC=NC2=C1	4.757
**141**	ClC1=CC=C2C(NC3=CC(C4CC(C5=CC=CC=C5)=NN4C(C)=O)=CC=C3)=CC=NC2=C1	4.898
**142**	ClC1=CC=C2C(NC3=CC(C4CC(C5=CC=CC=C5)=NN4C(C)=O)=CC=C3)=CC=NC2=C1	4.607
**143**	O=CN1N=C(C2=CC=C(Br)C=C2)CC1C3=CC=CC(NC4=CC=NC5=CC(Cl)=CC=C45)=C3	4.805
**144**	O=CN1N=C(C2=CC=C(OC)C=C2)CC1C3=CC=CC(NC4=CC=NC5=CC(Cl)=CC=C45)=C3	4.792
**145**	O=CN1N=C(C2=CC(OC)=C(OC)C(OC)=C2)CC1C3=CC=CC(NC4=CC=NC5=CC(Cl)=CC=C45)=C3	4.615
**146**	O=CN1N=C(C2=CC=C(C)C=C2)CC1C3=CC=CC(NC4=CC=NC5=CC(Cl)=CC=C45)=C3	4.791

**147**	O=CN1N=C(C2=CC=CC=C2)CC1C3=CC=CC(NC4=CC=NC5=CC(Cl)=CC=C45)=C3	4.753
**148**	ClC1=CC=C(C2=NN(C3=CC=CC=C3)C(C4=CC(NC5=CC=NC6=CC(Cl)=CC=C56)=CC=C4)C2)C=C1	4.931
**149**	BrC1=CC=C(C2=NN(C3=CC=CC=C3)C(C4=CC(NC5=CC=NC6=CC(Cl)=CC=C56)=CC=C4)C2)C=C1	5.257
**150**	ClC1=CC=C2C(NC3=CC=CC(C4CC(C5=CC=C(OC)C=C5)=NN4C6=CC=CC=C6)=C3)=CC=NC2=C1	4.865
**151**	ClC1=CC=C2C(NC3=CC=CC(C4CC(C5=CC(OC)=C(OC)C(OC)=C5)=NN4C6=CC=CC=C6)=C3)=CC=NC2=C1	4.810
**152**	ClC1=CC=C2C(NC3=CC=CC(C4CC(C5=CC=C(C)C=C5)=NN4C6=CC=CC=C6)=C3)=CC=NC2=C1	4.869
**153**	ClC1=CC=C2C(NC3=CC=CC(C4CC(C5=CC=CC=C5)=NN4C6=CC=CC=C6)=C3)=CC=NC2=C1	4.874
**154**	ClC1=CC=C2C(NC3=CC=CC(C4CC(C5=CC=C(Cl)C=C5)=NN4C6=CC=C(Cl)C=C6)=C3)=CC=NC2=C1	4.989
**155**	ClC1=CC=C2C(NC3=CC=CC(C4CC(C5=CC=C(Br)C=C5)=NN4C6=CC=C(Cl)C=C6)=C3)=CC=NC2=C1	4.989
**156**	ClC1=CC=C2C(NC3=CC=CC(C4CC(C5=CC=C(OC)C=C5)=NN4C6=CC=C(Cl)C=C6)=C3)=CC=NC2=C1	4.351
**157**	ClC1=CC=C2C(NC3=CC=CC(C4CC(C5=CC(OC)=C(OC)C(OC)=C5)=NN4C6=CC=C(Cl)C=C6)=C3)=CC=NC2=C1	4.351
**158**	ClC1=CC=C2C(NC3=CC=CC(C4CC(C5=CC=C(C)C=C5)=NN4C6=CC=C(Cl)C=C6)=C3)=CC=NC2=C1	4.864
**159**	ClC1=CC=C2C(NC3=CC=CC(C4CC(C5=CC=CC=C5)=NN4C6=CC=C(Cl)C=C6)=C3)=CC=NC2=C1	4.989
**160**	ClC1=CC=C2C(NC3=CC=CC(C4CC(C5=CC=C(Cl)C=C5)=NN4C6=CC(Cl)=CC(Cl)=C6)=C3)=CC=NC2=C1	4.945
**161**	ClC1=CC=C2C(NC3=CC=CC(C4CC(C5=CC=C(Br)C=C5)=NN4C6=CC(Cl)=CC(Cl)=C6)=C3)=CC=NC2=C1	4.842
**162**	ClC1=CC=C2C(NC3=CC=CC(C4CC(C5=CC=C(OC)C=C5)=NN4C6=CC(Cl)=CC(Cl)=C6)=C3)=CC=NC2=C1	4.983
**163**	ClC1=CC=C2C(NC3=CC=CC(C4CC(C5=CC(OC)=C(OC)C(OC)=C5)=NN4C6=CC(Cl)=CC(Cl)=C6)=C3)=CC=NC2=C1	4.963
**164**	ClC1=CC=C2C(NC3=CC=CC(C4CC(C5=CC=C(C)C=C5)=NN4C6=CC(Cl)=CC(Cl)=C6)=C3)=CC=NC2=C1	4.186
**165**	ClC1=CC=C2C(NC3=CC=CC(C4CC(C5=CC=CC=C5)=NN4C6=CC(Cl)=CC(Cl)=C6)=C3)=CC=NC2=C1	4.901
**166**	C[Si](OC[C@@H](N)C1=CC(C(F)(F)F)=NC2=C(C(F)(F)F)C=CC=C12)(C(C)(C)C)C	6.127
**167**	CCCCCN[C@@H](C1=CC(C(F)(F)F)=NC2=C(C(F)(F)F)C=CC=C12)CO[Si](C)(C(C)(C)C)C	5.301
**168**	CCCCCN[C@@H](C1=CC(C(F)(F)F)=NC2=C(C(F)(F)F)C=CC=C12)CO	6.000
**169**	CCCCCN[C@H](C1=CC(C(F)(F)F)=NC2=C(C(F)(F)F)C=CC=C12)CO	5.301
**170**	CCCCCCCN[C@@H](C1=CC(C(F)(F)F)=NC2=C(C(F)(F)F)C=CC=C12)CO	5.523
**171**	CCCCCCCN[C@H](C1=CC(C(F)(F)F)=NC2=C(C(F)(F)F)C=CC=C12)CO	5.699
**172**	CCCCCOC[C@@H](N)C1=CC(C(F)(F)F)=NC2=C(C(F)(F)F)C=CC=C12	5.398
**173**	CCCCCOC[C@H](N)C1=CC(C(F)(F)F)=NC2=C(C(F)(F)F)C=CC=C12	6.000
**174**	O=C(C1=CC=C(C)C=C1)/C=C/C2=C/C(C(C(C)=C2)=O)=C\NCCNC3=CC=NC4=CC(Cl)=CC=C34	10.699
**175**	O=C(C1=CC=C(OC)C=C1)/C=C/C2=C/C(C(C(C)=C2)=O)=C\NCCNC3=CC=NC4=CC(Cl)=CC=C34	10.482
**176**	FC1=CC=C(C(/C=C/C2=C/C(C(C(C)=C2)=O)=C\NCCNC3=CC=NC4=CC(Cl)=CC=C34)=O)C=C1	10.658

**177**	ClC1=CC=C(C(/C=C/C2=C/C(C(C(C)=C2)=O)=C\NCCNC3=CC=NC4=CC(Cl)=CC=C34)=O)C=C1	10.357
**178**	BrC1=CC=C(C(/C=C/C2=C/C(C(C(C)=C2)=O)=C\NCCNC3=CC=NC4=CC(Cl)=CC=C34)=O)C=C1	10.469
**179**	O=C(C1=CC(OC)=C(OC)C(OC)=C1)/C=C/C2=C/C(C(C(C)=C2)=O)=C\NCCNC3=CC=NC4=CC(Cl)=CC=C34	10.721
**180**	O=C(C1=CC=CS1)/C=C/C2=C/C(C(C(C)=C2)=O)=C\NCCNC3=CC=NC4=CC(Cl)=CC=C34	10.678
**181**	O=C(C1=CC=CC=C1)/C=C/C2=C/C(C(C(C(C)CC)=C2)=O)=C\NCCNC3=CC=NC4=CC(Cl)=CC=C34	10.569
**182**	O=C(C1=CC=C(O)C=C1)/C=C/C2=C/C(C(C(C(C)CC)=C2)=O)=C\NCCNC3=CC=NC4=CC(Cl)=CC=C34	10.377
**183**	O=C(C1=CC=C(OC)C=C1)/C=C/C2=C/C(C(C(C(C)CC)=C2)=O)=C\NCCNC3=CC=NC4=CC(Cl)=CC=C34	10.469
**184**	O=C(C1=CC=C(Cl)C=C1)/C=C/C2=C/C(C(C(C(C)CC)=C2)=O)=C\NCCNC3=CC=NC4=CC(Cl)=CC=C34	10.482
**185**	O=C(C1=CC=CO1)/C=C/C2=C/C(C(C(C(C)CC)=C2)=O)=C\NCCNC3=CC=NC4=CC(Cl)=CC=C34	10.854
**186**	O=C(C1=CC=CS1)/C=C/C2=C/C(C(C(C(C)CC)=C2)=O)=C\NCCNC3=CC=NC4=CC(Cl)=CC=C34	10.745
**187**	O=C(C1=CC=C(F)C=C1)/C=C/C2=C/C(C(C(C(C)(C)C)=C2)=O)=C\NCCNC3=CC=NC4=CC(Cl)=CC=C34	10.854
**188**	O=C(C1=CC=C(Cl)C=C1)/C=C/C2=C/C(C(C(C(C)(C)C)=C2)=O)=C\NCCNC3=CC=NC4=CC(Cl)=CC=C34	10.284
**189**	O=C(C1=CC=C(Br)C=C1)/C=C/C2=C/C(C(C(C(C)(C)C)=C2)=O)=C\NCCNC3=CC=NC4=CC(Cl)=CC=C34	11.097
**190**	O=C(C1=CC=C(O)C=C1)/C=C/C2=C/C(C(C(C(C)CC)=C2)=O)=C\NCCCNC3=CC=NC4=CC(Cl)=CC=C34	10.108
**191**	O=C(C1=CC=C(OC)C=C1)/C=C/C2=C/C(C(C(C(C)CC)=C2)=O)=C\NCCCNC3=CC=NC4=CC(Cl)=CC=C34	10.125
**192**	O=C(C1=CC=C(Cl)C=C1)/C=C/C2=C/C(C(C(C(C)CC)=C2)=O)=C\NCCCNC3=CC=NC4=CC(Cl)=CC=C34	10.125
**193**	O=C(C1=CC=C(OC)C=C1)/C=C/C2=C/C(C(C(C)=C2)=O)=C\NC3=CC=C(NC4=CC=NC5=CC(Cl)=CC=C45)C=C3	9.437
**194**	O=C(C1=CC=C(F)C=C1)/C=C/C2=C/C(C(C(C)=C2)=O)=C\NC3=CC=C(NC4=CC=NC5=CC(Cl)=CC=C45)C=C3	9.523
**195**	O=C(C1=CC=CO1)/C=C/C2=C/C(C(C(C(C)CC)=C2)=O)=C\NC3=CC=C(NC4=CC=NC5=CC(Cl)=CC=C45)C=C3	9.836
**196**	ClC1=CC=C2C(NC3=CC=C(N/C=C4C=C(/C=C/C(C5=CC=C(Cl)C=C5)=O)C=C(C(C)(C)C)C\4=O)C=C3)=CC=NC2=C1	9.193
**197**	O=C(C1=C2C3=CC(Cl)=CC=C3N=C1NCCN4CCCCC4)C5=C2C=CC=C5	2.766
**198**	ClC1=CC=C2C3=C(/C(C4=C3C=CC=C4)=N/O)C(NCCN5CCCCC5)=NC2=C1	6.276
**199**	ClC1=CC2=NC3=C(C(OCCN(CC)CC)=C2C=C1)N(CCN(CC)CC)C4=C3C=C(Cl)C=C4	6.801
**200**	ClC1=CC=C2C(NCCN(CC)CC)=C(N(CCN3CCCC3)C4=C5C=CC=C4)C5=NC2=C1	6.183
**201**	ClC1=CC=C2C(NCCN3CCCC3)=C(N(CCN4CCCC4)C5=C6C=CC=C5)C6=NC2=C1	6.330
**202**	ClC1=CC=C2C(NCCCN(CC)CC)=C(N(CCN3CCCC3)C4=C5C=CC=C4)C5=NC2=C1	6.319
**203**	ClC1=CC=C2C(NCCCN3CCCCC3)=C(N(CCN4CCCC4)C5=C6C=CC=C5)C6=NC2=C1	6.272
**204**	ClC1=CC=C2C(NCCCN3CCOCC3)=C(N(CCN4CCCC4)C5=C6C=CC=C5)C6=NC2=C1	6.329
**205**	ClC1=CC=C2C(NC(C)CCCN(CC)CC)=C(N(CCN3CCCC3)C4=C5C=CC=C4)C5=NC2=C1	6.611
**206**	ClC1=CC(C2=NC3=CC(Cl)=CC=C3C(NCCN(CC)CC)=C2N4CCN5CCCC5)=C4C=C1	6.611
**207**	ClC1=CC(C2=NC3=CC(Cl)=CC=C3C(NCCN4CCCC4)=C2N5CCN6CCCC6)=C5C=C1	6.426
**208**	ClC1=CC(C2=NC3=CC(Cl)=CC=C3C(NCCCN(CC)CC)=C2N4CCN5CCCC5)=C4C=C1	6.407

**209**	ClC1=CC(C2=NC3=CC(Cl)=CC=C3C(NCCCN4CCCCC4)=C2N5CCN6CCCC6)=C5C=C1	6.181
**210**	ClC1=CC(C2=NC3=CC(Cl)=CC=C3C(NCCCN4CCOCC4)=C2N5CCN6CCCC6)=C5C=C1	6.390
**211**	ClC1=CC(C2=NC3=CC(Cl)=CC=C3C(NC(C)CCCN(CC)CC)=C2N4CCN5CCCC5)=C4C=C1	6.341
**212**	ClC1=CC=C2C(NCCCN(CC)CC)=C(N(CCN3CCOCC3)C4=C5C=CC=C4)C5=NC2=C1	6.495
**213**	ClC1=CC(C2=NC3=CC(Cl)=CC=C3C(NCCCN(CC)CC)=C2N4CCN5CCOCC5)=C4C=C1	6.701
**214**	ClC1=CC(C2=NC3=CC(Cl)=CC=C3C(NCCN(CC)CC)=C2N4CCN5CCOCC5)=C4C=C1	6.290
**215**	ClC1=CC(C2=NC3=CC(Cl)=CC=C3C(NCCN(CC)CC)=C2N4CCN(CC)CC)=C4C=C1	6.401
**216**	O=C(OCC)CC(O)C1=C2N=C(C3=CC=CC=C3)C=CC2=CC=C1	4.346
**217**	O=C(OCC)CC(O)C1=C2N=C(C3=CC=CC=C3)C=C(C)C2=CC=C1	4.465
**218**	O=C(OCC)CC(C1=C2N=C(C3=CC=CC=C3F)C=CC2=CC=C1)O	4.327
**219**	O=C(OCC)CC(C1=C2N=C(C3=CC=C(C)C(Cl)=C3)C=CC2=CC=C1)O	4.567
**220**	O=C(OCC)CC(O)C1=C2N=C(C3=CC=C([N+]([O-])=O)C=C3)C=CC2=CC=C1	4.434
**221**	O=C(N1C2=CC(C(F)(F)F)=CC=C2)C=CC3=C1C4=CC(C5=CN=C(N6CCCCC6)C=C5)=CC=C4N=C3	5.301
**222**	O=C(N1C2=CC(C(F)(F)F)=CC=C2)C=CC3=C1C4=CC(C5=CN=CC(N)=C5)=CC=C4N=C3	6.810
**223**	O=C(N1C2=CC(C(F)(F)F)=CC=C2)C=CC3=C1C4=CC(C5=CN=CC=C5)=CC=C4N=C3	7.143
**224**	O=C(N1C2=CC(C(F)(F)F)=CC=C2)C=CC3=C1C4=CC(C5=CC=NC=C5)=CC=C4N=C3	7.036
**225**	O=C(N1C2=CC(C(F)(F)F)=CC=C2)C=CC3=C1C4=CC(C5=CNC(C=C5)=O)=CC=C4N=C3	6.893
**226**	O=C(N1C2=CC(C(F)(F)F)=CC=C2)C=CC3=C1C4=CC(C5=CN=C(C(F)(F)F)C=C5)=CC=C4N=C3	6.810
**227**	O=C(N1C2=CC(C(F)(F)F)=CC=C2)C=CC3=C1C4=CC(C5=CN=CC(C)=C5)=CC=C4N=C3	5.699
**228**	O=C(N1C2=CC(C(F)(F)F)=CC=C2)C=CC3=C1C4=CC(C5=CN=C(N(C)C)N=C5)=CC=C4N=C3	5.699
**229**	O=C(N1C2=CC(C(F)(F)F)=CC=C2)C=CC3=C1C4=CC(C5=CN=C(N)N=C5C)=CC=C4N=C3	6.126
**230**	O=C(N1C2=CC(C(F)(F)F)=CC=C2)C=CC3=C1C4=CC(C5=CN=CN=C5)=CC=C4N=C3	6.445
**231**	O=C(N1C2=CC(C(F)(F)F)=CC=C2)C=CC3=C1C4=CC(C5=CNN=C5)=CC=C4N=C3	7.097
**232**	O=C(N1C2=CC(C(F)(F)F)=CC=C2)C=CC3=C1C4=CC(C5=CSC=C5)=CC=C4N=C3	6.544
**233**	O=C(N1C2=CC(C(F)(F)F)=CC=C2)C=CC3=C1C4=CC(C5=COC=C5)=CC=C4N=C3	6.487
**234**	O=C(N1C2=CC(C(F)(F)F)=CC=C2)C=CC3=C1C4=CC(C5=CN=C(C=CC=C6)C6=C5)=CC=C4N=C3	5.991
**235**	O=C(N1C2=CC(C(F)(F)F)=CC=C2)C=CC3=C1C4=CC(C5=CC=C(NC=C6)C6=C5)=CC=C4N=C3	6.830
**236**	O=C(N1C2=CC(C(F)(F)F)=CC=C2)C=CC3=C1C4=CC(C5=CC=C(SC=C6)C6=C5)=CC=C4N=C3	6.000
**237**	O=C(N1C2=CC(C(F)(F)F)=CC=C2)C=CC3=C1C4=CC(C5=CC=CC=C5)=CC=C4N=C3	6.640
**238**	O=C(N1C2=CC(C(F)(F)F)=CC=C2)C=CC3=C1C4=CC(C5=CC=C(OC)C=C5)=CC=C4N=C3	5.636
**239**	O=C(N1C2=CC(C(F)(F)F)=CC=C2)C=CC3=C1C4=CC(C5=CC=C(S(=O)(C)=O)C=C5)=CC=C4N=C3	6.535
**240**	O=C(N1C2=CC(C(F)(F)F)=CC=C2)C=CC3=C1C4=CC(C5=CC=CC(C(C)=O)=C5)=CC=C4N=C3	6.407
**241**	O=C(N1C2=CC(C(F)(F)F)=CC=C2)C=CC3=C1C4=CC(C5=CC=C(C6=NN=NN6)C=C5)=CC=C4N=C3	4.728
**242**	O=C(N1C2=CC(C(F)(F)F)=CC=C2)C=CC3=C1C4=CC(C5=CC=C(OCC6)C6=C5)=CC=C4N=C3	5.699
**243**	O=C(N1C2=CC(C(F)(F)F)=CC=C2)C=CC3=C1C4=CC(C5=CC=C(OCO6)C6=C5)=CC=C4N=C3	6.133
**244**	O=C(N1C2=C(OC)C=CC=C2)C=CC3=C1C4=CC(C5=CN=C(N)C=C5)=CC=C4N=C3	7.161
**245**	O=C(N1C2=CC(OC)=CC=C2)C=CC3=C1C4=CC(C5=CN=C(N)C=C5)=CC=C4N=C3	7.201
**246**	O=C(N1C2=CC(C(C)C)=CC=C2)C=CC3=C1C4=CC(C5=CN=C(N)C=C5)=CC=C4N=C3	7.009

**247**	O=C(N(C1=O)C2=C(C=CC=C3)C3=NC4=CC=CC=C42)C5=C6C1=CC=C(C(N(CCCC)C7=O)=O)C6=C7C=C5	5.398
**248**	O=C(N(C1=O)C2=C(C=CC=C3)C3=NC4=CC=CC=C42)C5=C6C1=CC=C(C(N(CCN(C)C)C7=O)=O)C6=C7C=C5	6.264
**249**	O=C(N(C1=O)C2=C(C=CC=C3)C3=NC4=CC=CC=C42)C5=C6C1=CC=C(C(N(CCCN7C=CN=C7)C8=O)=O)C6=C8C=C5	6.108
**250**	O=C(N(C1=O)C2=C(C=C(OC)C=C3)C3=NC4=CC(Cl)=CC=C42)C5=C6C1=CC=C(C(N(CCN(C)C)C7=O)=O)C6=C7C=C5	6.378
**251**	O=C(N(CCNC1=C(C=C(OC)C=C2)C2=NC3=CC(Cl)=CC=C31)C4=O)C5=C6C4=CC=C(C(N(CCCC)C7=O)=O)C6=C7C=C5	8.438
**252**	O=C(N(CCNC1=C(C=C(OC)C=C2)C2=NC3=CC(Cl)=CC=C31)C4=O)C5=C6C4=CC=C(C(N(CCN(C)C)C7=O)=O)C6=C7C=C5	8.364
**253**	O=C(N(CCCNC1=C(C=CC(Cl)=C2)C2=NC3=C1C=C(OC)C=C3)C4=O)C5=C6C4=CC=C(C(N(CCCC)C7=O)=O)C6=C7C=C5	8.410
**254**	O=C(N(CCCN1C=CN=C1)C2=O)C3=C4C2=CC=C(C(N(CCCN5C=CN=C5)C6=O)=O)C4=C6C=C3	6.585
**255**	ClC1=CC=C(C(NCCCC)=C(C=C(OC)C=C2)C2=N3)C3=C1	7.578
**256**	FC(C1=CC=C2C(N=CC=C2NCCN3CCN(CC3)C)=C1)(F)F	7.600
**257**	ClC1=CC=C2C(N=CC=C2N[C@@H](C)CN3CCN(CC3)C)=C1	7.773
**258**	FC(C1=CC=C2C(N=CC=C2N[C@@H](C)CN3CCN(CC3)C)=C1)(F)F	7.036
**259**	ClC1=CC=C2C(N=CC=C2N[C@@H](C(C)C)CN3CCN(CC3)C)=C1	7.819
**260**	FC(C1=CC=C2C(N=CC=C2N[C@@H](C(C)C)CN3CCN(CC3)C)=C1)(F)F	6.155
**261**	ClC1=CC=C2C(N=CC=C2N[C@@H](CC(C)C)CN3CCN(CC3)C)=C1	8.056
**262**	ClC1=CC=C2C(N=CC=C2N[C@H](CC(C)C)CN3CCN(CC3)C)=C1	7.963
**263**	ClC1=CC=C2C(N=CC=C2NC(CC(C)C)CN3CCN(CC3)C)=C1	8.078
**264**	FC(C1=CC=C2C(N=CC=C2N[C@@H](CC(C)C)CN3CCN(CC3)C)=C1)(F)F	7.945
**265**	ClC1=CC=C2C(N=CC=C2N[C@@H]([C@@H](C)CC)CN3CCN(CC3)C)=C1	7.833
**266**	FC(C1=CC=C2C(N=CC=C2N[C@@H]([C@@H](C)CC)CN3CCN(CC3)C)=C1)(F)F	6.203
**267**	ClC1=CC=C2C(N=CC=C2N[C@@H](CC3=CC=CC=C3)CN4CCN(CC4)C)=C1	7.988
**268**	FC(C1=CC=C2C(N=CC=C2N[C@@H](CC3=CC=CC=C3)CN4CCN(CC4)C)=C1)(F)F	8.276
**269**	FC(C1=CC=C2C(N=CC=C2NCCCN3CCN(CC3)C)=C1)(F)F	7.090
**270**	ClC1=CC=C2C(N=CC=C2NC(CC3=CC=CC=C3)CCN4CCN(CC4)C)=C1	8.298
**271**	CN(CCCNCC1=CC=C(C2=CC(C3=CC=C(CNCCCN(C)C)C=C3)=NC4=CC=CC=C24)C=C1)C	6.328
**272**	CN(CC1)CCN1CCCCNCC2=CC=C(C3=NC4=CC=CC=C4C(C5=CC=C(CNCCCCN6CCN(CC6)C)C=C5)=C3)C=C2	6.161
**273**	CN1CCN(CCCNCC2=CC=C(C3=NC4=CC=CC=C4C(C5=CC=C(CNCCCN6CCN(CC6)C)C=C5)=C3)C=C2)CC1	6.328
**274**	C1(C2=CC=C(CNCCCN3CCOCC3)C=C2)=NC4=CC=CC=C4C(C5=CC=C(CNCCCN6CCOCC6)C=C5)=C1	7.004
**275**	C1(C2=CC=C(CNCCN3CCOCC3)C=C2)=NC4=CC=CC=C4C(C5=CC=C(CNCCN6CCOCC6)C=C5)=C1	5.873
**276**	CN(CCCCNCC1=CC=C(C2=CC(C3=CC=C(CNCCCCN(C)C)C=C3)=NC4=CC=C(OC)C=C24)C=C1)C	5.510
**277**	CN(CCCNCC1=CC=C(C2=CC(C3=CC=C(CNCCCN(C)C)C=C3)=NC4=CC=C(OC)C=C24)C=C1)C	5.577
**278**	COC1=CC=C2N=C(C3=CC=C(CNCCCCN4CCN(CC4)C)C=C3)C=C(C5=CC=C(CNCCCCN6CCN(CC6)C)C=C5)C2=C1	5.587
**279**	COC1=CC=C2N=C(C3=CC=C(CNCCCN4CCN(CC4)C)C=C3)C=C(C5=CC=C(CNCCCN6CCN(CC6)C)C=C5)C2=C1	5.745

**280**	CN(CCCNCC1=CC=C(C2=CC(C3=CC=C(CNCCCN(C)C)C=C3)=NC4=CC(OC)=CC=C24)C=C1)C	5.498
**281**	COC1=CC=C2C(C3=CC=C(CNCCCCN4CCN(CC4)C)C=C3)=CC(C5=CC=C(CNCCCCN6CCN(CC6)C)C=C5)=NC2=C1	5.618
**282**	COC1=CC=C2C(C3=CC=C(CNCCCN4CCN(CC4)C)C=C3)=CC(C5=CC=C(CNCCCN6CCN(CC6)C)C=C5)=NC2=C1	5.363
**283**	CN(CCCNCC1=CC(C2=CC(C3=CC=CC(CNCCCN(C)C)=C3)=NC4=CC=CC=C24)=CC=C1)C	5.842
**284**	CN(CC1)CCN1CCCNCC2=CC(C3=CC(C4=CC=CC(CNCCCN5CCN(CC5)C)=C4)=NC6=CC=CC=C36)=CC=C2	5.710
**285**	C1(C2=CC=CC(CNCCN3CCOCC3)=C2)=NC4=CC=CC=C4C(C5=CC=CC(CNCCN6CCOCC6)=C5)=C1	6.638
**286**	ClC1=CC2=NC3=CC=C(OC)C=C3C(NCN)=C2C=C1	7.377
**287**	ClC1=CC2=NC3=CC=C(OC)C=C3C(NCCN)=C2C=C1	7.174
**288**	ClC1=CC2=NC3=CC=C(OC)C=C3C(NCCCN)=C2C=C1	6.886
**289**	NCNC1=C2C(C=CC=C2)=NC3=CC=CC=C31	6.032
**290**	NCCNC1=C2C(C=CC=C2)=NC3=CC=CC=C31	5.955
**291**	NCCCNC1=C2C(C=CC=C2)=NC3=CC=CC=C31	5.947
**292**	ClC1=CC2=NC3=CC=C(OC)C=C3C(NCN4CCNCC4)=C2C=C1	7.699
**293**	ClC1=CC2=NC3=CC=C(OC)C=C3C(NCCN4CCNCC4)=C2C=C1	7.222
**294**	ClC1=CC2=NC3=CC=C(OC)C=C3C(NCCCN4CCNCC4)=C2C=C1	6.854
**295**	C12=CC=CC=C1C(NCN3CCNCC3)=C4C(C=CC=C4)=N2	6.076
**296**	C12=CC=CC=C1C(NCCN3CCNCC3)=C4C(C=CC=C4)=N2	5.770
**297**	C12=CC=CC=C1C(NCCCN3CCNCC3)=C4C(C=CC=C4)=N2	5.959
**298**	ClC1=CC2=NC3=CC=C(OC)C=C3C(NCN4CCOCC4)=C2C=C1	6.672
**299**	ClC1=CC2=NC3=CC=C(OC)C=C3C(NCCN4CCOCC4)=C2C=C1	8.000
**300**	ClC1=CC2=NC3=CC=C(OC)C=C3C(NCCCN4CCOCC4)=C2C=C1	8.046
**301**	O=C(C[C@@H](N)CC1=CNC=N1)NCNC2=C3C(C=C(Cl)C=C3)=NC4=CC=C(OC)C=C42	6.575
**302**	O=C(C[C@@H](N)CC1=CNC=N1)NCCNC2=C3C(C=C(Cl)C=C3)=NC4=CC=C(OC)C=C42	6.650
**303**	O=C(C[C@@H](N)CC1=CNC=N1)NCCCNC2=C3C(C=C(Cl)C=C3)=NC4=CC=C(OC)C=C42	6.606
**304**	O=C(C[C@@H](N)CC1=CNC=N1)NCCNC2=C3C(C=CC=C3)=NC4=CC=CC=C42	5.833
**305**	O=C(C[C@@H](N)CC1=CNC=N1)NCCCNC2=C3C(C=CC=C3)=NC4=CC=CC=C42	5.699
**306**	O=C([C@@H]1CCCN1)NCNC2=C3C(C=C(Cl)C=C3)=NC4=CC=C(OC)C=C42	7.377
**307**	O=C([C@@H]1CCCN1)NCCNC2=C3C(C=C(Cl)C=C3)=NC4=CC=C(OC)C=C42	6.682
**308**	O=C([C@@H]1CCCN1)NCCCNC2=C3C(C=C(Cl)C=C3)=NC4=CC=C(OC)C=C42	6.420
**309**	O=C([C@@H]1CCCN1)NCNC2=C3C(C=CC=C3)=NC4=CC=CC=C42	6.315
**310**	O=C([C@@H]1CCCN1)NCCNC2=C3C(C=CC=C3)=NC4=CC=CC=C42	5.708
**311**	O=C([C@@H]1CCCN1)NCCCNC2=C3C(C=CC=C3)=NC4=CC=CC=C42	5.854
**312**	O=C(NC1CCCCC1)C(OC(C2=CC=CC=C2)=O)C3=C4C(C=CC=C4)=NC=C3	5.699
**313**	O=C(NC1CCCCC1)C(OC(C2=CC=C(C(C)(C)C)C=C2)=O)C3=C4C(C=CC=C4)=NC=C3	5.523
**314**	O=C(NC1CCCCC1)C(OC(C2=CC=C(C)C=C2)=O)C3=C4C(C=CC=C4)=NC=C3	5.523
**315**	O=C(NC1CCCCC1)C(OC(C2=CC=C(Cl)C=C2)=O)C3=C4C(C=CC=C4)=NC=C3	5.301
**316**	O=C(NC1CCCCC1)C(OC(C2=CC=CC(Cl)=C2)=O)C3=C4C(C=CC=C4)=NC=C3	5.222
**317**	O=C(NC1CCCCC1)C(OC(C2=CC=C(F)C=C2)=O)C3=C4C(C=CC=C4)=NC=C3	5.155
**318**	O=C(NC1CCCCC1)C(OC(C2=CC=C(Br)C=C2)=O)C3=C4C(C=CC=C4)=NC=C3	5.222
**319**	O=C(NC1CCCCC1)C(OC(C2=CC=C(I)C=C2)=O)C3=C4C(C=CC=C4)=NC=C3	5.523
**320**	O=C(NC1CCCCC1)C(OC(C2=CC=C(C#N)C=C2)=O)C3=C4C(C=CC=C4)=NC=C3	5.398
**321**	O=C(NC1CCCCC1)C(OC(C2=CC=CC3=C2C=CC=C3)=O)C4=C5C(C=CC=C5)=NC=C4	5.432
**322**	O=C(NC(C)(C)C)C(OC(C1=CC=CC=C1)=O)C2=C3C(C=CC=C3)=NC=C2	5.398

**323**	O=C(NC(C)(C)C)C(OC(C1=CC=C(C(C)(C)C)C=C1)=O)C2=C3C(C=CC=C3)=NC=C2	5.523
**324**	O=C(NC(C)(C)C)C(OC(C1=CC=C(Cl)C=C1)=O)C2=C3C(C=CC=C3)=NC=C2	5.222
**325**	O=C(NC(C)(C)C)C(OC(C1=CC=CC(Cl)=C1)=O)C2=C3C(C=CC=C3)=NC=C2	5.222
**326**	O=C(NC(C)(C)C)C(OC(C1=CC=C(F)C=C1)=O)C2=C3C(C=CC=C3)=NC=C2	5.301
**327**	O=C(NC(C)(C)C)C(OC(C1=CC=C(Br)C=C1)=O)C2=C3C(C=CC=C3)=NC=C2	5.301
**328**	O=C(NC(C)(C)C)C(OC(C1=CC=C(C#N)C=C1)=O)C2=C3C(C=CC=C3)=NC=C2	5.523
**329**	O=C(NC(C)(C)C)C(OC(C1=CC=CC2=C1C=CC=C2)=O)C3=C4C(C=CC=C4)=NC=C3	5.155
**330**	O=C(NC1=C(C)C=CC=C1C)C(OC(C2=CC=CC=C2)=O)C3=C4C(C=CC=C4)=NC=C3	5.398
**331**	O=C(NC1=C(C)C=CC=C1C)C(OC(C2=CC=C(C(C)(C)C)C=C2)=O)C3=C4C(C=CC=C4)=NC=C3	6.000
**332**	O=C(NC1=C(C)C=CC=C1C)C(OC(C2=CC=C(Cl)C=C2)=O)C3=C4C(C=CC=C4)=NC=C3	5.398
**333**	O=C(NC1=C(C)C=CC=C1C)C(OC(C2=CC=CC3=C2C=CC=C3)=O)C4=C5C(C=CC=C5)=NC=C4	6.000
**334**	O=C(/C=C/C(NC1=CC=CC(F)=C1)=O)NCCCC(C)NC2=CC(OC)=CC3=C2N=CC=C3	4.861
**335**	O=C(/C=C/C(NC1=CC=C(F)C=C1)=O)NCCCC(C)NC2=CC(OC)=CC3=C2N=CC=C3	5.111
**336**	O=C(/C=C/C(NC1=CC=CC(C(F)(F)F)=C1)=O)NCCCC(C)NC2=CC(OC)=CC3=C2N=CC=C3	4.857
**337**	ClC1=CC2=C(C(NCCCCNC(/C=C/C(NC3=CC=CC(F)=C3)=O)=O)=C1)N=CC=C2	6.842
**338**	ClC1=CC2=C(C(NCCCCNC(/C=C/C(NC3=CC=C(F)C=C3)=O)=O)=C1)N=CC=C2	7.174
**339**	ClC1=CC2=C(C(NCCCCNC(/C=C/C(NC3=CC=CC(Cl)=C3)=O)=O)=C1)N=CC=C2	7.081
**340**	ClC1=CC2=C(C(NCCCCNC(/C=C/C(NC3=CC=C(Cl)C=C3)=O)=O)=C1)N=CC=C2	7.456
**341**	CC1=CC=C(C2CC(C3=CC=CS3)=NN2C4=NC5=CC=CC=C5C(C)=C4)C=C1	5.301
**342**	COC1=CC=C(C2CC(C3=CC=CS3)=NN2C4=NC5=CC=CC=C5C(C)=C4)C=C1	5.222
**343**	ClC1=CC=C(C2CC(C3=CC=CS3)=NN2C4=NC5=CC=CC=C5C(C)=C4)C=C1	5.301
**344**	CC1=CC(N2N=C(C3=CC=CS3)CC2C4=CC=CS4)=NC5=CC=CC=C15	4.921
**345**	NC1=NN(C2=NC3=CC=CC=C3C(C)=C2)C4=NC(C5=CC=C(C)C=C5)=CC(C6=CC=C(OC)C=C6)=C41	1.822
**346**	NC1=NN(C2=NC3=CC=CC=C3C(C)=C2)C4=NC(C5=CC=CC=C5)=CC(C6=CC=C(Cl)C=C6)=C41	2.322
**347**	NC1=NN(C2=NC3=CC=CC=C3C(C)=C2)C4=NC(C5=CC=C(F)C=C5)=CC(C6=CC=C(Cl)C=C6)=C41	2.441
**348**	NC1=NN(C2=NC3=CC=CC=C3C(C)=C2)C4=NC(C5=CC=C(OC)C=C5)=CC(C6=CC=C(Cl)C=C6)=C41	2.394
**349**	NC1=NN(C2=NC3=CC=CC=C3C(C)=C2)C4=NC(C5=CC=C(C)C=C5)=CC(C6=CC=C(Cl)C=C6)=C41	1.949

Table S6. Experimental and predicted activities for the 2D-QSAR model.

Training Set			
**Molecule**	**pIC_{50} experimental**	**pIC_{50} predicted**	**Residual**
**15**	6.920	6.680	0.24
**16**	6.963	6.646	0.32
**17**	7.362	7.026	0.34
**20**	6.986	7.026	-0.04
**21**	6.618	6.992	-0.37
**22**	6.907	6.957	-0.05
**23**	6.758	6.783	-0.03
**24**	6.542	6.745	-0.20
**27**	7.288	7.130	0.16
**31**	7.636	7.290	0.35
**33**	7.202	7.638	-0.44
**34**	7.118	7.603	-0.48
**36**	7.357	7.638	-0.28
**38**	7.436	6.992	0.44
**39**	7.066	6.957	0.11
**40**	7.052	7.338	-0.29
**41**	7.305	7.304	0.00
**42**	7.161	7.338	-0.18
**46**	7.107	6.783	0.32
**47**	7.025	6.745	0.28
**49**	7.107	7.095	0.01
**51**	7.366	7.130	0.24
**52**	7.414	7.095	0.32
**53**	7.249	7.057	0.19
**54**	6.000	5.673	0.33
**55**	6.136	5.985	0.15
**56**	6.000	5.684	0.32
**57**	6.000	5.685	0.31
**58**	6.134	5.985	0.15
**59**	6.106	6.298	-0.19
**61**	6.102	5.998	0.10
**63**	6.250	6.023	0.23
**64**	5.699	5.722	-0.02
**65**	5.699	5.722	-0.02
**66**	6.000	5.711	0.29
**68**	5.699	5.722	-0.02
**69**	5.699	5.723	-0.02
**70**	5.699	5.884	-0.18
**71**	5.523	5.884	-0.36
**73**	5.824	6.063	-0.24
**74**	6.456	6.065	0.39
**77**	5.886	6.065	-0.18

**78**	5.745	5.764	-0.02
**81**	5.886	6.063	-0.18
**82**	6.155	6.065	0.09
**85**	5.319	5.762	-0.44
**86**	5.620	5.724	-0.10
**89**	5.569	5.799	-0.23
**91**	6.606	6.713	-0.11
**92**	6.694	6.680	0.01
**96**	6.543	6.742	-0.20
**97**	7.200	6.765	0.43
**103**	6.824	6.907	-0.08
**104**	6.658	6.910	-0.25
**110**	6.658	6.916	-0.26
**114**	7.328	6.958	0.37
**116**	6.813	6.910	-0.10
**123**	7.097	7.214	-0.12
**124**	6.000	5.435	0.56
**125**	5.699	5.432	0.27
**127**	6.189	5.910	0.28
**129**	6.009	6.031	-0.02
**149**	5.257	5.706	-0.45
**166**	6.127	6.056	0.07
**168**	6.000	6.120	-0.12
**171**	5.699	6.052	-0.35
**173**	6.000	5.871	0.13
**200**	6.183	5.758	0.42
**208**	6.407	6.036	0.37
**210**	6.390	6.636	-0.25
**211**	6.341	5.984	0.36
**212**	6.495	6.387	0.11
**213**	6.701	6.699	0.00
**215**	6.401	6.135	0.27
**225**	6.893	6.819	0.07
**230**	6.445	6.489	-0.04
**232**	6.544	6.042	0.50
**236**	6.000	6.052	-0.05
**238**	5.636	5.886	-0.25
**246**	7.009	6.652	0.36
**260**	6.155	6.281	-0.13
**271**	6.328	5.961	0.37
**276**	5.510	5.907	-0.40
**277**	5.577	5.975	-0.40
**280**	5.498	5.975	-0.48
**283**	5.842	5.961	-0.12
**290**	5.955	5.863	0.09
**291**	5.947	5.829	0.12
**293**	7.222	6.754	0.47

**294**	6.854	6.720	0.13
**295**	6.076	6.445	-0.37
**297**	5.959	6.395	-0.44
**301**	6.575	6.967	-0.39
**303**	6.606	6.882	-0.28
**308**	6.420	6.390	0.03
**309**	6.315	6.114	0.20
**313**	5.523	5.404	0.12
**315**	5.301	5.707	-0.41
**316**	5.222	5.707	-0.48
**317**	5.155	5.444	-0.29
**318**	5.222	5.403	-0.18
**320**	5.398	5.544	-0.15
**321**	5.432	5.387	0.05
**322**	5.398	5.567	-0.17
**326**	5.301	5.615	-0.31
**328**	5.523	5.714	-0.19
**330**	5.398	5.578	-0.18
**337**	6.842	6.434	0.41
**339**	7.081	6.697	0.38
**341**	5.301	5.309	-0.01
	**Test Set**		
**11**	6.530	6.774	-0.24
**28**	7.128	7.095	0.03
**29**	7.014	7.057	-0.04
**30**	7.404	7.325	0.08
**37**	7.537	7.568	-0.03
**44**	7.472	7.269	0.20
**45**	7.153	6.818	0.34
**48**	7.311	7.130	0.18
**50**	7.285	7.057	0.23
**62**	5.699	5.684	0.02
**67**	6.199	6.023	0.18
**80**	5.398	5.777	-0.38
**84**	6.097	6.130	-0.03
**87**	5.602	5.716	-0.11
**88**	5.770	5.898	-0.13
**93**	5.699	6.467	-0.77
**94**	6.577	6.465	0.11
**167**	5.301	5.831	-0.53
**203**	6.272	6.204	0.07
**204**	6.329	6.324	0.00
**209**	6.181	6.515	-0.33
**214**	6.290	6.734	-0.44
**222**	6.810	6.838	-0.03
**226**	6.810	6.654	0.16

**234**	5.991	6.448	-0.46
**239**	6.535	6.094	0.44
**242**	5.699	5.879	-0.18
**243**	6.133	5.916	0.22
**248**	6.264	6.612	-0.35
**249**	6.108	6.461	-0.35
**266**	6.203	6.227	-0.02
**274**	7.004	7.153	-0.15
**289**	6.032	5.879	0.15
**298**	6.672	6.382	0.29
**302**	6.650	6.951	-0.30
**307**	6.682	6.424	0.26
**310**	5.708	6.098	-0.39
**311**	5.854	6.065	-0.21
**312**	5.699	5.396	0.30
**314**	5.523	5.405	0.12
**319**	5.523	5.387	0.14
**323**	5.523	5.583	-0.06
**327**	5.301	5.575	-0.27
**329**	5.155	5.559	-0.40
**342**	5.222	5.311	-0.09
**343**	5.301	5.610	-0.31

References

[1] M. Mellado, C. Gonzalez, J. Mella, L.F. Aguilar, I. Celik, F. Borges, E. Uriarte, G. Delogu, D. Vina, M.J. Matos, Coumarin-Resveratrol-Inspired Hybrids as Monoamine Oxidase B Inhibitors: 3-Phenylcoumarin versus trans-6-Styrylcoumarin, Molecules. 27 (2022) 928. https://doi.org/10.3390/molecules27030928.

[2] M. Lorca, M. Faundez, C.D. Pessoa-Mahana, G. Recabarren-Gajardo, B. Diethelm-Varela, D. Millan, I. Celik, M. Mellado, I. Araque, J. Mella, J. Romero-Parra, Design of benzimidazoles, benzoxazoles, benzothiazoles and thiazolopyridines as leukotriene A4 hydrolase inhibitors through 3D-QSAR, docking and molecular dynamics, J. Serb. Chem. Soc. 88 (2023) 25–39. https://doi.org/10.2298/JSC220427068L.

[3] M. Erol, I. Celik, B.N. Saglik, A. Karayel, M. Mellado, J. Mella, Synthesis, molecular modeling, 3D-QSAR and biological evaluation studies of new benzimidazole derivatives as potential MAO-A and MAO-B inhibitors, J. Mol. Struct. 1265 (2022) 133444. https://doi.org/10.1016/j.molstruc.2022.133444.

[4] U.A. Çevik, I. Celik, J. Mella, M. Mellado, Y. Özkay, Z.A. Kaplancıklı, Design, Synthesis, and Molecular Modeling Studies of a Novel Benzimidazole as an Aromatase Inhibitor, ACS Omega. 7 (2022) 16152–16163. https://doi.org/10.1021/acsomega.2c01497.

[5] G. Klebe, U. Abraham, T. Mietzner, Molecular Similarity Indexes in a Comparative-Analysis (comsia) of Drug Molecules to Correlate and Predict Their Biological-Activity, J. Med. Chem. 37 (1994) 4130–4146. https://doi.org/10.1021/jm00050a010.

[6] M. Beus, D. Fontinha, J. Held, Z. Rajic, M. Prudencio, B. Zorc, Synthesis and antiplasmodial evaluation of novel mefloquine-based fumardiamides, Acta Pharm. 69 (2019) 233–248. https://doi.org/10.2478/acph-2019-0019.

[7] S. Aggarwal, D. Paliwa, D. Kaushik, G.K. Gupta, A. Kumar, Synthesis, Antimalarial Evaluation and SAR Study of Some 1,3,5-Trisubstituted Pyrazoline Derivatives, Lett. Org. Chem. 16 (2019) 807–817. https://doi.org/10.2174/1570178616666190212145754.

[8] D. Saini, S. Jain, A. Kumar, N. Jain, Synthesis and Antimalarial Potential of Some Novel Quinoline-Pyrazolopyridine Derivatives, EXCLI J. 15 (2016) 730–737. https://doi.org/10.17179/excli2016-677.

[9] K.V. Sashidhara, S.R. Avula, G.R. Palnati, S.V. Singh, K. Srivastava, S.K. Puri, J.K. Saxena, Synthesis and in vitro evaluation of new chloroquine-chalcone hybrids against chloroquine-resistant strain of Plasmodium falciparum, Bioorg. Med. Chem. Lett. 22 (2012) 5455–5459. https://doi.org/10.1016/j.bmcl.2012.07.028.

[10] A. Mishra, H. Batchu, K. Srivastava, P. Singh, P.K. Shukla, S. Batra, Synthesis and evaluation of new diaryl ether and quinoline hybrids as potential antiplasmodial and antimicrobial agents, Bioorg. Med. Chem. Lett. 24 (2014) 1719–1723. https://doi.org/10.1016/j.bmcl.2014.02.044.

[11] S. Jain, A. Kumar, D. Saini, Novel arylidene derivatives of quinoline based thiazolidinones: Synthesis, in vitro, in vivo and in silico study as antimalarials, Exp. Parasitol. 185 (2018) 107–114. https://doi.org/10.1016/j.exppara.2018.01.015.

[12] S.P. Prajapati, N.K. Kaushik, M. Zaveri, D. Mohanakrishanan, N. Kawathekar, D. Sahal, Synthesis, characterization and antimalarial evaluation of new beta-benzoylstyrene derivatives of acridine, Arab. J. Chem. 10 (2017) S274–S280. https://doi.org/10.1016/j.arabjc.2012.07.033.

[13] K. Singh, H. Kaur, K. Chibale, J. Balzarini, S. Little, P.V. Bharatam, 2-Aminopyrimidine based 4-aminoquinoline anti-plasmodial agents. Synthesis, biological activity, structure-activity relationship and mode of action studies, Eur. J. Med. Chem. 52 (2012) 82–97. https://doi.org/10.1016/j.ejmech.2012.03.007.

[14] N.M. Shah, M.P. Patel, R.G. Patel, New N-arylamino biquinoline derivatives: Synthesis, antimicrobial, antituberculosis, and antimalarial evaluation, Eur. J. Med. Chem. 54 (2012) 239–247. https://doi.org/10.1016/j.ejmech.2012.05.004.

[15] M. Tukulula, S. Little, J. Gut, P.J. Rosenthal, B. Wan, S.G. Franzblau, K. Chibale, The design, synthesis, in silico ADME profiling, antiplasmodial and antimycobacterial evaluation of new arylamino quinoline derivatives, Eur. J. Med. Chem. 57 (2012) 259–267. https://doi.org/10.1016/j.ejmech.2012.08.047.

[16] J. Ramirez-Prada, S.M. Robledo, I.D. Velez, M. del Pilar Crespo, J. Quiroga, R. Abonia, A. Montoya, L. Svetaz, S. Zacchino, B. Insuasty, Synthesis of novel quinoline based 4,5 dihydro-1H pyrazoles as potential anticancer, antifungal, antibacterial and antiprotozoal agents, Eur. J. Med. Chem. 131 (2017) 237–254. https://doi.org/10.1016/j.ejmech.2017.03.016.

[17] G. Bentzinger, W. De Souza, C. Mullie, P. Agnamey, A. Dassonville-Klimpt, P. Sonnet, Asymmetric synthesis of new antimalarial aminoquinolines through Sharpless aminohydroxylation, Tetrahedron-Asymmetry. 27 (2016) 1–11. https://doi.org/10.1016/j.tetasy.2015.11.003.

[18] K.V. Sashidhara, M. Kumar, R.K. Modukuri, R.K. Srivastava, A. Soni, K. Srivastava, S.V. Singh, J.K. Saxena, H.M. Gauniyal, S.K. Puri, Antiplasmodial activity of novel keto-enamine chalcone-chloroquine based hybrid pharmacophores, Bioorg. Med. Chem. 20 (2012) 2971–2981. https://doi.org/10.1016/j.bmc.2012.03.011.

[19] A. Barteselli, S. Parapini, N. Basilico, D. Mommo, A. Sparatore, Synthesis and evaluation of the antiplasmodial activity of novel indeno[2,1-c]quinoline derivatives, Bioorg. Med. Chem. 22 (2014) 5757–5765. https://doi.org/10.1016/j.bmc.2014.09.040.

[20] R. Sharma, R. Kumar, R. Kumar, P. Upadhyay, D. Sahal, U. Sharma, Rh(III)-Catalyzed C(8)-H Functionalization of Quinolines via Simultaneous C-C and C-O Bond Formation: Direct Synthesis of Quinoline Derivatives with Antiplasmodial Potential, J. Org. Chem. 83 (2018) 12702–12710. https://doi.org/10.1021/acs.joc.8b02042.

[21] H. Li, W. Sun, X. Huang, X. Lu, P.R. Patel, M. Kim, M.J. Orr, R.M. Fisher, T.Q. Tanaka, J.C. McKew, A. Simeonov, P.E. Sanderson, W. Zheng, K.C. Williamson, W. Huang, Efficient Synthesis of 1,9-Substituted Benzo[h][1,6]naphthyridin-2(1H)-ones and Evaluation of their Plasmodium falciparum Gametocytocidal Activities, ACS Comb. Sci. 19 (2017) 748–754. https://doi.org/10.1021/acscombsci.7b00119.

[22] S. Dana, S.K. Keshri, J. Shukla, K.S. Vikramdeo, N. Mondal, P. Mukhopadhyay, S.K. Dhar, Design, Synthesis and Evaluation of Bifunctional Acridinine-Naphthalenediimide Redox-Active Conjugates as Antimalarials, ACS Omega. 1 (2016) 318–333. https://doi.org/10.1021/acsomega.6b00060.
[23] V.R. Dola, A. Soni, P. Agarwal, H. Ahmad, K.S.R. Raju, M. Rashid, M. Wahajuddin, K. Srivastava, W. Haq, A.K. Dwivedi, S.K. Puri, S.B. Katti, Synthesis and Evaluation of Chirally Defined Side Chain Variants of 7-Chloro4-Aminoquinoline To Overcome Drug Resistance in Malaria Chemotherapy, Antimicrob. Agents Chemother. 61 (2017) e01152-16. https://doi.org/10.1128/AAC.01152-16.
[24] J. Guillon, A. Cohen, C. Boudot, A. Valle, V. Milano, R.N. Das, A. Gudin, S. Moreau, L. Ronga, S. Savrimoutou, M. Demourgues, E. Reviriego, S. Rubio, S. Ferriez, P. Agnamey, C. Pauc, S. Moukha, P. Dozolme, S. Da Nascimento, P. Laumaille, A. Bouchut, N. Azas, J.-L. Mergny, C. Mullie, P. Sonnet, B. Courtioux, Design, synthesis, and antiprotozoal evaluation of new 2,4-bis[(substituted-aminomethyl)phenyl]quinoline, 1,3-bis[(substituted-aminomethyl)phenyl]isoquinoline and 2,4-bis[(substituted-aminomethyl)phenyl]quinazoline derivatives, J. Enzym. Inhib. Med. Chem. 35 (2020) 432–459. https://doi.org/10.1080/14756366.2019.1706502.
[25] X.-M. Yu, F. Ramiandrasoa, L. Guetzoyan, B. Pradines, E. Quintino, D. Gadelle, P. Forterre, T. Cresteil, J.-P. Mahy, S. Pethe, Synthesis and Biological Evaluation of Acridine Derivatives as Antimalarial Agents, ChemMedChem. 7 (2012) 587–605. https://doi.org/10.1002/cmdc.201100554.
[26] A. Ramazani, B. Khosravani, J. Taran, A. Ramazani, Evaluation of Novel alpha-(Acyloxy)-alpha-(Quinolin-4-yl) Acetamides as Antiplasmodial Agents, Iran. J. Pharm. Res. 16 (2017) 924–928.
[27] M. Beus, D. Fontinha, J. Held, Z. Rajic, L. Uzelac, M. Kralj, M. Prudencio, B. Zorc, Primaquine and Chloroquine Fumardiamides as Promising Antiplasmodial Agents, Molecules. 24 (2019) 2812. https://doi.org/10.3390/molecules24152812.
